# Stimulus-responsive metallocenes: a photo/thermal switch enabled by the perfluorinated Cp* ligand†

**DOI:** 10.1039/d5sc02784e

**Published:** 2025-05-27

**Authors:** Robin Sievers, Nick Hartmann, Paulin S. Riemann, Tim-Niclas Streit, Moritz Malischewski

**Affiliations:** a Freie Universität Berlin, Institut für Chemie und Biochemie – Anorganische Chemie Fabeckstraße 34/36 14195 Berlin Germany moritz.malischewski@fu-berlin.de

## Abstract

The electron-deficient ferrocene [Fe(C_5_H_5_)(C_5_(CF_3_)_5_)] is complemented by the synthesis and full characterisation of the analogous bench-stable ruthenocene [Ru(C_5_H_5_)(C_5_(CF_3_)_5_)]. These complexes have been studied with respect to the substitution lability of the perfluorinated Cp* ligand under mild conditions. Photolysis of the metallocenes in MeCN converted the [C_5_(CF_3_)_5_]^−^ ligand into a weakly coordinating anion. This gave access to the highly reactive piano-stool complexes [M(C_5_H_5_)(MeCN)_3_][C_5_(CF_3_)_5_] (M = Fe, Ru). The unstable iron half-sandwich complex dismutates under formation of [Fe(C_5_H_5_)_2_] and [Fe(MeCN)_6_][C_5_(CF_3_)_5_]_2_. It was trapped by the chelating diphosphine DPPE and isolated as thermally stable [Fe(C_5_H_5_)(DPPE)(MeCN)][C_5_(CF_3_)_5_]. For [Ru(C_5_H_5_)(MeCN)_3_][C_5_(CF_3_)_5_] a thermally induced backreaction to ruthenocene is observed. This represents the first example of a reversible dissociation and recoordination of a cyclopentadienyl ligand, initiated by light and heat.

## Abstract 2

The [C_5_(CF_3_)_5_]^−^ ligand in metallocenes (M = Fe, Ru) is substituted under irradiation in MeCN. While the iron complex scrambles, the cationic ruthenium complex can be converted back into the metallocene upon heating.

## Graphical abstract

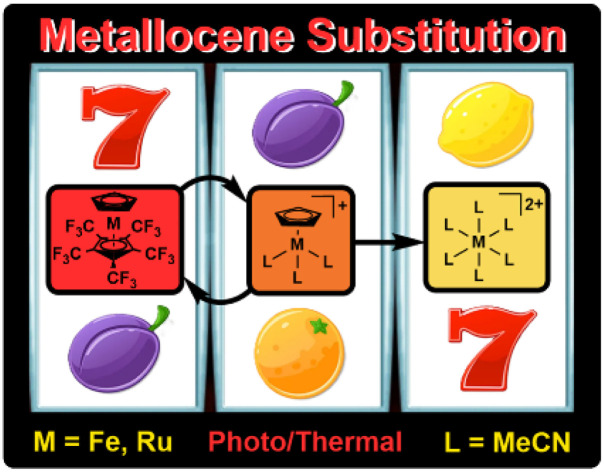

## Introduction

**Scheme 1:**
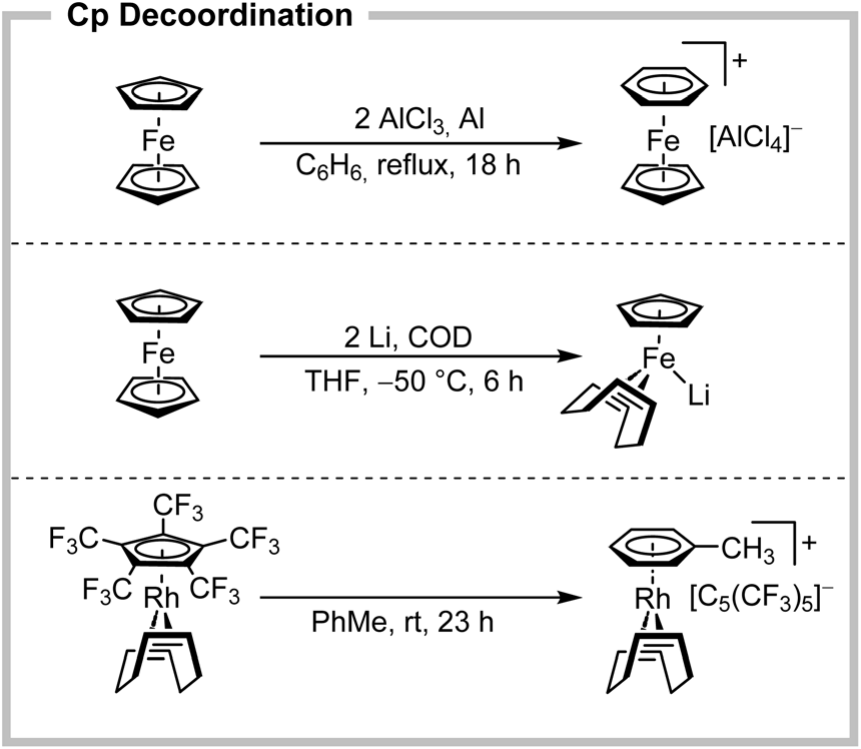
Synthetic approaches towards Cp decoordination by Lewis acids (top) or reduction (centre) in metallocenes and substitution of the electron-deficient perfluorinated Cp* by electron-rich arenes (bottom).

Since the discovery of ferrocene in 1951,^1^ the group of metallocenes has developed as a fundamental part of organometallic chemistry. Today, due to their valuable properties, they are manifested in various fields, ranging from (bio)organometallics to catalysis, polymers and material science. In particular for the group 8 metallocenes, including the iconic ferrocene, this is not least due to their exceptional chemical stability combined with functional tunability.^2,3^ This has led to extensive studies of different substitution patterns on the cyclopentadienyl (Cp) ligands, yielding today more than 22 000 structure hits according to the Cambridge Crystallographic Data Centre (CCDC) for ferrocenes alone.^4^ However, almost none of these functionalization involves the metal–Cp bond itself. The explanation for this lies in the extraordinarily high bond dissociation energy (BDE), which is more than 1000 kJ mol^−1^ for [Fe(C_5_H_5_)_2_],^5,6^ leaving the notion of metallocenes as synthetic dead ends with respect to their inert metal–Cp bond. In fact, the removal of Cp ligands in metallocenes by arenes was introduced as early as 1963 by the Nesmeyanov group (Scheme 1, top),^7^ laying the foundation for seminal works as those from Astruc.^3,8^ However, these substitutions required very harsh conditions, such as strong Lewis acids, high temperatures and an excess of reagents. In addition, these reactions are often associated with strict substrate limitations and low yields and generally rather resemble metallocene decomposition than substitution reactions.

Besides highly strained and therefore activated metallocenophanes,^9^ the only alternative for metallocene cleavage is represented by highly reductive conditions (Scheme 1, centre). Reaction with lithium in the presence of olefins as better acceptor ligands allows for the elimination of LiCp to give the corresponding reduced iron(0) or iron(−ii) species.^10^ Although the Cp ligand is selectively displaced here, this reaction primarily involves a change in oxidation state and only yields highly air-sensitive products. Thus, even today, the ability to manipulate the metal–Cp bond in metallocenes is highly limited to very specific conditions. However, the perfluorinated Cp* anion is known since 1980, with different synthetic routes to it, reported by Lemal and, more recently, by Chambers *et al.*^11,12^ In 2022, we introduced it to coordination chemistry by the preparation of several transition metal complexes, such as [Rh(COD)(C_5_(CF_3_)_5_)].^13,14^ The electron-deficient [C_5_(CF_3_)_5_]^−^ exhibits an extraordinarily weak binding character according to DFT calculations and in direct comparison to regular Cp ligands. This finding is in stark contrast to directly fluorinated Cp ligands [C_5_F_5_]^−^, presented in seminal works of Hughes and Sünkel, due to the absence of any conjugative donor effects of the CF_3_-groups.^15^ In the case of [Rh(COD)(C_5_R_5_)] (R = H, CF_3_) the bond energies differed by an impressive value of 259 kJ mol^−1^. This was confirmed experimentally, as [Rh(COD)(C_5_(CF_3_)_5_)] underwent an unexpected quantitative (and reversible) substitution in toluene towards [Rh(COD)(PhMe)][C_5_(CF_3_)_5_] (Scheme 1, bottom),^13^ which was subsequently demonstrated by the substitution with fluorinated pyridines and triphenylpnictogens.^16^ The unique property of the perfluorinated Cp* to act either as a ligand or as a weakly coordinating anion (WCA) allows these clean conversions under very mild conditions,^17^ raising the question whether the substitution lability of [Rh(COD)(C_5_(CF_3_)_5_)] could be transferred to the challenging substrate class of metallocenes. Here, electron deficiency induced lability resembles an unprecedented approach (previously only observed by mass spectrometry)^18^ for metallocene chemistry. This would potentially open up new synthetic pathways and applications and change the perception of the mostly inert metal–Cp bond.

## Results and discussion

**Scheme 2:**
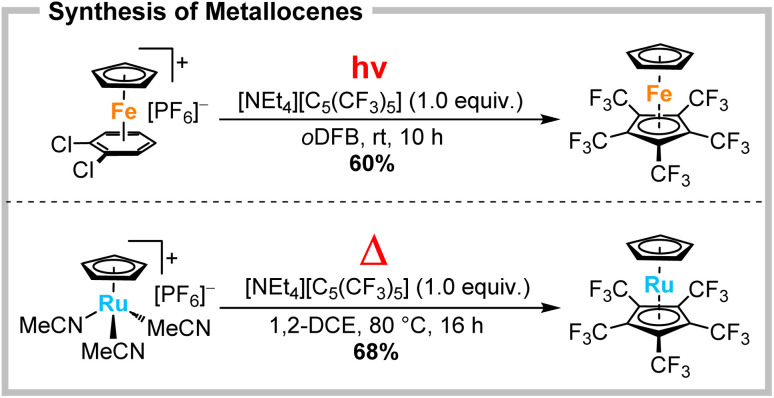
Synthesis of the extremely electron-poor metallocenes [M(C_5_H_5_)(C_5_(CF_3_)_5_)] (M = Fe, Ru) by photolytic (top) and thermal (bottom) ligand substitution.

Recently, we demonstrated the synthesis of the electron-deficient ferrocene [Fe(C_5_H_5_)(C_5_(CF_3_)_5_)] by a photolytically induced arene displacement of [Fe(C_5_H_5_)(*o*DCB)][PF_6_] (Scheme 2, top).^19^ Now, the analogous ruthenocene [Ru(C_5_H_5_)(C_5_(CF_3_)_5_)] is reported. Due to the reduced photolability of ruthenium arene complexes, this metallocene can only be obtained by a thermally induced ligand substitution of [Ru(C_5_H_5_)(MeCN)_3_][PF_6_] with a yield of 68% (Scheme 2, bottom). Like its iron counterpart, the colourless solid [Ru(C_5_H_5_)(C_5_(CF_3_)_5_)] was found to be completely bench-stable and exhibited a highly unusual solubility for metallocenes in perfluorocarbons. The colour shift with respect to non-fluorinated ruthenocenes (yellow) results from spin-allowed ^1^A_1g_ → ^1^E_1g_ and ^1^A_1g_ → ^1^E_2g_ d–d transitions.^18,20^ In combination with an energetically enhanced HOMO–LUMO gap a hypsochromic shift is observed, giving an absorption maximum within the UV region. ^19^F and ^1^H NMR spectroscopy revealed one sharp singlet at −50.6 and 5.24 ppm, respectively. The latter indicates a strong high-field shift compared to normal ruthenocene at 5.52 ppm.^21^ Single crystals were obtained from solutions of perfluorohexanes by slow cooling to −70 °C. [Ru(C_5_H_5_)(C_5_(CF_3_)_5_)] crystallizes in the monoclinic *P*2_1_/*c* space group and exhibits coplanar η^5^-coordination of both Cp ligands (see Fig. S40†). Notably, [C_5_(CF_3_)_5_]^−^ is slightly closer to the ruthenium centre with 1.817(1) Å, compared to the electron richer [C_5_H_5_]^−^ with 1.823(1) Å ([Ru(C_5_H_5_)_2_]: 1.842 Å).^22^ This trend is even stronger pronounced for [Fe(C_5_H_5_)(C_5_(CF_3_)_5_)] and could be easily mistaken as a stronger metal–Cp interaction for the perfluorinated Cp*. Actually this is properly explained due to a significant dipole and push–pull nature of the metallocenes, induced by the extreme electron withdrawal of the CF_3_-groups.^19^ The strength of the metal–Cp interactions was evaluated using DFT (B3LYP-D3BJ/def2TZVP) by calculating the energy change for the combination of cationic metal fragments and the corresponding anionic cyclopentadienyl ligands. The two different metal–Cp bonds in [Ru(C_5_H_5_)(C_5_(CF_3_)_5_)] can formally be described by the interaction of diamagnetic [Ru(C_5_H_5_)]^+^ and [C_5_(CF_3_)_5_]^−^ (671 kJ mol^−1^) or combination of [Ru(C_5_(CF_3_)_5_)]^+^ and [C_5_H_5_]^−^ (1108 kJ mol^−1^). For [Fe(C_5_H_5_)(C_5_(CF_3_)_5_)] this analysis is complicated by the energetic preference of the quintet state for the cationic half-sandwich complexes. Interaction of paramagnetic [Fe(C_5_H_5_)]^+^ and [C_5_(CF_3_)_5_]^−^ (589 kJ mol^−1^) or combination of [Fe(C_5_(CF_3_)_5_)]^+^ and [C_5_H_5_]^−^ (1013 kJ mol^−1^) indicate weaker M–Cp bonds compared to the ruthenium compound. For the energetically higher diamagnetic iron-based fragments the corresponding values would be 696 and 1134 kJ mol^−1^. In all cases this simple model shows that the strength of interaction between the metal and the [C_5_(CF_3_)_5_]^−^ is much lower than the interaction with [C_5_H_5_]^−^. These diminished values are due to the significantly decreased σ- and π-donor capabilities (but enhanced δ-acceptor ability) of [C_5_(CF_3_)_5_]^−^ compared to regular [C_5_H_5_]^−^.^13,19^ Nevertheless, the comparison of the corresponding value for the metal–[C_5_(CF_3_)_5_] interaction in [Rh(COD)(C_5_(CF_3_)_5_)]^13^ (500 kJ mol^−1^) demonstrates the significantly higher bond energy in metallocenes, making them more challenging substrates in substitution reactions.

**Scheme 3:**
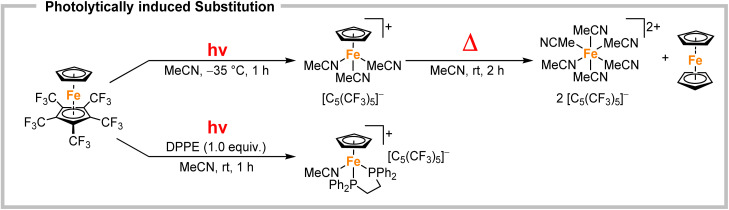
Photolytic substitution of the perfluorinated Cp* in [Fe(C_5_H_5_)(C_5_(CF_3_)_5_)] towards the thermally labile [Fe(C_5_H_5_)(MeCN)_3_][C_5_(CF_3_)_5_] (top) and stable [Fe(C_5_H_5_)(DPPE)(MeCN)][C_5_(CF_3_)_5_] (bottom).

**Fig. 1:**
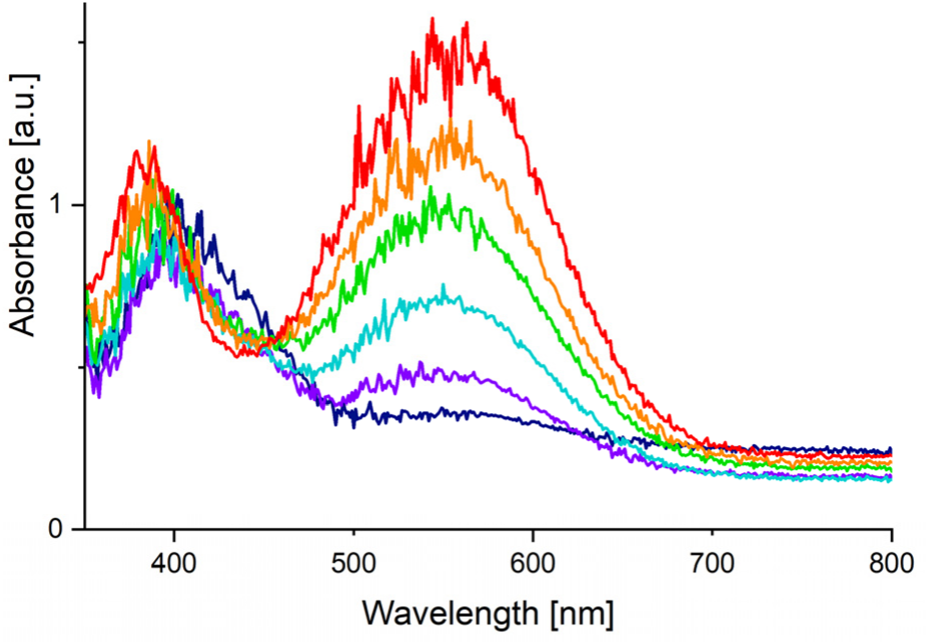
UV/VIS spectra (MeCN, −35 °C) of [Fe(C_5_H_5_)(C_5_(CF_3_)_5_)] under UV irradiation after 0 min (dark blue), 10 min (violet), 20 min (light blue), 30 min (green), 40 min (orange), 75 min (red).

**Fig. 2:**
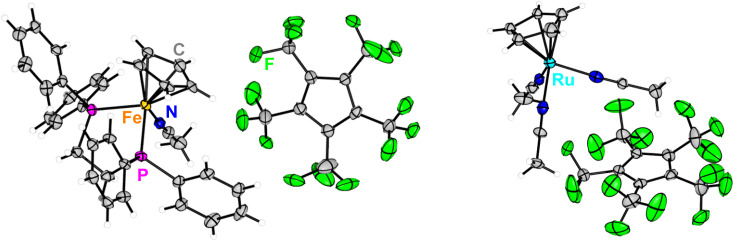
Molecular structure in solid state of [Ru(C_5_H_5_)(DPPE)(MeCN)][C_5_(CF_3_)_5_]·2CH_2_Cl_2_ (left) and [Ru(C_5_H_5_)(MeCN)_3_][C_5_(CF_3_)_5_]·CH_2_Cl_2_ (right). Solvent molecules and disorder are omitted for clarity. Ellipsoids are depicted with 50% probability level. Color code: white-hydrogen, grey-carbon, green-fluorine, orange-iron, deep blue-nitrogen, purple-phosphorus, light blue-ruthenium.

The substitution lability was first investigated by UV irradiation of the metallocenes in coordinating solvents. While nonstrained metallocenes are generally completely inert under such conditions, [Fe(C_5_H_5_)(C_5_(CF_3_)_5_)] underwent a fast conversion in MeCN (Scheme 3, top). When irradiated at −35 °C, a deep purple solution was obtained within 1 h. Low temperature NMR spectroscopy showed a significant shift in the ^19^F NMR spectrum of [C_5_(CF_3_)_5_]^−^ towards −51.0 ppm, demonstrating its substitution and conversion to a WCA. The still coordinated ligand [C_5_H_5_]^−^ showed a strong high-field shift in the ^1^H NMR spectrum at 3.93 ppm, indicating the formation of a cationic species. The intense colour transition was also monitored by low temperature UV/VIS spectroscopy with a gradual shift of the absorption maximum from 407 nm (for [Fe(C_5_H_5_)(C_5_(CF_3_)_5_)]) to 550 nm (Fig. 1). The release of [C_5_(CF_3_)_5_]^−^ together with the UV/VIS and ^1^H NMR shifts strongly suggest the formation of the thermally unstable piano-stool complex [Fe(C_5_H_5_)(MeCN)_3_][C_5_(CF_3_)_5_] with an almost quantitative conversion (according to NMR spectroscopy).^23^ This result impressively demonstrates the ability of the perfluorinated Cp* to facilitate an unprecedented substitution reaction by its extreme electron withdrawal within a metallocene. Unfortunately, the substitution product could not be isolated, but this is more likely explained by the labile nature of [Fe(C_5_H_5_)(MeCN)_3_]^+^ complexes themselves.^24^ Upon warming to room temperature the purple colour of [Fe(C_5_H_5_)(MeCN)_3_][C_5_(CF_3_)_5_] disappears completely and a yellow solution is obtained within 2 h, as shown by another time-dependent UV/VIS spectrum and a hypsochromic shift of the absorption maximum (see Fig. S38†). Instead of a possible recoordination of [C_5_(CF_3_)_5_]^−^ and reversibility towards [Fe(C_5_H_5_)(C_5_(CF_3_)_5_)], dismutation is observed. This results in the quantitative and equimolar formation of ferrocene [Fe(C_5_H_5_)_2_] and the dicationic solvate complex [Fe(MeCN)_6_][C_5_(CF_3_)_5_]_2_ which was confirmed by NMR spectroscopy (Scheme 3, top).^12^ In a similar experiment [Fe(C_5_H_5_)(C_5_(CF_3_)_5_)] and 1,2-bis(diphenylphosphino)ethane (DPPE) were irradiated in MeCN for 1 h to give a deep red solution that persists at room temperature (Scheme 3, bottom). Isolation and full characterisation of the red solid revealed the quantitative formation of [Fe(C_5_H_5_)(DPPE)(MeCN)][C_5_(CF_3_)_5_].^23^ In addition to the expected DPPE and MeCN resonances, ^1^H NMR spectroscopy showed a high field shift of [C_5_H_5_]^−^ towards 4.30 ppm. The ^19^F NMR spectrum shows a decoordinated and ionic [C_5_(CF_3_)_5_]^−^ with a chemical shift of −50.6 ppm. Single crystals suitable for XRD were obtained by slow cooling of a solution in *n*-pentane/CH_2_Cl_2_ to −70 °C. [Fe(C_5_H_5_)(DPPE)(MeCN)][C_5_(CF_3_)_5_]·2CH_2_Cl_2_ crystallized in the monoclinic *Pc* space group and revealed separated ions, with [C_5_(CF_3_)_5_]^−^ transformed into a WCA (Fig. 2, left). Thus, the demonstrated photolability of ferrocene [Fe(C_5_H_5_)(C_5_(CF_3_)_5_)] is emphasised by this quantitative substitution to a stable product. Upon heating (up to 80 °C), solutions of [Fe(C_5_H_5_)(DPPE)(MeCN)][C_5_(CF_3_)_5_] showed no decomposition or reversibility.

**Scheme 4:**
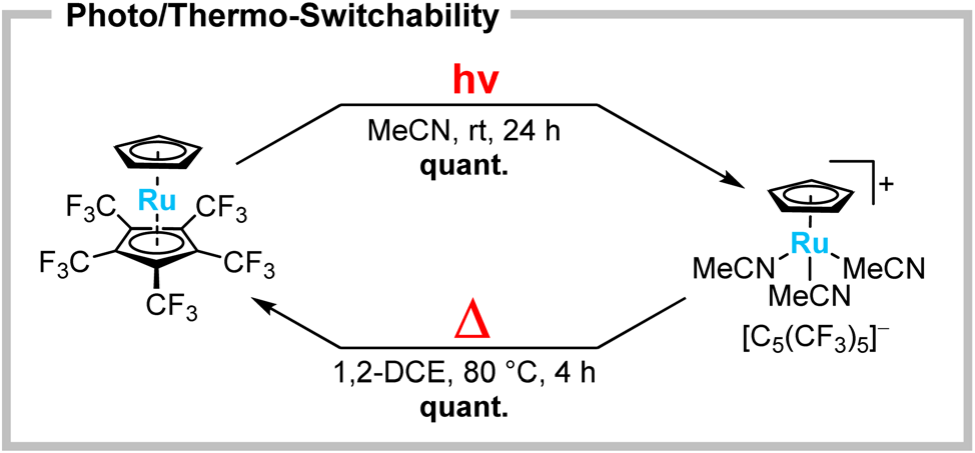
Photolytic substitution of the perfluorinated Cp* in [Ru(C_5_H_5_)(C_5_(CF_3_)_5_)] towards [Ru(C_5_H_5_)(MeCN)_3_][C_5_(CF_3_)_5_] (top) and its thermally induced reversibility (bottom).

The heavier homologues of group 8 metallocenes are generally known to form stronger metal–ligand bonds, resulting in even more challenging substrates for substitution,^5^ whereas the desired product scaffolds are thermally stable and less prone to scrambling reactions.^25,26^ When ruthenocene [Ru(C_5_H_5_)(C_5_(CF_3_)_5_)] is irradiated in MeCN at room temperature, decoordination of the perfluorinated Cp* ligand is again observed (Scheme 4, top). However, in comparison to the analogous ferrocene, the reaction is significantly slower, reaching full conversion only after 24 h. In the case of ruthenium, the product appeared to be indefinitely stable in solution and could even be isolated as a yellow solid. The ^1^H NMR spectrum showed a significant high-field shift of [C_5_H_5_]^−^ towards 4.24 ppm and the presence of solvate MeCN, due to a singlet at 2.29 ppm. ^19^F NMR spectra showed a shift of the [C_5_(CF_3_)_5_]^−^ singlet towards −50.6 ppm. This suggests the formation of the substitution product [Ru(C_5_H_5_)(MeCN)_3_][C_5_(CF_3_)_5_].^25^ Single crystals suitable for XRD were obtained by slow cooling of a solution in *n*-pentane/CH_2_Cl_2_ to −70 °C. [Ru(C_5_H_5_)(MeCN)_3_][C_5_(CF_3_)_5_]·CH_2_Cl_2_ crystallized in the monoclinic *P*2_1_ space group, confirming the photolytic conversion of the [C_5_(CF_3_)_5_]^−^ ligand to a WCA (Fig. 2, right). The herein increased reaction time indicates an energetic preference for the neutral ruthenocene over the analogous ferrocene substitution. This raised the question of the potential reversibility of the substitution. Indeed, when solutions of the piano-stool complex [Ru(C_5_H_5_)(MeCN)_3_][C_5_(CF_3_)_5_] are heated in solutions (except MeCN), such as 1,2-dichloroethane (1,2-DCE), the quantitative back-reaction towards ruthenocene [Ru(C_5_H_5_)(C_5_(CF_3_)_5_)] is observed within a few hours (Scheme 4, bottom). This unique reactivity for metallocenes can be considered as a photo/thermo-switchability, allowing the perfluorinated Cp* to be transformed between ligand and WCA.

While metallocenes are usually known as synthetic dead ends with respect to their metal–Cp framework, the substitution of [M(C_5_H_5_)(C_5_(CF_3_)_5_)] (M = Fe, Ru) demonstrates an unprecedented exception from this. Since the only alternatives within metallocene chemistry are the use of strongly Lewis acidic or reductive conditions, the electron deficiency of the perfluorinated Cp* resembles a hitherto unknown approach, namely irradiation in the Lewis basic solvent MeCN. The corresponding cationic piano-stool complexes are not only treated as highly reactive and valuable synthetic precursors,^27^ but also find application as potent catalysts (*e.g.* C–C-coupling, isomerisation, cycloaddition) as demonstrated by Trost and other groups.^28^ Not only the possibility of a photolytic *in situ* activation of a so far unreactive, bench-stable metallocene, but particularly the combination with its reformation under heating may therefore inspire for unique (biphasic) applications in the future.

## Conclusions

In conclusion, the synthesis and full characterisation of the electron deficient ruthenocene [Ru(C_5_H_5_)(C_5_(CF_3_)_5_)] is presented, which supplements the analogue ferrocene [Fe(C_5_H_5_)(C_5_(CF_3_)_5_)].^19^ Contrary to the notion of an inert metal–Cp bond, both complexes were examined regarding a metallocene-unique substitution lability of the perfluorinated Cp* ligand. Photolysis of metallocenes [M(C_5_H_5_)(C_5_(CF_3_)_5_)] (M = Fe, Ru) in MeCN yielded the corresponding piano-stool complexes [M(C_5_H_5_)(MeCN)_3_][C_5_(CF_3_)_5_] and [Fe(C_5_H_5_)(DPPE)(MeCN)][C_5_(CF_3_)_5_] by an unexpected substitution reaction in quantitative yield. These results prove that an extreme electron deficiency can facilitate an unprecedented substitution lability in metallocenes. Furthermore, for [Ru(C_5_H_5_)(MeCN)_3_][C_5_(CF_3_)_5_] a thermally induced reversibility towards its ruthenocene was demonstrated, introducing a photo/thermo-switchability being unique for metallocenes.

## Data availability

Data supporting this manuscript is available within the ESI† and available on request.

## Author contributions

RS: investigation, formal analysis, writing (original draft); NH: investigation; PSR: investigation; TNS: formal analysis; MM: conceptualization, supervision, project administration, writing (review and editing).

## Conflicts of interest

There are no conflicts to declare.

## Supplementary Material

SC-OLF-D5SC02784E-s001

SC-OLF-D5SC02784E-s002

## References

[cit1] Kealy T. J., Pauson P. L. (1951). Nature.

[cit2] Astruc D. (2017). Eur. J. Inorg. Chem..

[cit3] Astruc D. (2023). Chem. Commun..

[cit4] Groom C. R., Bruno I. J., Lightfood M. P., Ward S. C. (2016). Acta Crystallogr..

[cit5] Swart M. (2007). Inorg. Chim. Acta.

[cit6] Klopper W., Lüthi H. P. (1996). Chem. Phys. Lett..

[cit7] Nesmeyanov A. N., Vol'kenau N. A., Shilovtseva L. S. (1969). Izv. Akad. Nauk SSSR, Ser. Khim..

[cit8] Astruc D., Hamon J. R., Althoff G., Román E., Batail P., Michaud P., Mariot J. P., Varret F., Cozak D. (1979). J. Am. Chem. Soc..

[cit9] Tanabe M., Vandermeulen G. W. M., Chan W. Y., Cyr P. W., Vanderark L., Rider D. A., Manners I. (2006). Nat. Mater..

[cit10] Jonas K., Schieferstein L. (1979). Angew. Chem..

[cit11] Laganis E. D., Lemal D. M. (1980). J. Am. Chem. Soc..

[cit12] Chambers R. D., Gray W. K., Vaughan J. F. S., Korn S. R., Médebielle M., Batsanov A. S., Lehmann C. W., Howard J. A. K. (1997). J. Chem. Soc., Perkin Trans. 1.

[cit13] Sievers R., Sellin M., Rupf S. M., Parche J., Malischewski M. (2022). Angew. Chem., Int. Ed..

[cit14] Sievers R., Parche J., Kub N. G., Malischewski M. (2023). Synlett.

[cit15] Curnow O. J., Hughes R. P. (1992). J. Am. Chem. Soc..

[cit16] Parche J., Rupf S. M., Sievers R., Malischewski M. (2023). Dalton Trans..

[cit17] Sievers R., Kub N. G., Streit T.-N., Rupf S. M., Malischewski M. (2025). Chem.–Eur. J..

[cit18] Yamaguchi Y., Ding W., Sanderson C. T., Borden M. L., Morgan M. J., Kutal C. (2007). Coord. Chem. Rev..

[cit19] Sievers R., Kub N. G., Streit T.-N., Reimann M., Thiele G., Kaupp M., Malischewski M. (2025). Angew. Chem., Int. Ed..

[cit20] Sohn Y. S., Hendrickson D. N., Gray H. B. (1971). J. Am. Chem. Soc..

[cit21] Watanabe M., Sano H. (1991). Chem. Lett..

[cit22] Hardgrove G. L., Templeton D. H. (1959). Acta Crystallogr..

[cit23] Gill T. P., Mann K. R. (1983). Inorg. Chem..

[cit24] Darchsen A. (1986). J. Organomet. Chem..

[cit25] Gill T. P., Mann K. R. (1982). Organometallics.

[cit26] Freedman D. A., Gill T. P., Blough A. M., Koefod R. S., Mann K. R. (1997). Inorg. Chem..

[cit27] Matsuo Y., Tahara K., Fujita T., Nakamura E. (2009). Angew. Chem., Int. Ed..

[cit28] Trost B. M., Toste F. D. (2000). J. Am. Chem. Soc..

