# Supplementary material for: Stimulus-responsive metallocenes: a photo/thermal switch enabled by the perfluorinated Cp* ligand
Source: Chem Sci. 2025 May 27;16(26):11949–53. doi: 10.1039/d5sc02784e (PMC12131068; doi:10.1039/d5sc02784e)
Supplement: SC-016-D5SC02784E-s001 [file SC-016-D5SC02784E-s001.pdf]

Supplementary Information (SI) for Chemical Science.
This journal is © The Royal Society of Chemistry 2025

Stimulus-responsive metallocenes: a photo/thermal switch enabled by the perfluorinated Cp* ligand

Robin Sievers, Nick Hartmann, Paulin S. Riemann, Tim-Niclas Streit, Moritz Malischewski*

R. Sievers, N. Hartmann, P. S. Riemann, T.-N. Streit, Dr. M. Malischewski

Institute of Chemistry and Biochemistry

Freie Universität Berlin

Fabeckstr. 34/36, 14195 Berlin, Germany

E-mail: moritz.malischewski@fu-berlin.de

Contents

General Information 2
Synthetic Procedures 4
NMR Spectra 9
IR Spectra 26
UV/VIS Spectra 28
Crystallographic Data 29
Density functional theory (DFT) calculations 34
References 40

General Information

Reactions and workups sensitive to air were performed in previously heated glassware under an atmosphere of argon using standard Schlenk techniques and an oil pump vacuum of 10^{-3} mbar. Room temperature (rt) refers to 25 °C. The addition of liquid reagents and solvents was done by using threefold argon-flushed disposable syringes and septa, while solids were added in argon stream. Low temperature reactions were performed in a cooled ethanol-bath. Glassware was cleaned by storing in a potassium hydroxide bath for several days, rinsed with diluted hydrochloric acid and doubly deionized water and dried at 150 °C.

Pressure reactions

The synthesis of $[NEt_4][C_5(CF_3)_5]$ involves high temperatures and highly volatile substances in a closed system. Hence, it must be assumed, that high pressures will be generated upon heating and advanced caution is required. Therefore, it is advisable to perform the reaction in a separate and properly closed fume hood. The thick-walled glass reaction vessel should not be opened and if possible, not even touched until the reaction is finished and has reached rt.

Solvents, reagents and materials

Anhydrous CH_2Cl_2, MeCN and *n*-pentane were obtained from the solvent system FMBRAUN MB SPS-800 and stored over activated 3 Å molecular sieves. Anhydrous *o*DCB, *o*DFB and 1,2-DCE were used as purchased and stored over activated 3 Å molecular sieves. Deuterated solvents CD_2Cl_2, d_3-MeCN and $(CD_3)_2CO$ were used as purchased and stored over activated 3 Å molecular sieves. Sulfolane was heated at 60 °C for at least 24 h over activated 3 Å molecular sieves and additionally 1 h in high vacuum prior to use. 18-crown-6 was heated at 80 °C for 2 h in high vacuum prior to use. All other solvents and commercially available reagents were used without further purification. Photochemistry was performed with a blue LED (470 nm) from OSRAM mounted on a 10 cm^3 aluminum heat sink or a 200 W low-pressure mercury vapor lamp (UV).

Nuclear magnetic resonance (NMR) spectroscopy

NMR spectra were measured on a JEOL ECX 400 (400 MHz) or a Varian INOVA 600 (600 MHz) in the reported deuterated solvents CD_2Cl_2, d_3-MeCN and $(CD_3)_2CO$. All given chemical shifts in ^{1}H NMR spectra are calibrated on the resonance signals of $CDHCl_2$ contained in CD_2Cl_2 (δ = 5.32 ppm), CHD_2CN contained in d_3-MeCN (δ = 1.94 ppm) and $(CHD_2)(CD_3)CO$ contained in $(CD_3)_2CO$ (δ = 2.05 ppm). The ^{13}C NMR spectra are calibrated on the respective resonance signals of CD_2Cl_2 (δ = 53.84 ppm), d_3-MeCN (δ = 1.32 and 118.26 ppm) and $(CD_3)_2CO$ (δ = 29.84 and 206.26 ppm).[1,2] The ^{19}F and ^{31}P NMR spectra are device-internally calibrated relative to the resonance signal of $CFCl_3$ and H_3PO_4 according to the unified chemical shift scale.[3] The given multiplicities are phenomenological, thus the actual appearance of the signals is stated and not the theoretically expected one. The following abbreviations were used and analogously combined to designate multiplicities: s (singlet), d (doublet), t (triplet), q (quartet), m (multiplet), m_c (centrosymmetric multiplet). For centrosymmetric multiplets the center and for non-symmetric multiplets the interval is stated. Evaluation of spectra was performed with Mestrelab Research MNova 7.[4]

Infrared (IR) spectroscopy

IR spectra were measured on a FT (Fourier transformation) Nicolet iS10. The sample was directly measured by ATR (attenuated total reflection) technique. Characteristic absorptions are given in wavenumbers $\tilde{\nu}$ [cm^{-1}] and intensities are stated as vs (very strong), s (strong), m (medium) and w (weak).

High resolution mass spectrometry (HRMS) and elemental analysis (EA)

HRMS was recorded using an AGILENT 6210 spectrometer by electrospray ionization (ESI) or a VARIAN MAT 711 by electron impact ionization (EI) at the department of mass spectrometry at the Freie Universität Berlin. A detailed listing of fragmentation is dispensed, instead the molecular ion peak or a characteristic fragment peak is stated. EA was measured on a VARIO EL. Relative proportion of C, H and N are given in percent.

Ultraviolet/visible light (UV/VIS) spectroscopy

UV/VIS spectra were recorded on a PerkinElmer Lambda 465 photometer with deuterium and tungsten lamps. A fiber optic probe was used to record the spectra.

X-ray diffraction (XRD)

X-Ray data were collected on a BRUKER D8 Venture system. Data were collected at 100(2) K using graphite monochromated Mo K_α radiation (λ_α = 0.71073 Å). The strategy for the data collection was evaluated by using the Smart software. The data were collected by the standard "ψ-ω scan techniques" and were scaled and reduced using Saint+software. The structures were solved by using Olex2,[5] the structure was solved with the XT[6] structure solution program using Intrinsic Phasing and refined with the XL refinement package[7,8] using the Least Squares minimization. Bond length and angles were measured with Diamond Crystal and Molecular Structure Visualization Version 4.6.2.[9] Drawings were generated with POV-Ray.[10]

Density functional theory (DFT) calculations

DFT calculations were performed with Gaussian 16 (Revision C.02).[11] Structure optimizations and population analysis (gas phase) were done using B3LYP-D3BJ/def2TZVP. Solutions were processed using Avogadro[12] and Chemcraft 1.8.[13]

Synthetic Procedures

[NEt_4][$C_5(CF_3)_5$]

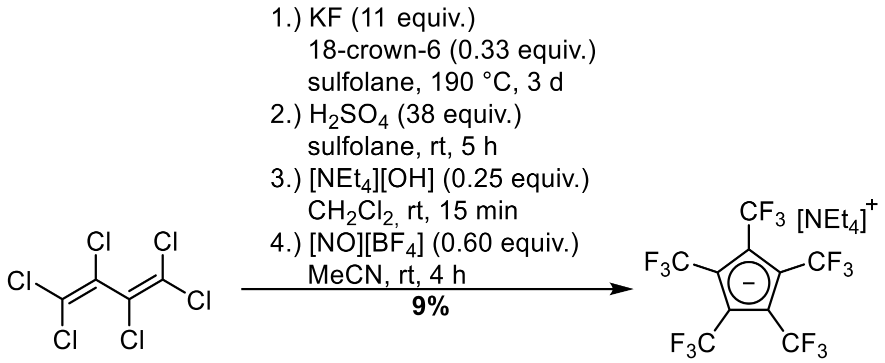

In a dried 1000 mL pressure flask anhydrous KF (60 g, 1.0 mol, 11 equiv.) was placed in anhydrous and degassed sulfolane (190 mL) under an atmosphere of argon. Anhydrous and degassed 18-crown-6 (8.7 g, 33 mmol, 0.33 equiv.) and hexachlorobuta-1,3-diene (15 mL, 96 mmol, 1.0 equiv.) were added at rt. The resulting reaction mixture was carefully shaken and cooled to –196 °C in high vacuum. The properly closed pressure flask was slowly warmed to 190 °C and stirred at this temperature for 3 d. Then the resulting black suspension was cooled to rt and the volatiles were removed in high vacuum. The remaining mixture was filtrated under an atmosphere of argon and the residue was extracted with anhydrous MeCN (3 × 40 mL). The filtrate was warmed to 40 °C and all MeCN was removed in high vacuum, while stirring. The resulting solution was put under high vacuum and H_2SO_4 (conc., 200 mL, 3.6 mol, 38 equiv.) was added dropwise at rt over a period of 3 h, while stirring and continuously collecting the volatiles in a cold trap of –196 °C. After complete addition, the mixture remained for additional 2 h in high vacuum. The cold trap was put under argon and slowly warmed to 0 °C, giving a pale yellow liquid. Then CH_2Cl_2 (20 mL) and a solution of [NEt_4][OH] (35% aqueous, 10 mL, 24 mmol, 0.25 equiv.) were added and the reaction mixture was stirred for 15 min at rt, giving a deep red solution. The aqueous layer was separated and extracted with CH_2Cl_2 (4 × 20 mL). The combined organic layers were dried over $MgSO_4$, filtrated and the solvent was removed under reduced pressure. The remaining solid was suspended in Et_2O (~5 mL) and recrystallized twice from CH_2Cl_2 (~10 mL) by slowly cooling to –20 °C. The crystalline residue was decanted and washed with Et_2O (2 × 5 mL). The solvents were removed under reduced pressure to give a product mixture of 85 mol% [NEt_4][$C_5(CF_3)_5$] (1.7 g, 3.2 mmol) and 15 mol% [NEt_4][$C_5(CF_3)_4H$] (0.30 g, 0.50 mmol) that was placed in a dried 50 mL Schlenk flask in anhydrous MeCN (20 mL). [NO][BF_4] (0.26 g, 2.2 mmol, 0.60 equiv.) was added and the reaction mixture was stirred at room temperature for 4 h. Dest. H_2O (20 mL) was slowly added and stirred for 15 min before separating aqueous and organic phase. The aqueous phase was extracted with CH_2Cl_2 (3 × 20 mL). The combined organic phases were dried over $MgSO_4$, filtrated and the solvents removed under reduced pressure. The residue was dissolved in CH_2Cl_2 (1 mL) and slowly added to stirred *n*Bu_2O (200 mL). The colorless suspension was filtrated and the residue was washed with *n*Bu_2O (2 × 10 mL) and n-pentane (2 × 10 mL). The solvent was removed under reduced pressure to give [NEt_4][$C_5(CF_3)_5$] (1.7 g, 3.2 mmol) as a colorless amorphous solid with a yield of 9%.

^{1}H NMR (400 MHz, CD_2Cl_2, rt) δ [ppm] = 2.95 (q, $^3J_{H,H}$ = 7.3 Hz, 8H), 1.19 (q, $^3J_{H,H}$ = 7.1 Hz, 12H). **^{19}F NMR** (377 MHz, CD_2Cl_2, rt) δ [ppm] = –50.6 (s, 15F). **^{13}C{^{1}H} NMR** (151 MHz, CD_2Cl_2, rt) δ [ppm] = 52.7 (m_c, 4C), 7.3 (s, 4C). **^{13}C{^{19}F} NMR** (151 MHz, CD_2Cl_2, rt) δ [ppm] = 124.9 (s, 5C), 109.6 (s, 5C). The analytical data are consistent with those reported in literature.[14–16]

[Fe(C_5H_5)(*o*DCB)][PF_6]

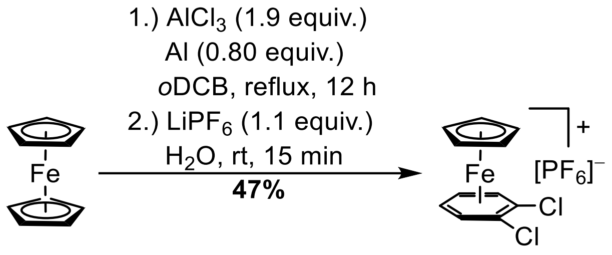

In a dried 500 mL Schlenk flask [Fe(C_5H_5)$_2$] (6.0 g, 32 mmol, 1.0 equiv.), powdered aluminum (0.72 g, 27 mmol, 0.80 equiv.) and $AlCl_3$ (8.0 g, 60 mmol, 1.9 equiv.) were suspended in anhydrous *o*DCB (100 mL) under an atmosphere of argon. The reaction mixture was stirred under reflux for 12 h. The brownish suspension was cooled to 0 °C and dest. H_2O (300 mL) was slowly added while stirring. The aqueous phase was isolated and washed with *n*-pentane (3 × 100 mL). A solution of $LiPF_6$ (5.2 g, 36 mmol, 1.1 equiv.) in dest. H_2O (40 mL) was added and the reaction mixture was stirred for 15 min. The resulting suspension was filtrated and the residue was washed with dest. H_2O (3 × 40 mL). The crude solid was dissolved in acetone (5 mL) and slowly added to stirred Et_2O (100 mL). The suspension was filtrated and the residue was washed with Et_2O (3 × 40 mL) and dried under reduced pressure to give [Fe(C_5H_5)(*o*DCB)][PF_6] (6.2 g, 15 mmol) as a greenish amorphous solid with a yield of 47%.

^{1}H NMR (400 MHz, $(CD_3)_2CO$, rt) δ [ppm] = 7.07 (s, 2H), 6.61 (s, 2H), 5.39 (s, 5H). **^{19}F NMR** (377 MHz, $(CD_3)_2CO$, rt) δ [ppm] = –72.2 (d, $^1J_{F,P}$ = 708.1 Hz, 6F). **^{31}P{^{19}F} NMR** (162 MHz, $(CD_3)_2CO$, rt) δ [ppm] = –144.2 (s, 1P). **^{13}C{^{1}H} NMR** (100 MHz, $(CD_3)_2CO$, rt) δ [ppm] = 107.5 (s, 2C), 89.4 (s, 2C), 88.3 (s, 2C), 81.7 (s, 5C). The analytical data are consistent with those reported in literature.[17]

[Fe(C_5H_5)($C_5(CF_3)_5$)]

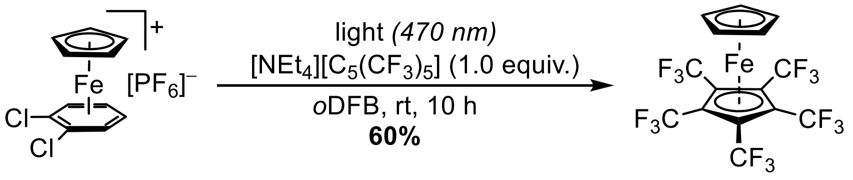

In a dried 10 mL Schlenk flask [Fe(C_5H_5)(*o*DCB)][PF_6] (0.17 g, 0.40 mmol, 1.0 equiv.) and [NEt_4][$C_5(CF_3)_5$] (0.21 g, 0.40 mmol, 1.0 equiv.) were dissolved in anhydrous *o*DFB (3 mL). The reaction mixture was stirred for 10 h at room temperature under visible light (470 nm) irradiation. Silica gel (200 mg) was added and the solvent was removed in high vacuum. The crude product loaded on silica was purified by column chromatography with *n*-pentane (first fraction). The organic fractions were combined and the solvent was removed under reduced pressure and the product was recrystallized from perfluorohexanes. The product [Fe(C_5H_5)($C_5(CF_3)_5$)] (0.12 g, 0.24 mmol) was obtained as a greenish crystalline solid with a yield of 60%.

^{1}H NMR (400 MHz, CD_2Cl_2, rt) δ [ppm] = 4.94 (s, 5H). **^{19}F NMR** (377 MHz, CD_2Cl_2, rt) δ [ppm] = –50.3 (s, 15F). **^{13}C{^{1}H} NMR** (151 MHz, CD_2Cl_2, rt) δ [ppm] = 78.1 (s, 5C). **^{13}C{^{19}F} NMR** (151 MHz, CD_2Cl_2, rt) δ [ppm] = 123.3 (m_c, 5C), 112.5 (s, 5C). The analytical data are consistent with those reported in literature.[18]

Substitution experiment of [Fe(C_5H_5)($C_5(CF_3)_5$)]:

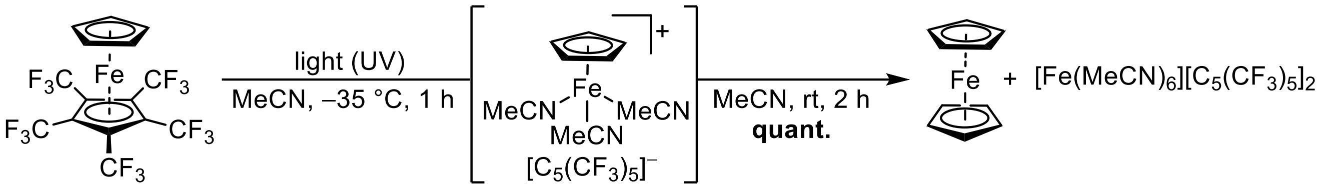

In a 10 mL Schlenk flask [Fe(C_5H_5)($C_5(CF_3)_5$)] (30 mg, 70 µmol, 1.0 equiv.) was dissolved in anhydrous and degassed d_3-MeCN or MeCN (1 mL). The greenish reaction mixture was stirred for 1 h at –35 °C under UV irradiation, giving a deep purple solution of [Fe(C_5H_5)(MeCN)$_3$][$C_5(CF_3)_5$] with a conversion of 95% according to NMR spectroscopy (see Figure S17 and S18 for reaction screening experiment).

[Fe(C_5H_5)(d_3-MeCN)$_3$][$C_5(CF_3)_5$]: **^{1}H NMR** (400 MHz, d_3-MeCN, –35 °C) δ [ppm] = 3.93 (s, 5H). **^{19}F NMR** (377 MHz, d_3-MeCN, –35 °C) δ [ppm] = –51.0 (s, 15F).

The reaction mixture was warmed to room temperature and stirred for another 2 h. The solvent of the yellow solution was removed in high vacuum and the products were separated by extraction with *n*-pentane (3 × 5 mL). The solvent was removed in high vacuum giving products [Fe(C_5H_5)$_2$] (from *n*-pentane solution) as an orange amorphous solid and [Fe(MeCN)$_6$][$C_5(CF_3)_5$]$_2$ (from residue) as an colorless amorphous solid with a quantitative yield.

[Fe(MeCN)$_6$][$C_5(CF_3)_5$]: **^{1}H NMR** (400 MHz, d_3-MeCN, rt) δ [ppm] = 1.96 (s, 18H). **^{19}F NMR** (377 MHz, d_3-MeCN, rt) δ [ppm] = –51.0 (s, 15F). The analytical data are consistent with those reported in literature.[15]

[Fe(C_5H_5)(DPPE)(MeCN)][$C_5(CF_3)_5$]:

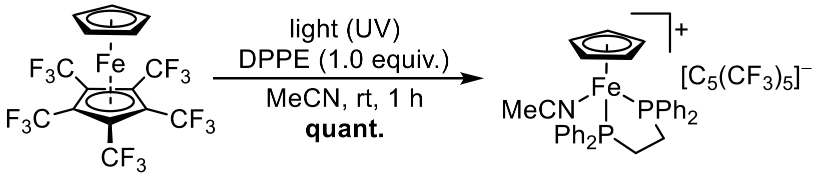

In a 10 mL Schlenk flask [Fe(C_5H_5)($C_5(CF_3)_5$)] (15 mg, 29 µmol, 1.0 equiv.) and DPPE (12 mg, 29 µmol, 1.0 equiv.) were dissolved in anhydrous and degassed MeCN (2 mL). The greenish reaction mixture was stirred for 1 h at room temperature under UV irradiation. The solvent of the deep red solution was removed in high vacuum and the residue washed with anhydrous *n*-pentane (3 × 5 mL). The solvent was removed in high vacuum and the product [Fe(C_5H_5)(DPPE)(MeCN)][$C_5(CF_3)_5$] (28 mg, 29 µmol) was obtained as a red amorphous solid with a quantitative yield.

^{1}**H NMR** (400 MHz, CD_2Cl_2, rt) *δ* [ppm] = 7.73 (m_c, 4H), 7.57–7.52 (m, 6H), 7.52–7.44 (m, 6H), 7.34–7.27 (m, 4H), 4.30 (s, 5H), 2.60–2.48 (m, 2H), 2.40–2.23 (m, 2H), 1.35 (s, 3H). ^{19}**F NMR** (377 MHz, CD_2Cl_2, rt) *δ* [ppm] = –50.6 (s, 15F). ^{31}**P{^{1}H} NMR** (162 MHz, CD_2Cl_2, rt) *δ* [ppm] = –97.4 (s, 2P). ^{13}**C{^{1}H} NMR** (100 MHz, CD_2Cl_2, rt) *δ* [ppm] = 136.8 (m_c, 4C), 133.6 (s, 1C), 132.8 (t, $^{2}J_{C,P}$ = 4.7 Hz, 4C), 131.6 (t, $^{2}J_{C,P}$ = 4.7 Hz, 4C), 131.3 (d, $^{1}J_{C,P}$ = 31.0 Hz, 4C), 129.5 (m_c, 8C), 78.2 (s, 5C), 28.3 (t, $^{1}J_{C,P}$ = 21.0 Hz, 2C), 4.0 (s, 1C). ^{13}**C{^{19}F} NMR** (100 MHz, CD_2Cl_2, rt) *δ* [ppm] = 124.1 (s, 5C), 110.0 (s, 5C). **FT-IR** (ATR) ṽ [cm^{-1}] = 3064 (w), 2273 (w), 1493 (m), 1435 (m), 1210 (vs), 1122 (vs), 1026 (m), 1000 (m), 917 (w), 876 (m), 847 (m), 811 (m), 744 (s), 693 (vs), 633 (s), 594 (w), 565 (w), 533 (vs). **HRMS** (ESI TOF, positive) m/z for $[FeC_{31}H_{30}P_2]^+$ calculated: 520.1172; measured: 520.1165. **HRMS** (ESI TOF, negative) m/z for $[C_{10}F_{15}]^-$ calculated: 404.9760; measured: 404.9803. **EA** $[FeC_{43}H_{32}NP_2F_{15}]$ calculated: C: 53.49%, H: 3.34%, N: 1.45; measured: C: 53.85%, H: 3.37%, N: 1.59%. A molecular structure in the solid state was determined for this compound (see Table S1 and Figure S39).

$[Ru(C_5H_5)(C_5(CF_3)_5)]$:

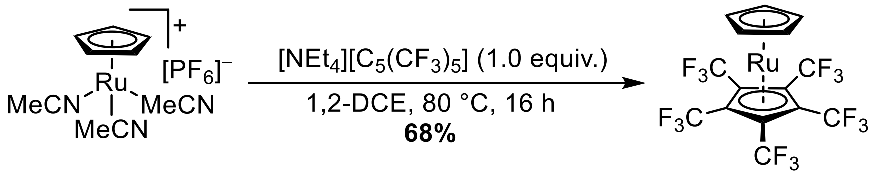

In a dried 10 mL Schlenk flask $[Ru(C_5H_5)(MeCN)_3][PF_6]$ (0.15 g, 0.35 mmol, 1.0 equiv.) and $[NEt_4][C_5(CF_3)_5]$ (0.19 g, 0.35 mmol, 1.0 equiv.) were dissolved in anhydrous 1,2-DCE (3 mL). The reaction mixture was stirred for 16 h at 80 °C. The solvent was removed in high vacuum and the residue was extracted with *n*-pentane (3 × 5 mL). The organic fractions were combined and the solvent was removed in high vacuum. The residue was recrystallized from perfluorohexanes and the product $[Ru(C_5H_5)(C_5(CF_3)_5)]$ (0.14 g, 0.24 mmol) was obtained as a colorless crystalline solid with a yield of 68%.

^{1}**H NMR** (400 MHz, CD_2Cl_2, rt) *δ* [ppm] = 5.24 (s, 5H). ^{19}**F NMR** (377 MHz, CD_2Cl_2, rt) *δ* [ppm] = –50.6 (s, 15F). ^{13}**C{^{1}H} NMR** (101 MHz, CD_2Cl_2, rt) *δ* [ppm] = 81.0 (s, 5C). ^{13}**C{^{19}F} NMR** (101 MHz, CD_2Cl_2, rt) *δ* [ppm] = 122.9 (s, 5C).[19] **FT-IR** (ATR) ṽ [cm^{-1}] = 3120 (w), 2925 (w), 1427 (s), 1160 (vs), 1141 (vs), 1005 (m), 984 (m), 868 (w), 846 (s), 808 (m), 708 (w), 656 (vs), 630 (s), 527 (w). **HRMS** (EI TOF, positive) m/z for $[RuC_{15}H_5F_{15}]^+$ calculated: 571.9195; measured: 571.9194. **EA** $[RuC_{15}H_5F_{15}]$ calculated: C: 31.54%, H: 0.88%; measured: C: 30.73%, H: 0.99%. A molecular structure in solid state was measured for this compound (see Table S2 and Figure S40).

Substitution experiment of $[Ru(C_5H_5)(C_5(CF_3)_5)]$:

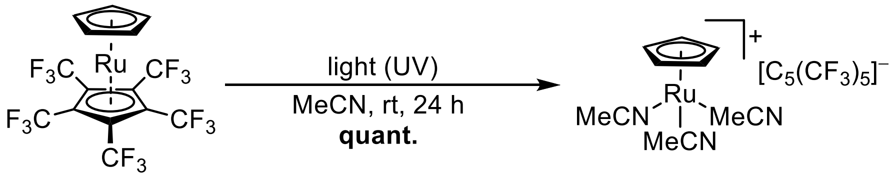

In a 10 mL Schlenk flask [Ru(C_5H_5)($C_5(CF_3)_5$)] (30 mg, 53 µmol, 1.0 equiv.) was dissolved in anhydrous MeCN (2 mL). The greenish reaction mixture was stirred for 24 h at room temperature under UV irradiation. The solvent of the yellow solution was removed in high vacuum and the residue was washed with *n*-pentane (3 × 5 mL). The product [Ru(C_5H_5)(MeCN)$_3$][$C_5(CF_3)_5$] (37 mg, 53 µmol) was obtained as a yellow amorphous solid in quantitative yield (see Figure S32 and S33 for reaction screening experiment).

^{1}H NMR (400 MHz, CD_2Cl_2, rt) δ [ppm] = 4.24 (s, 5H), 2.29 (s, 9H). **^{19}F NMR** (377 MHz, CD_2Cl_2, rt) δ [ppm] = –50.6 (s, 15F). **^{13}C{^{1}H} NMR** (100 MHz, CD_2Cl_2, rt) δ [ppm] = 69.5 (s, 5C), 4.0 (s, 3C).[20] **^{13}C{^{19}F} NMR** (100 MHz, CD_2Cl_2, rt) δ [ppm] = 124.1 (s, 5C), 110.0 (s, 5C). **FT-IR** (ATR) ṽ [cm^{-1}] = 2942 (w), 2289 (w), 1550 (w), 1493 (m), 1414 (w), 1296 (w), 1203 (vs), 1112 (vs), 1034 (m), 993 (m), 947 (w), 863 (w), 801 (w), 725 (w), 633 (s). **HRMS** (ESI TOF, positive) m/z for [$RuC_{11}H_{14}N_3$]$^+$ calculated: 290.0231; measured: 290.0199. **HRMS** (ESI TOF, negative) m/z for [$C_{10}F_{15}$]$^-$ calculated: 404.9760; measured: 404.9747. **EA** [$RuC_{21}H_{14}N_3F_{15}$] calculated: C: 36.32%, H: 2.03%, N: 6.05%; measured: C: 35.79%, H: 1.77%, N: 5.51%. A molecular structure in the solid state was determined for this compound (see Table S3 and Figure S41).

Substitution experiment of [Ru(C_5H_5)(MeCN)$_3$][$C_5(CF_3)_5$]:

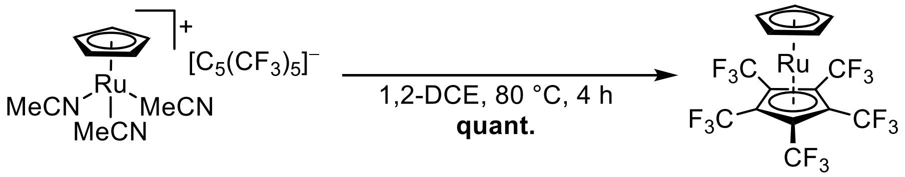

In a 10 mL Schlenk flask [Ru(C_5H_5)(MeCN)$_3$][$C_5(CF_3)_5$] (25 mg, 36 µmol, 1.0 equiv.) was dissolved in anhydrous 1,2-DCE (2 mL). The yellow reaction mixture was stirred for 4 h at 80 °C. The solvent of the colorless solution was removed in high vacuum. The product [Ru(C_5H_5)($C_5(CF_3)_5$)] (21 mg, 36 µmol) was obtained as a colorless amorphous solid with a quantitative yield (see Figure S34 for reaction screening experiment).

NMR Spectra

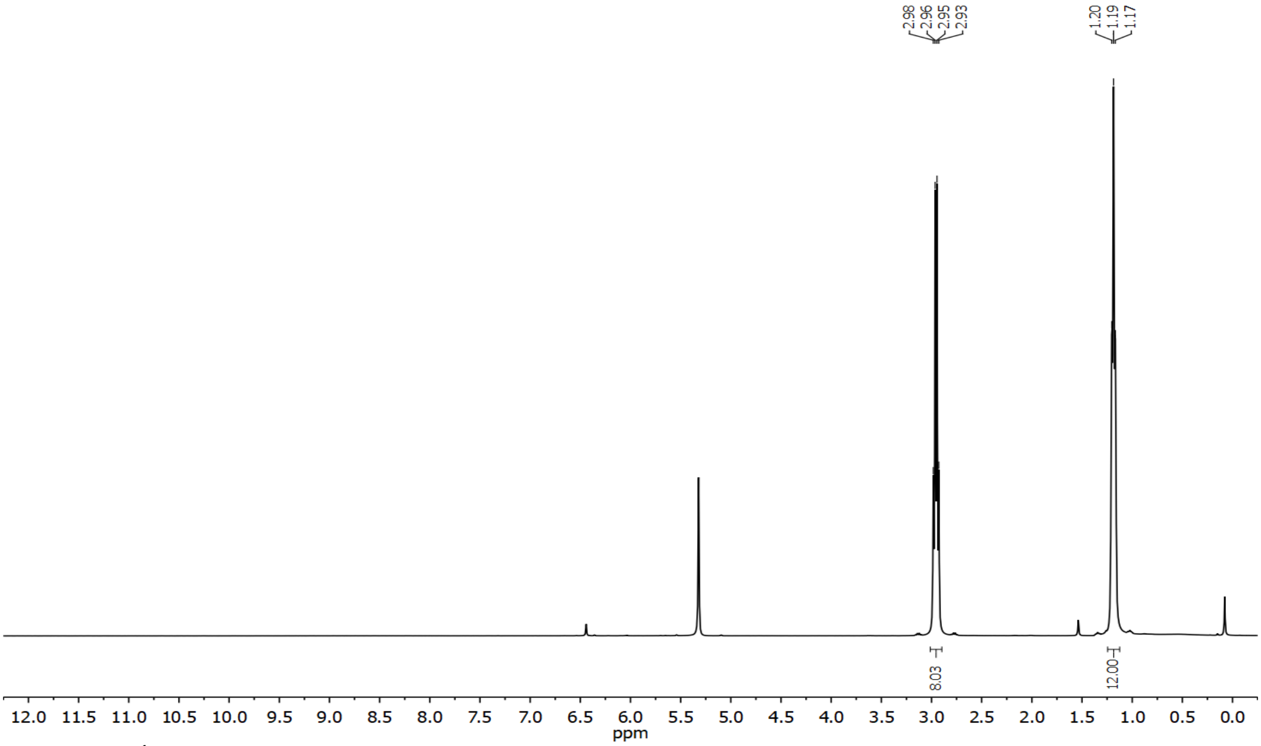

Figure S1. ^{1}H NMR (400 MHz, CD_2Cl_2, rt) spectrum of $[NEt_4][C_5(CF_3)_5]$.

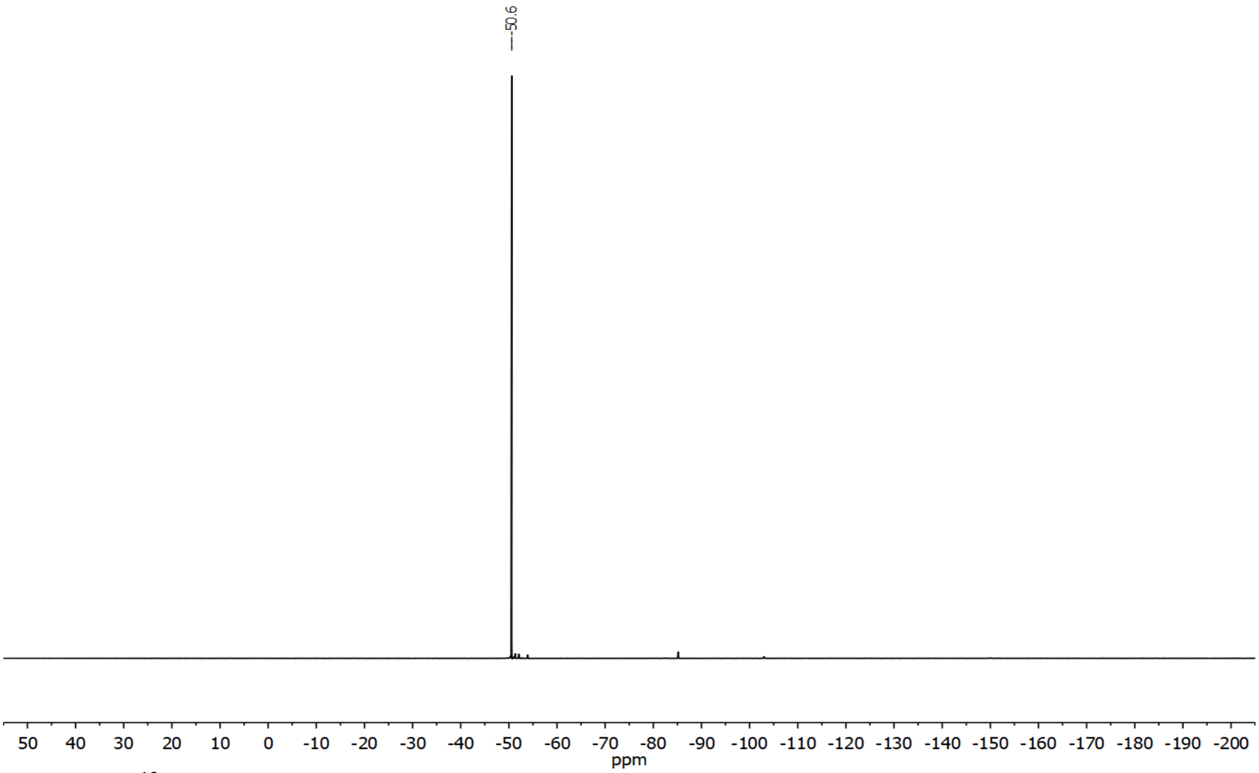

Figure S2. ^{19}F NMR (377 MHz, CD_2Cl_2, rt) spectrum of $[NEt_4][C_5(CF_3)_5]$.

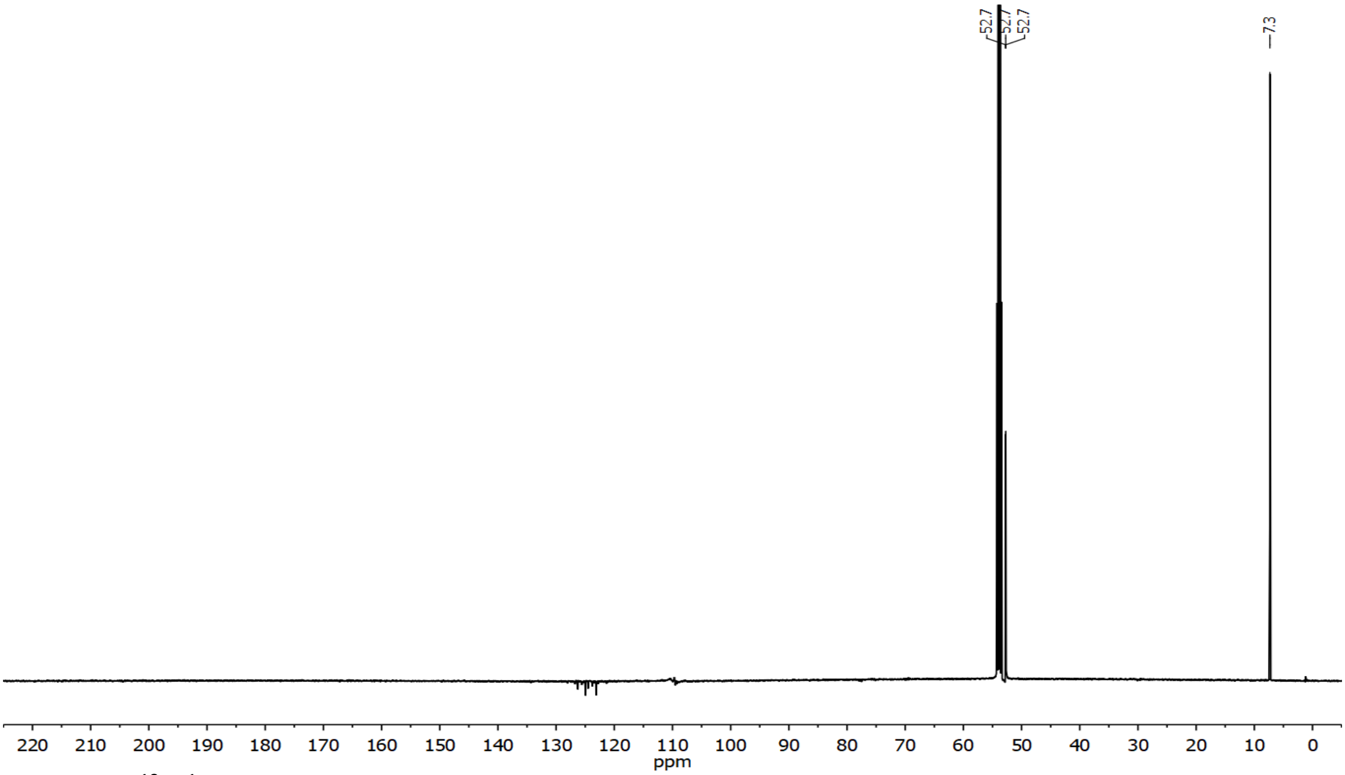

Figure S3. $^{13}C\{^1H\}$ NMR (151 MHz CD_2Cl_2, rt) spectrum of $[NEt_4][C_5(CF_3)_5]$.

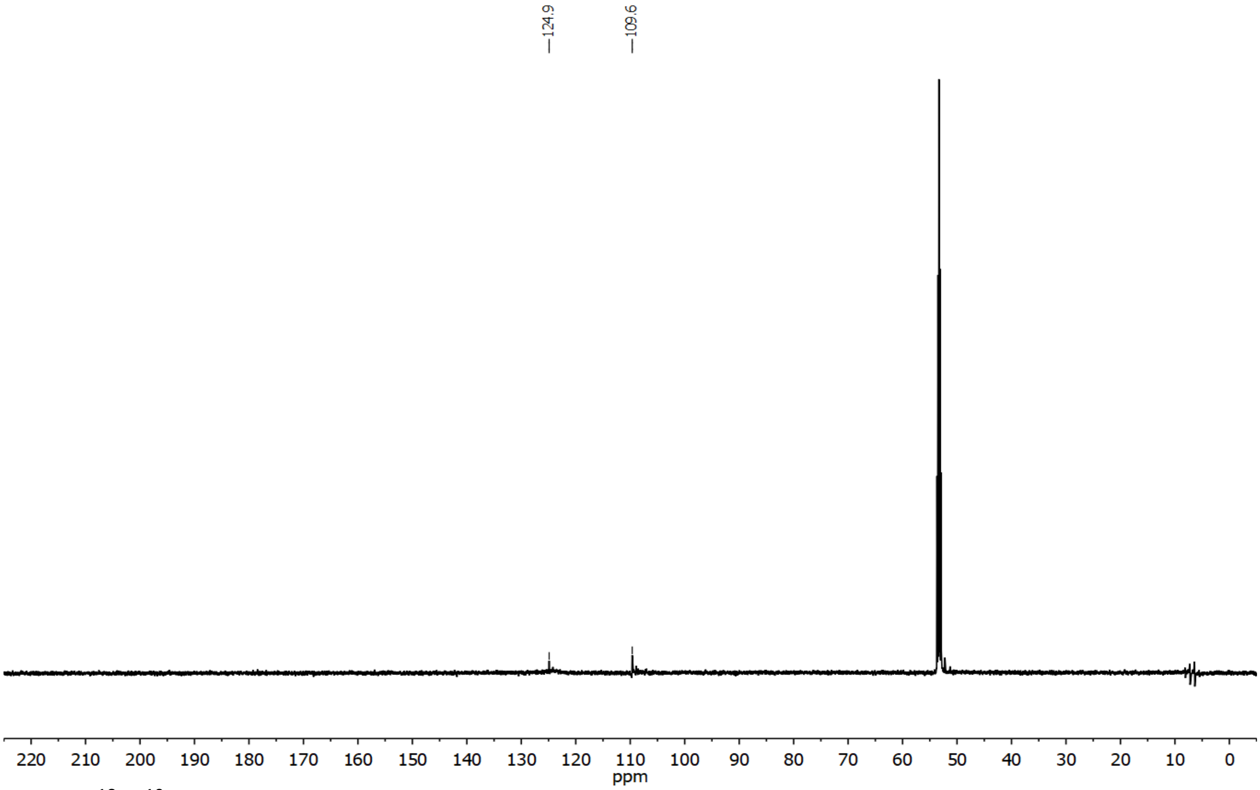

Figure S4. $^{13}C\{^{19}F\}$ NMR (151 MHz CD_2Cl_2, rt) spectrum of $[NEt_4][C_5(CF_3)_5]$.

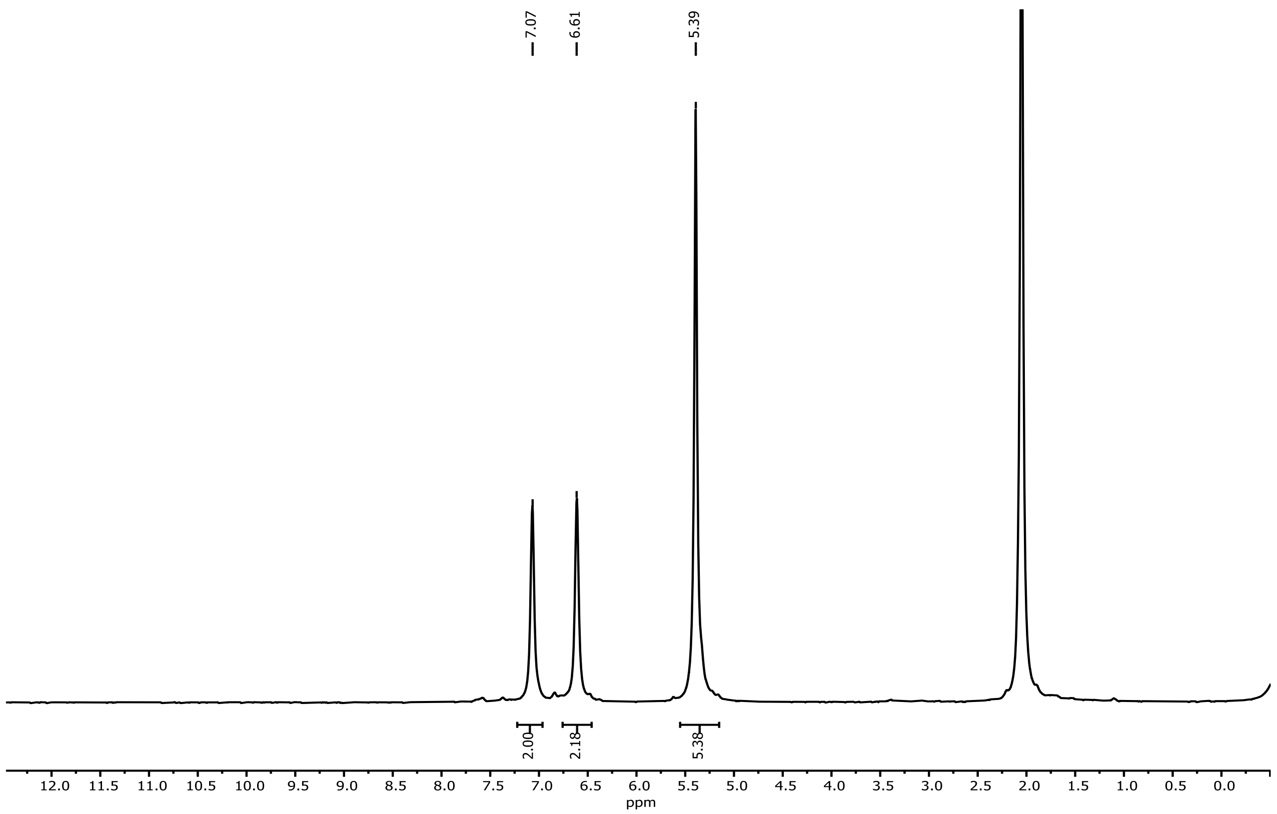

Figure S5. ^{1}H NMR (400 MHz, $(CD_3)_2CO$, rt) spectrum of $[Fe(C_5H_5)(o\text{DCB})][PF_6]$.

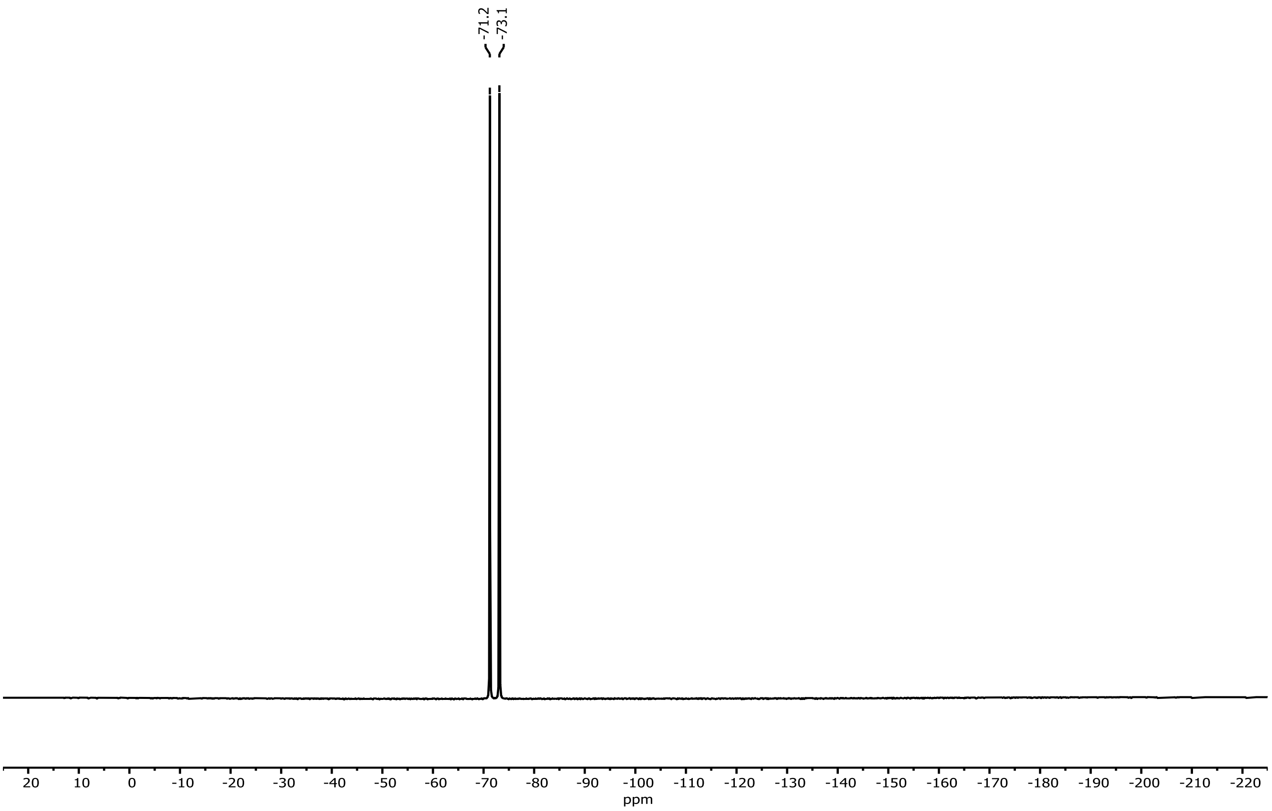

Figure S6. ^{19}F NMR (377 MHz, $(CD_3)_2CO$, rt) spectrum of $[Fe(C_5H_5)(o\text{DCB})][PF_6]$.

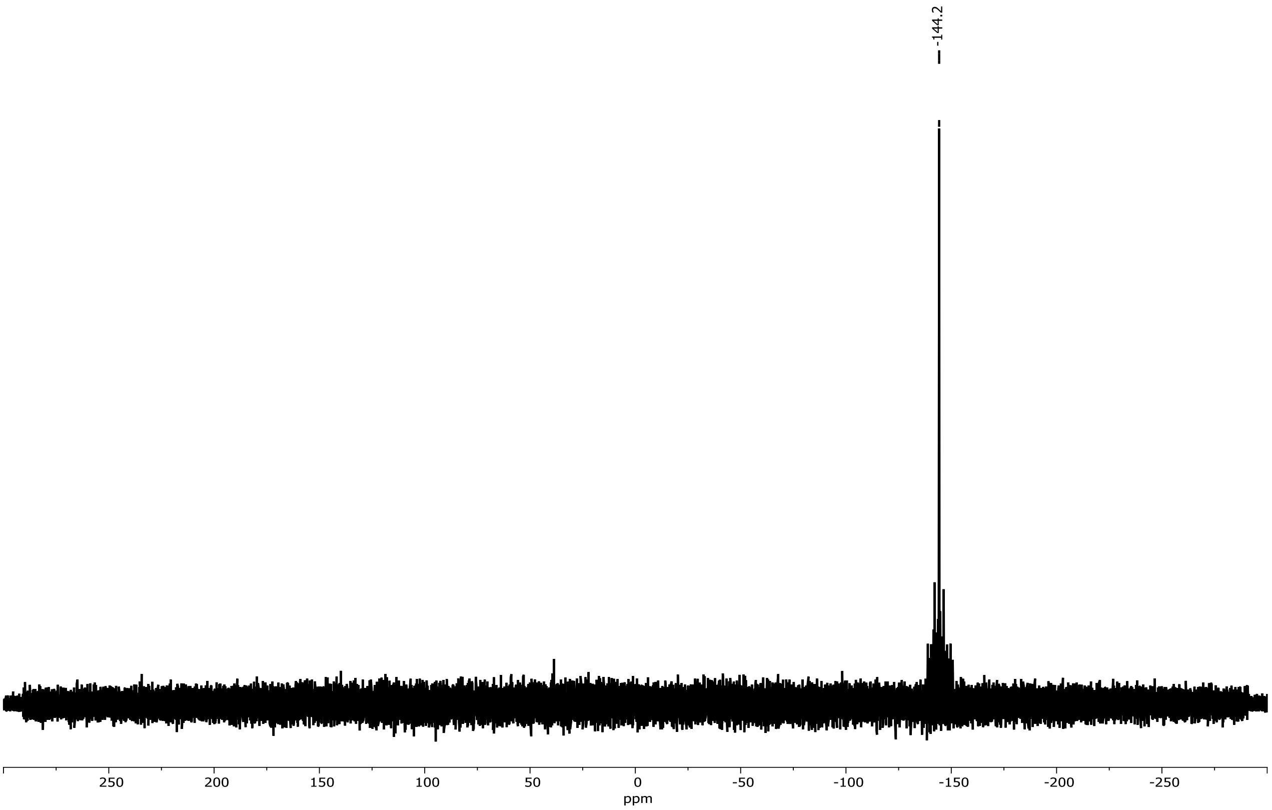

Figure S7. $^{31}P\{^{19}F\}$ NMR (162 MHz, $(CD_3)_2CO$, rt) spectrum of $[Fe(C_5H_5)(o\text{DCB})][PF_6]$.

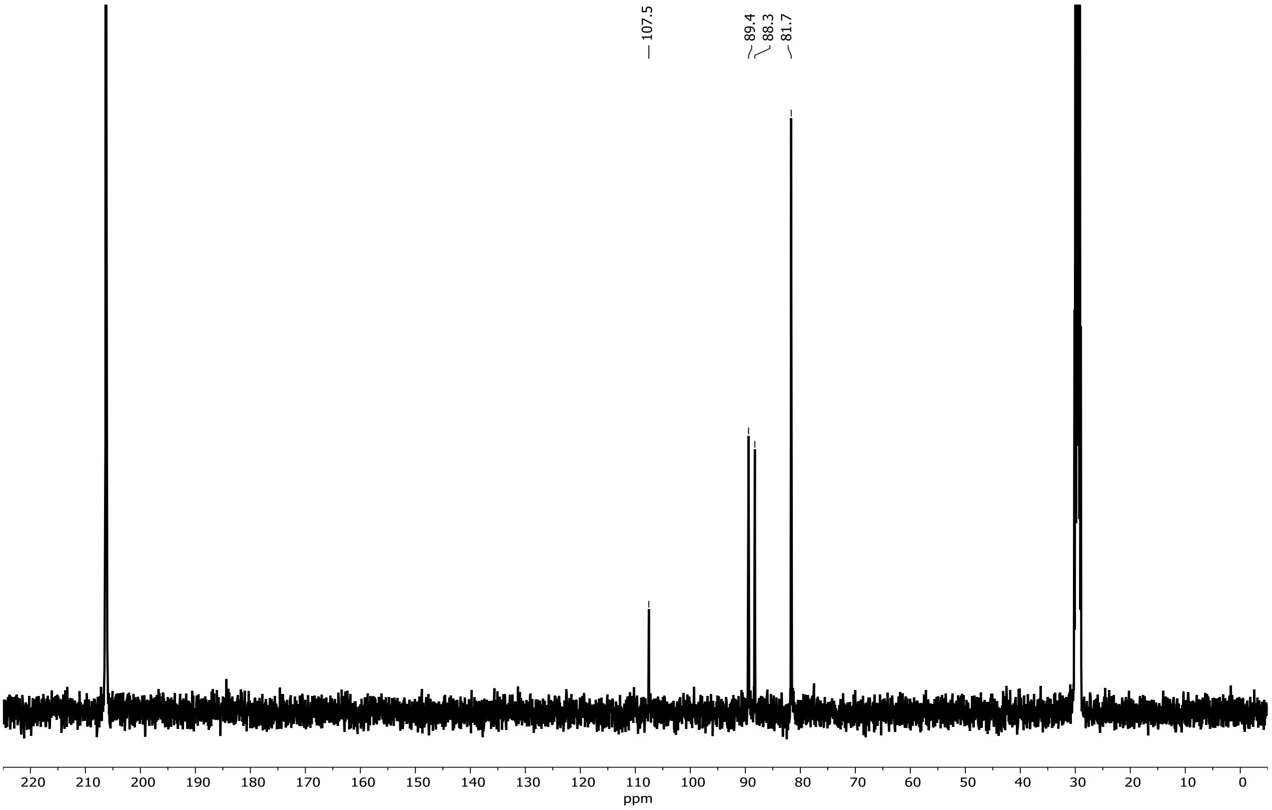

Figure S8. $^{13}C\{^{1}H\}$ NMR (100 MHz $(CD_3)_2CO$, rt) spectrum of $[Fe(C_5H_5)(o\text{DCB})][PF_6]$.

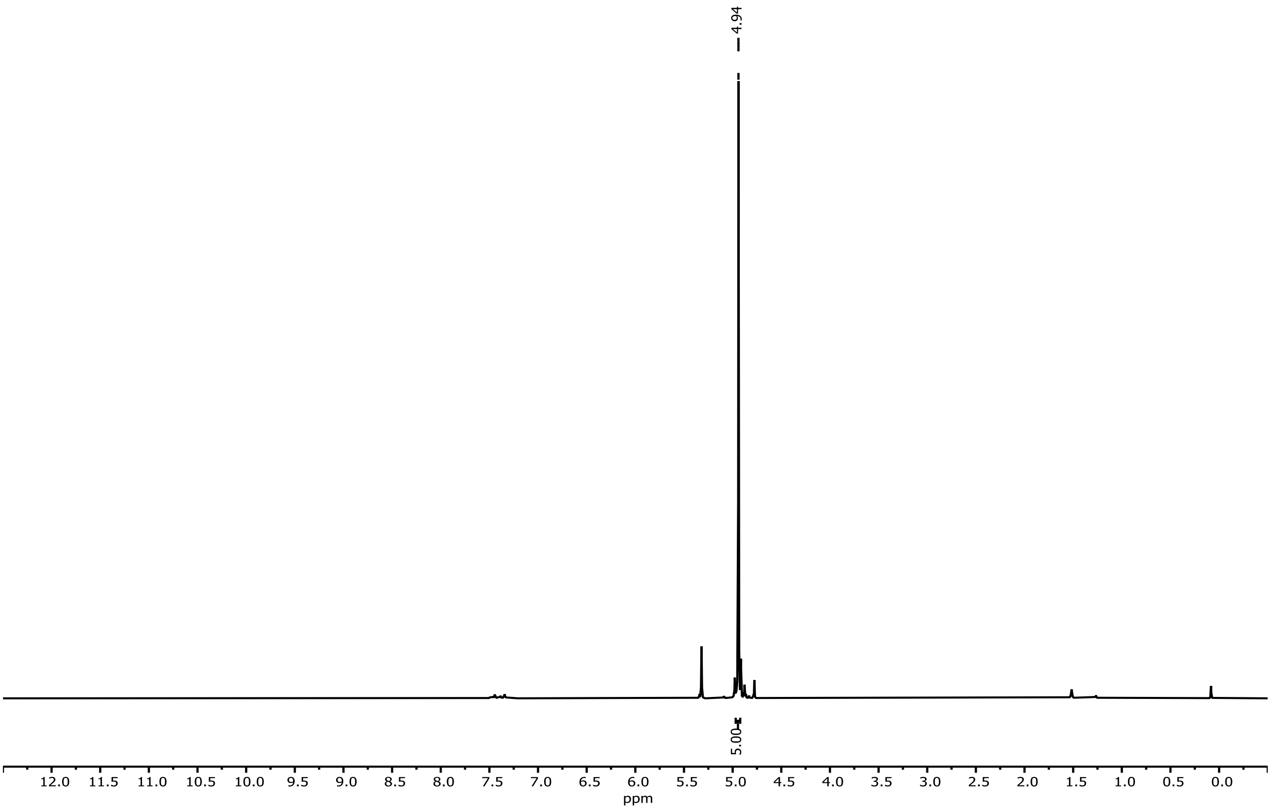

Figure S9. ^{1}H NMR (600 MHz, CD_2Cl_2, rt) spectrum of $[Fe(C_5H_5)(C_5(CF_3)_5)]$.

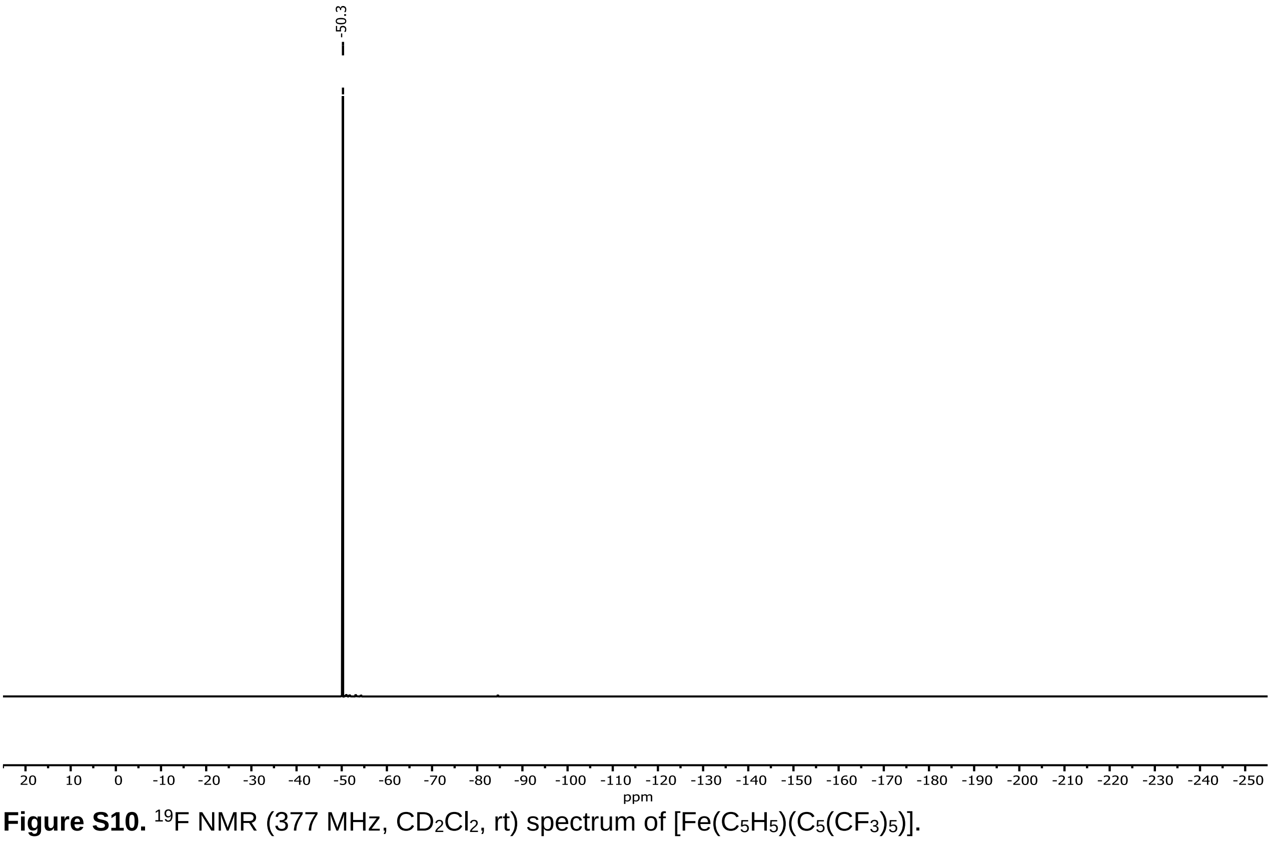

Figure S10. ^{19}F NMR (377 MHz, CD_2Cl_2, rt) spectrum of $[Fe(C_5H_5)(C_5(CF_3)_5)]$.

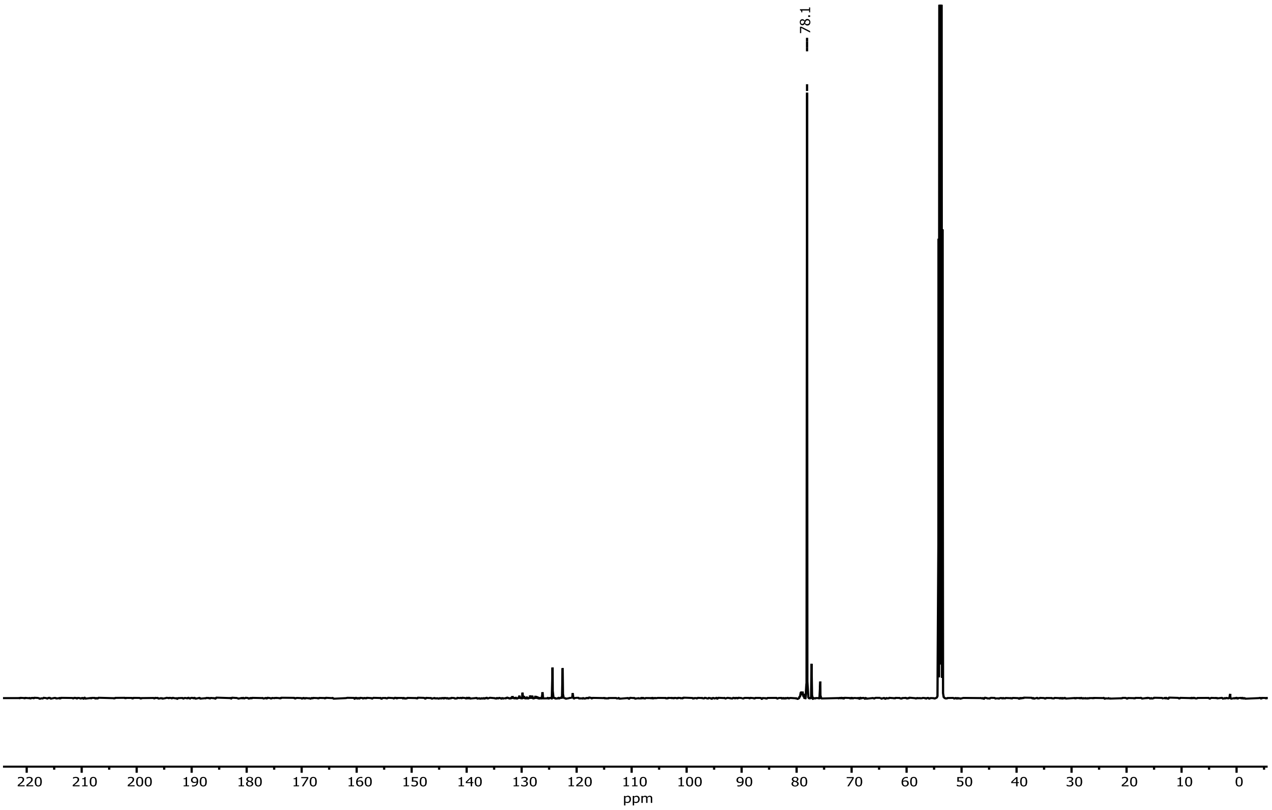

Figure S11. $^{13}C\{^{1}H\}$ NMR (151 MHz, CD_2Cl_2, rt) spectrum of $[Fe(C_5H_5)(C_5(CF_3)_5)]$.

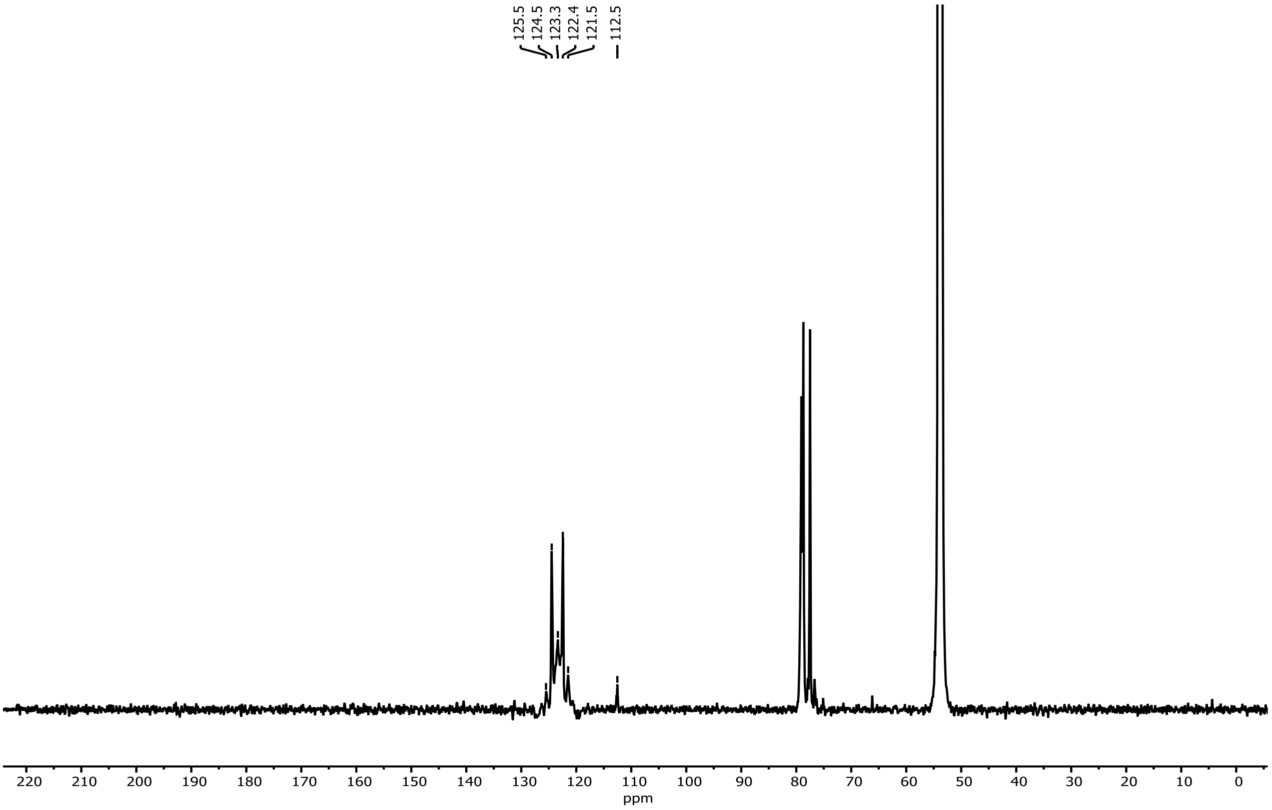

Figure S12. $^{13}C\{^{19}F\}$ NMR (151 MHz, CD_2Cl_2, rt) spectrum of $[Fe(C_5H_5)(C_5(CF_3)_5)]$.

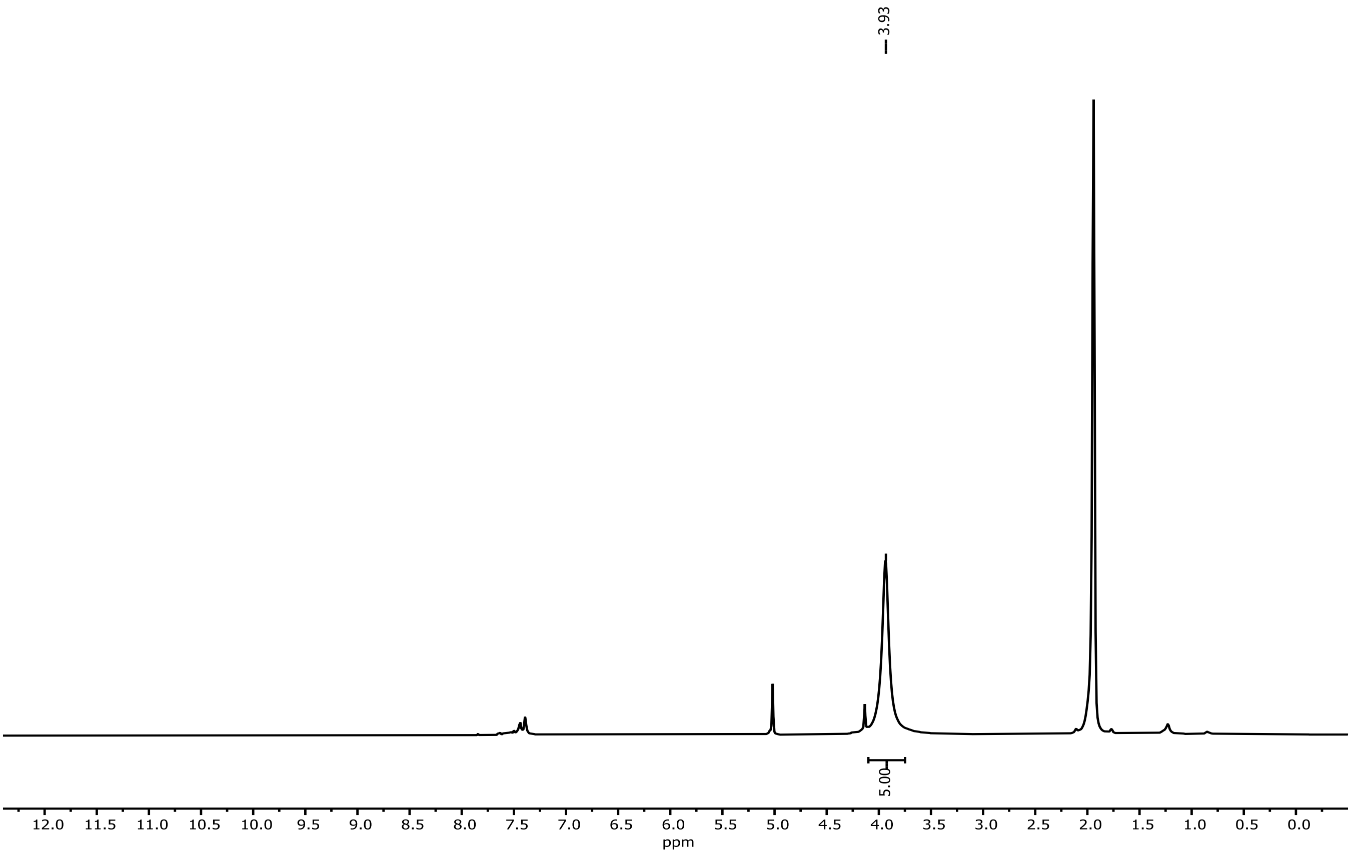

Figure S13. ^{1}H NMR (400 MHz, d_3-MeCN, –35 °C) spectrum of $[Fe(C_5H_5)(d_3\text{-}MeCN)_3][C_5(CF_3)_5]$.

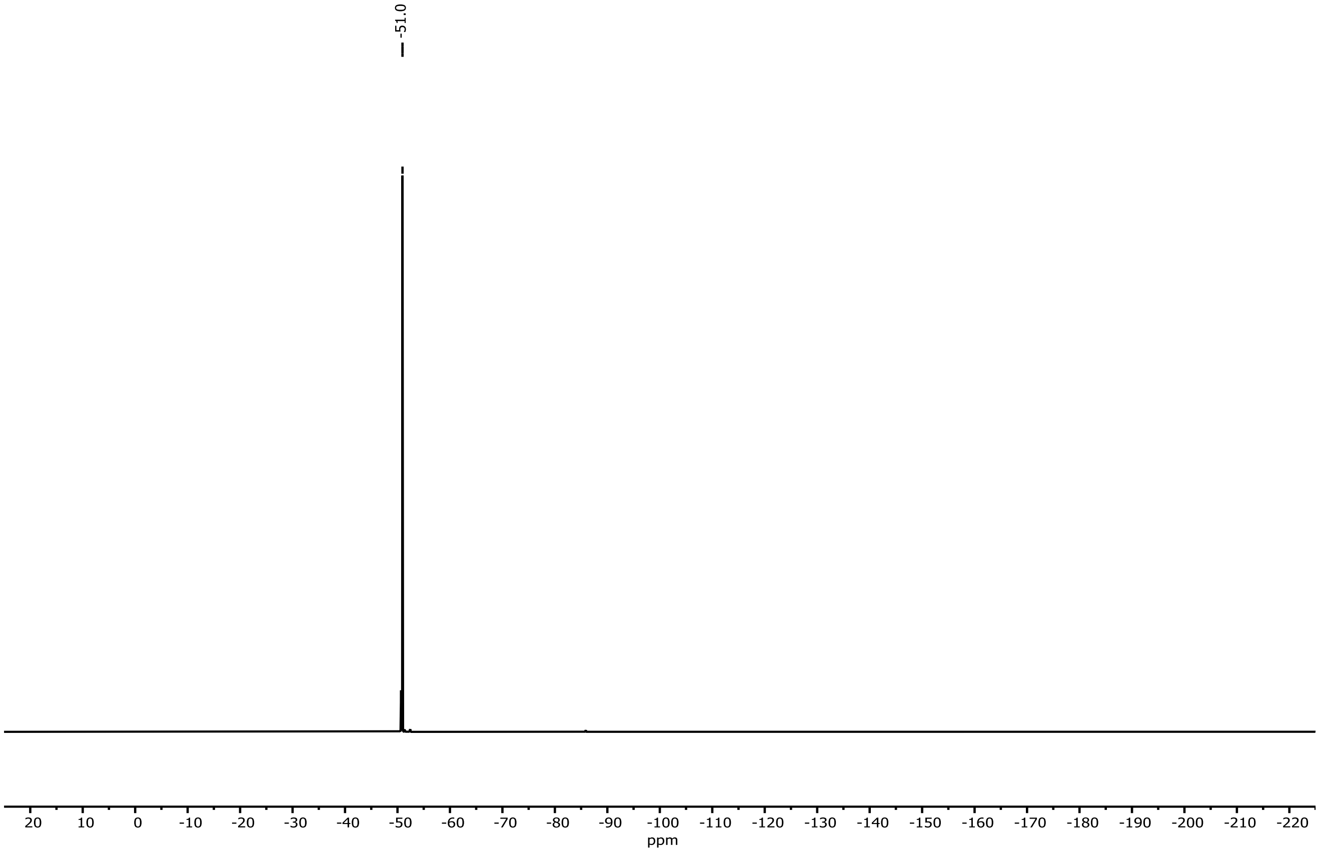

Figure S14. ^{19}F NMR (400 MHz, d_3-MeCN, –35 °C) spectrum of $[Fe(C_5H_5)(d_3\text{-}MeCN)_3][C_5(CF_3)_5]$.

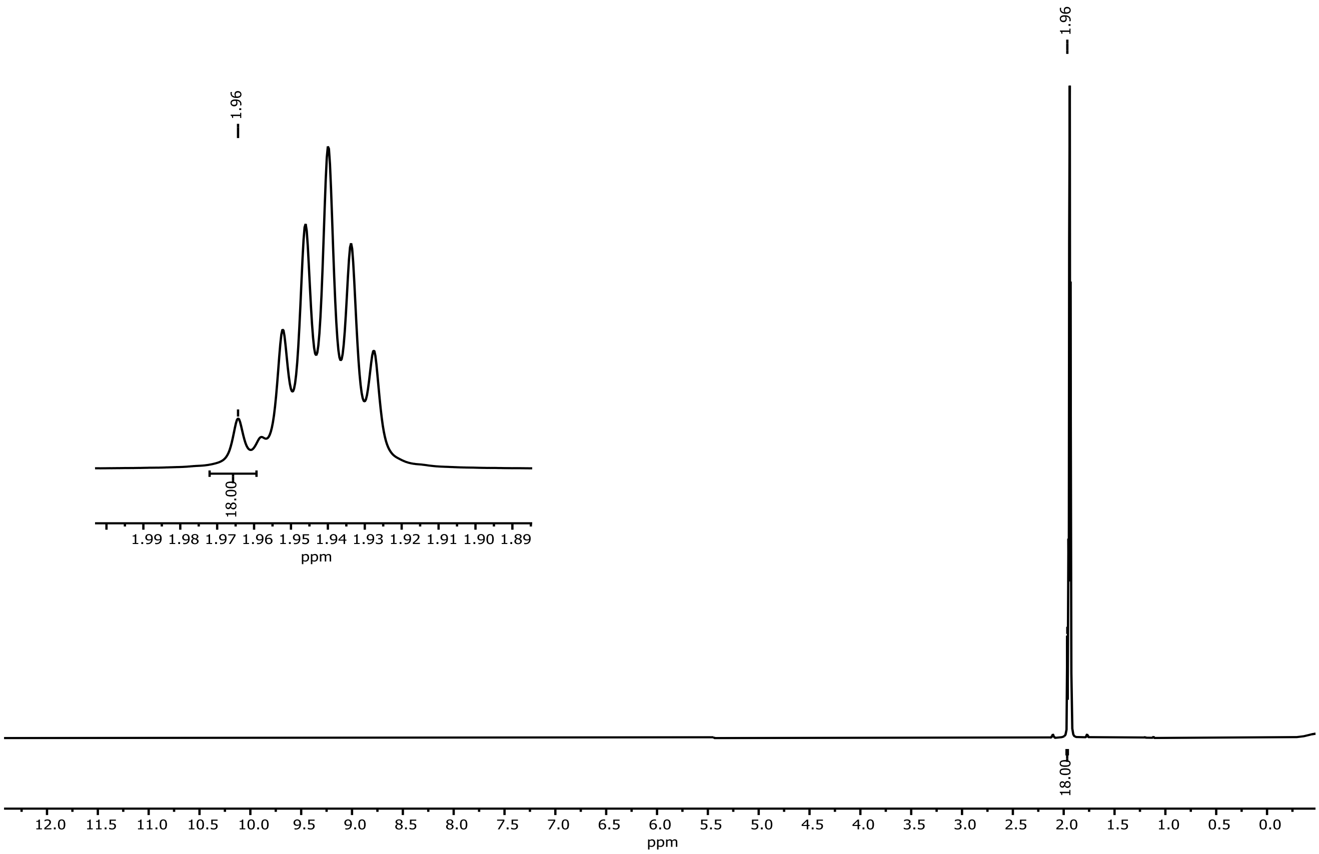

Figure S15. ^{1}H NMR (400 MHz, d_3-MeCN, rt) spectrum of $[Fe(MeCN)_6][C_5(CF_3)_5]_2$.

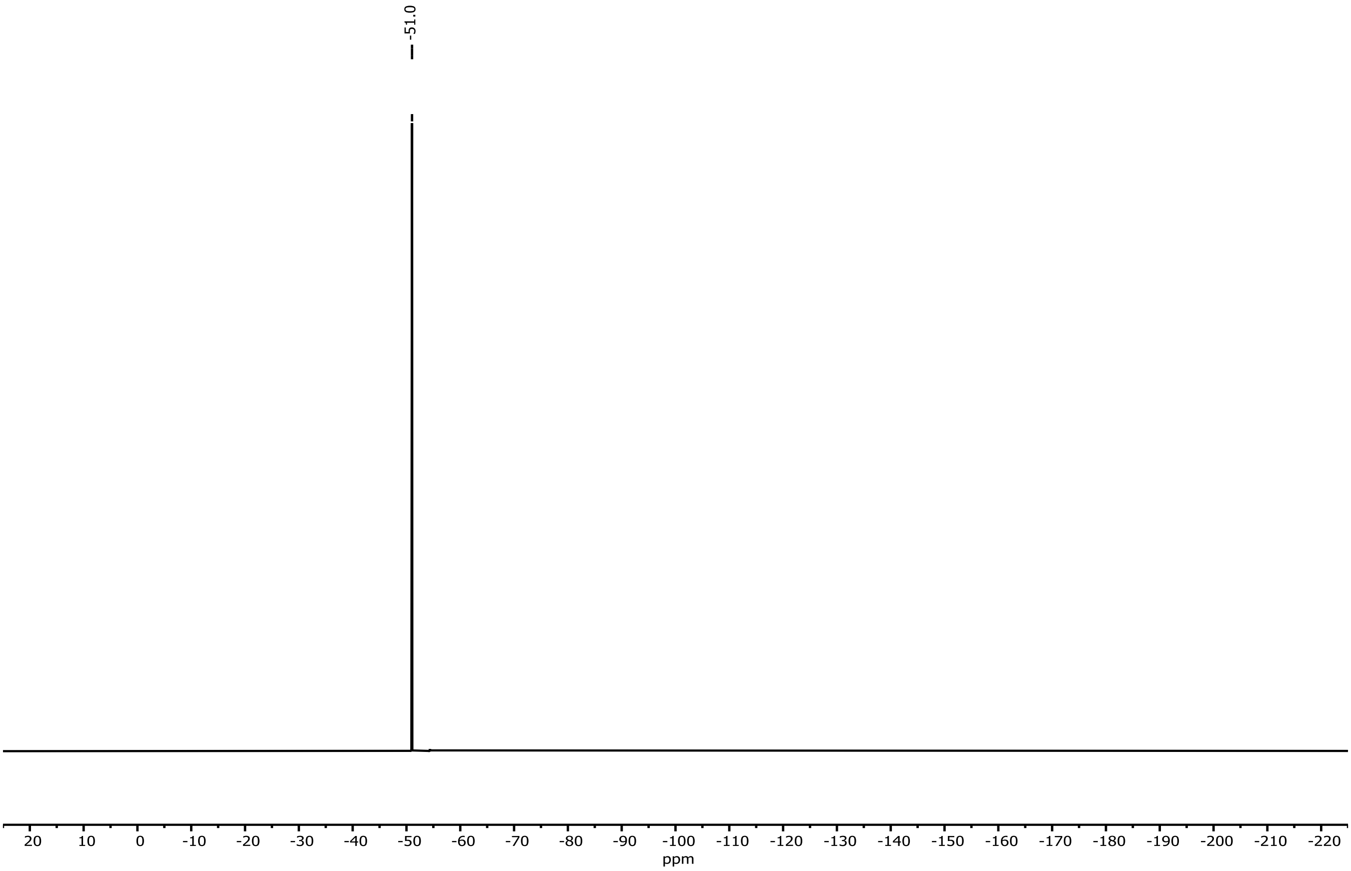

Figure S16. ^{19}F NMR (377 MHz, d_3-MeCN, rt) spectrum of $[Fe(MeCN)_6][C_5(CF_3)_5]_2$.

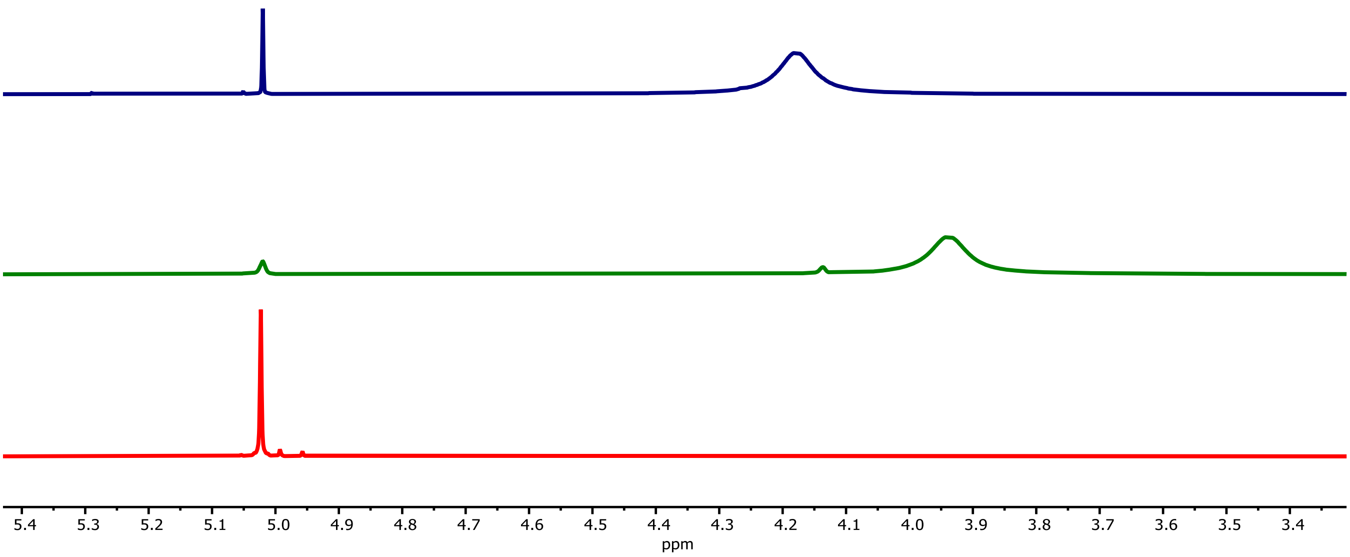

Figure S17. ^{1}H NMR (400 MHz, d_3-MeCN) spectra of [Fe(C_5H_5)($C_5(CF_3)_5$)] at rt (red), after 1 h of UV irradiation at –35 °C giving [Fe(C_5H_5)(d_3-MeCN)$_3$][$C_5(CF_3)_5$] (green) and after another 2 h at rt giving [Fe(C_5H_5)$_2$] (blue).

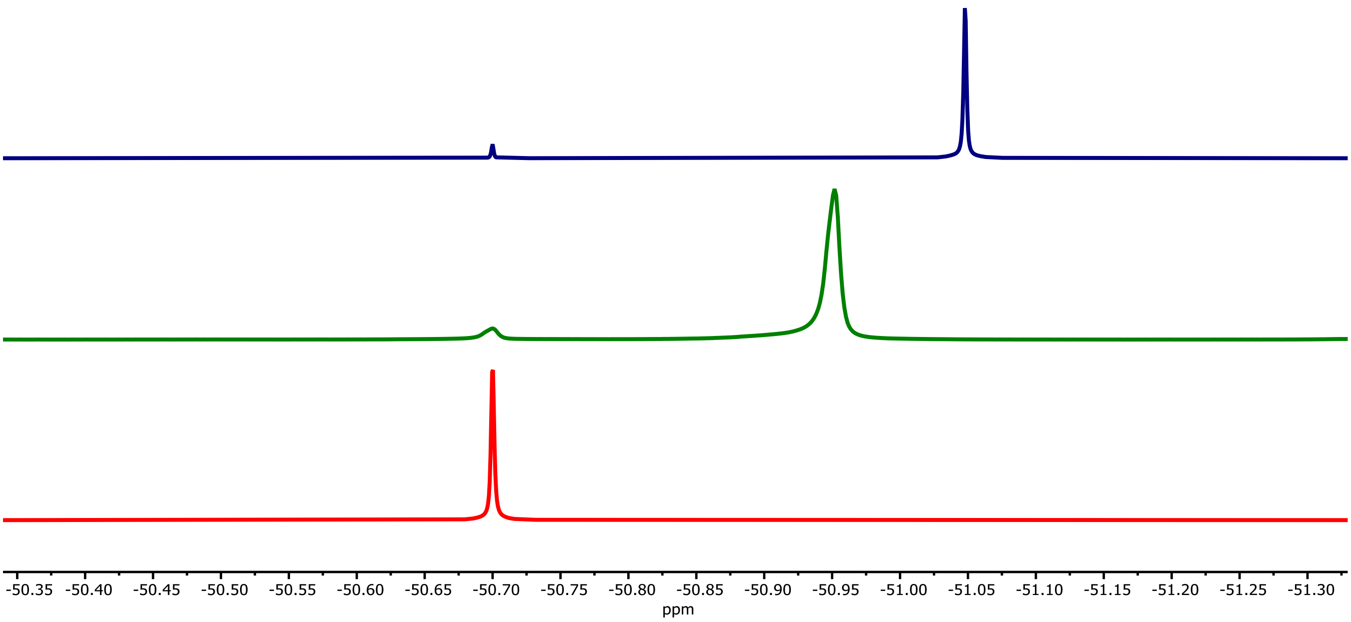

Figure S18. ^{19}F NMR (377 MHz, d_3-MeCN) spectra of [Fe(C_5H_5)($C_5(CF_3)_5$)] at rt (red), after 1 h of UV irradiation at –35 °C giving [Fe(C_5H_5)(d_3-MeCN)$_3$][$C_5(CF_3)_5$] (green) and after another 2 h at rt giving [Fe(C_5H_5)$_2$] (blue).

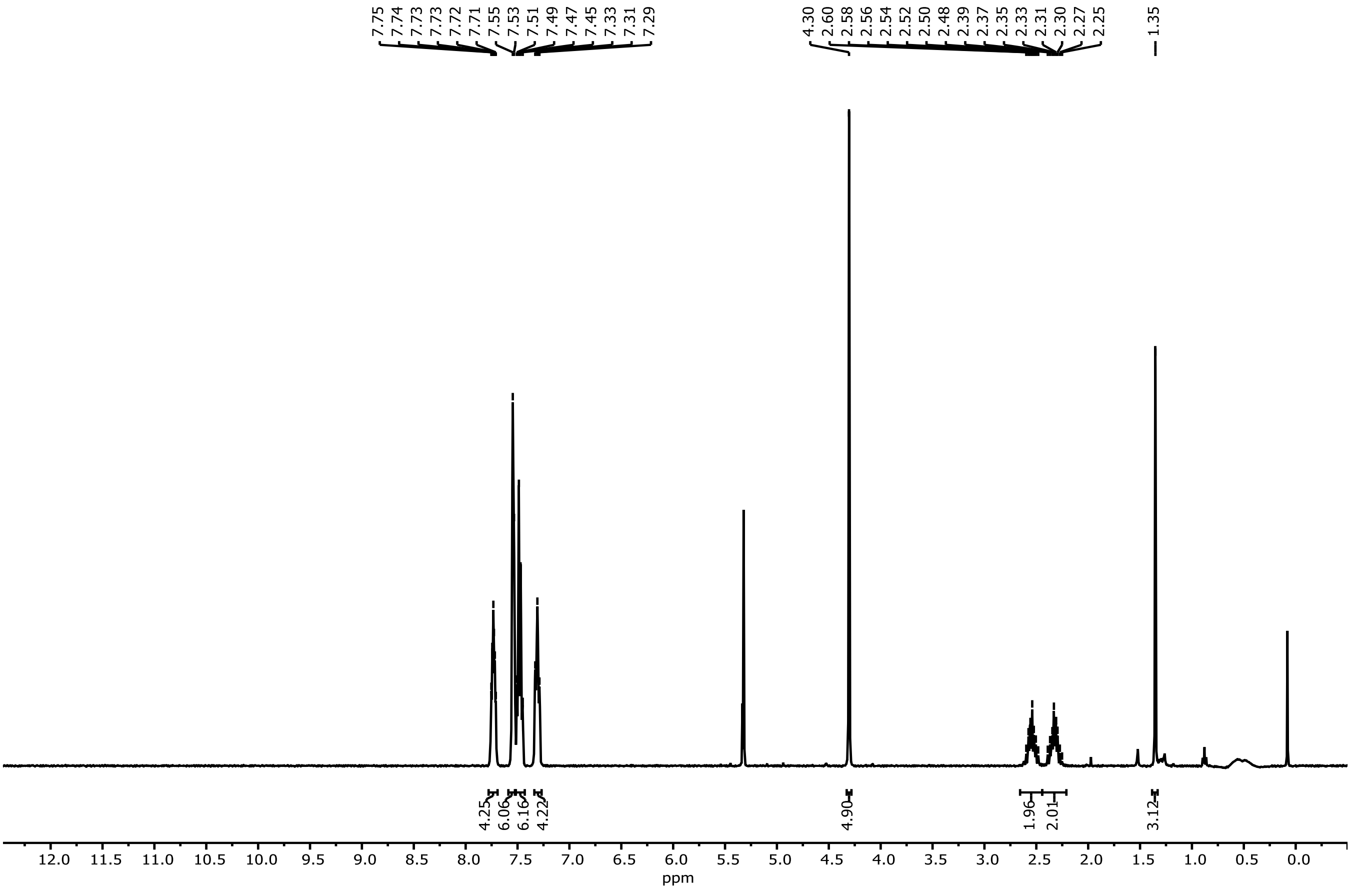

Figure S19. ^{1}H NMR (400 MHz, CD_2Cl_2, rt) spectrum of $[Fe(C_5H_5)(DPPE)(MeCN)][C_5(CF_3)_5]$.

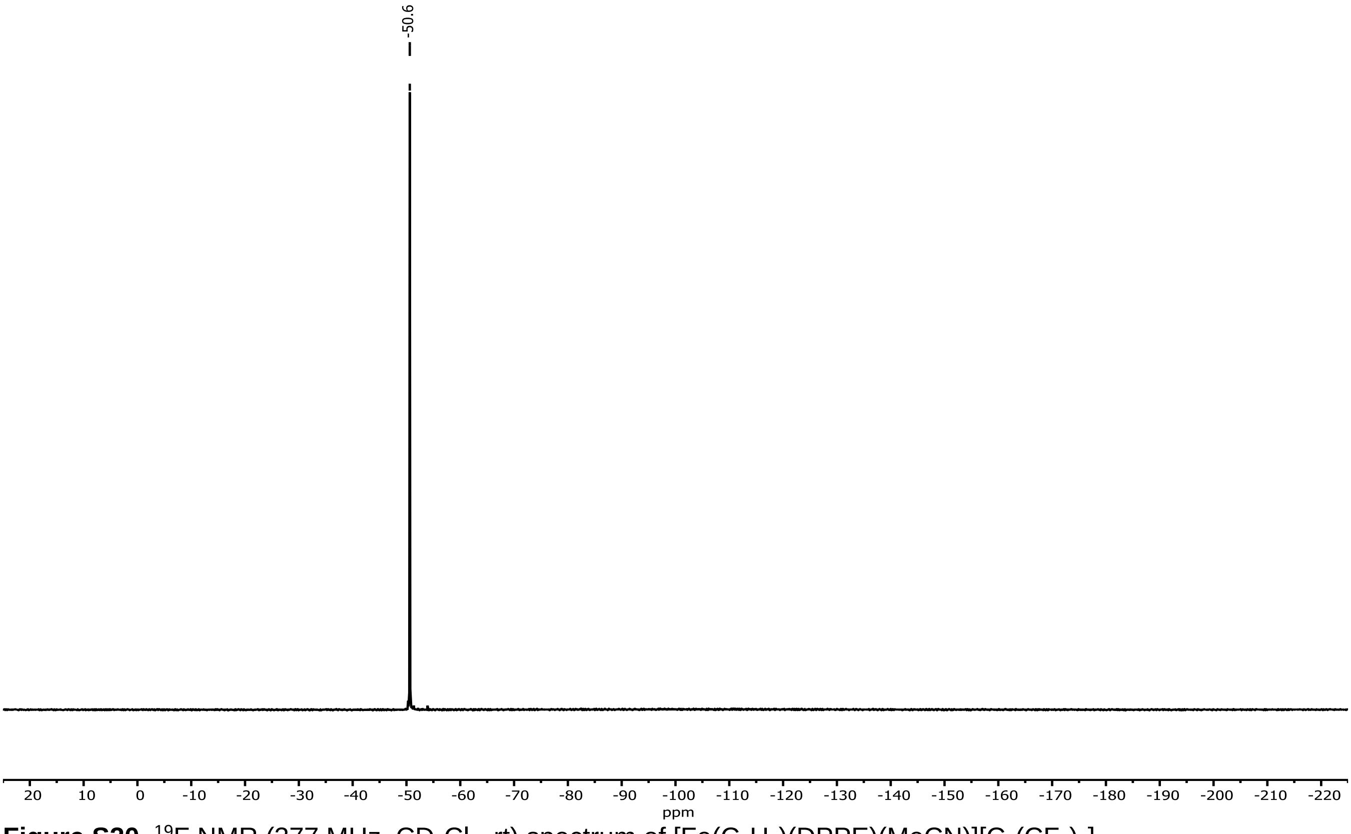

Figure S20. ^{19}F NMR (377 MHz, CD_2Cl_2, rt) spectrum of $[Fe(C_5H_5)(DPPE)(MeCN)][C_5(CF_3)_5]$.

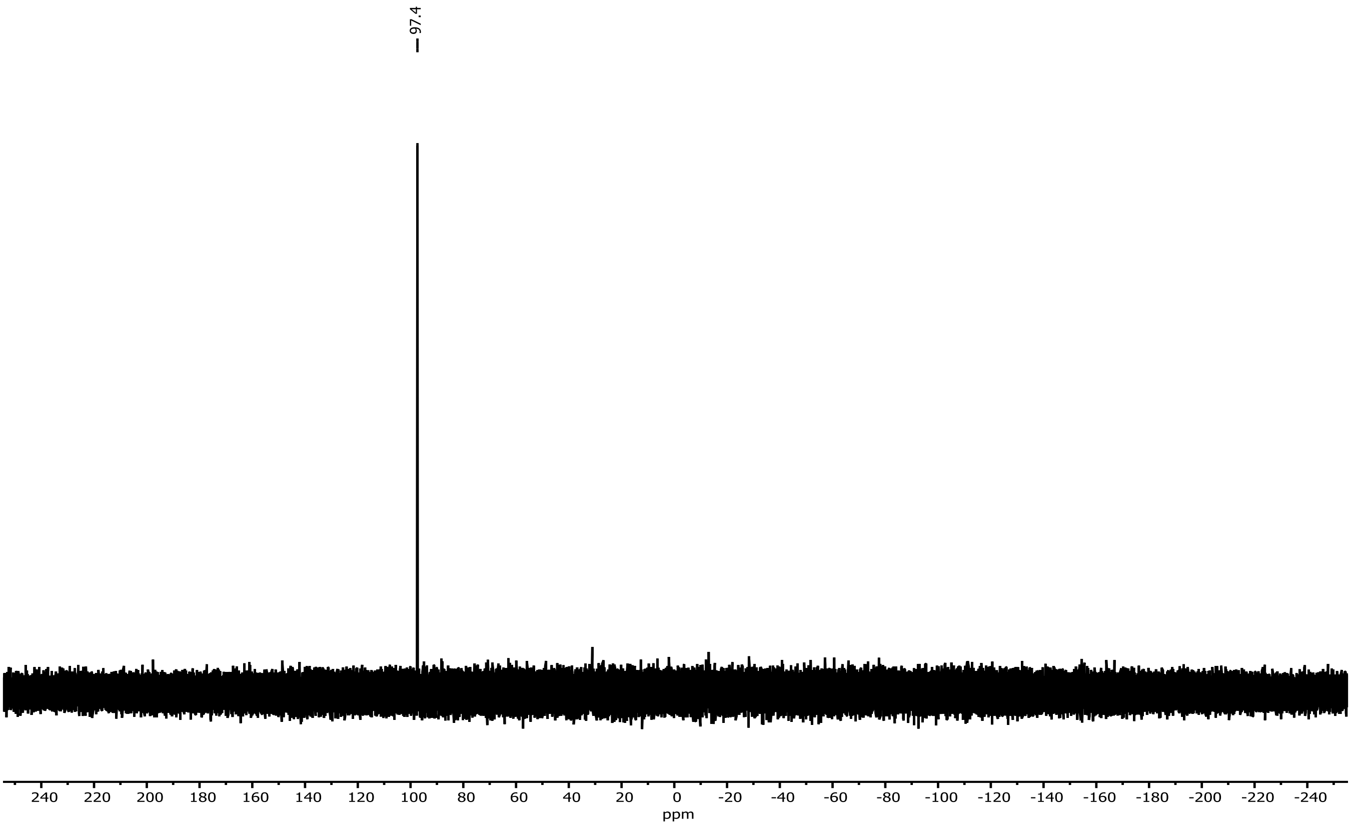

Figure S21. $^{31}P\{^1H\}$ NMR (162 MHz, CD_2Cl_2, rt) spectrum of $[Fe(C_5H_5)(DPPE)(MeCN)][C_5(CF_3)_5]$.

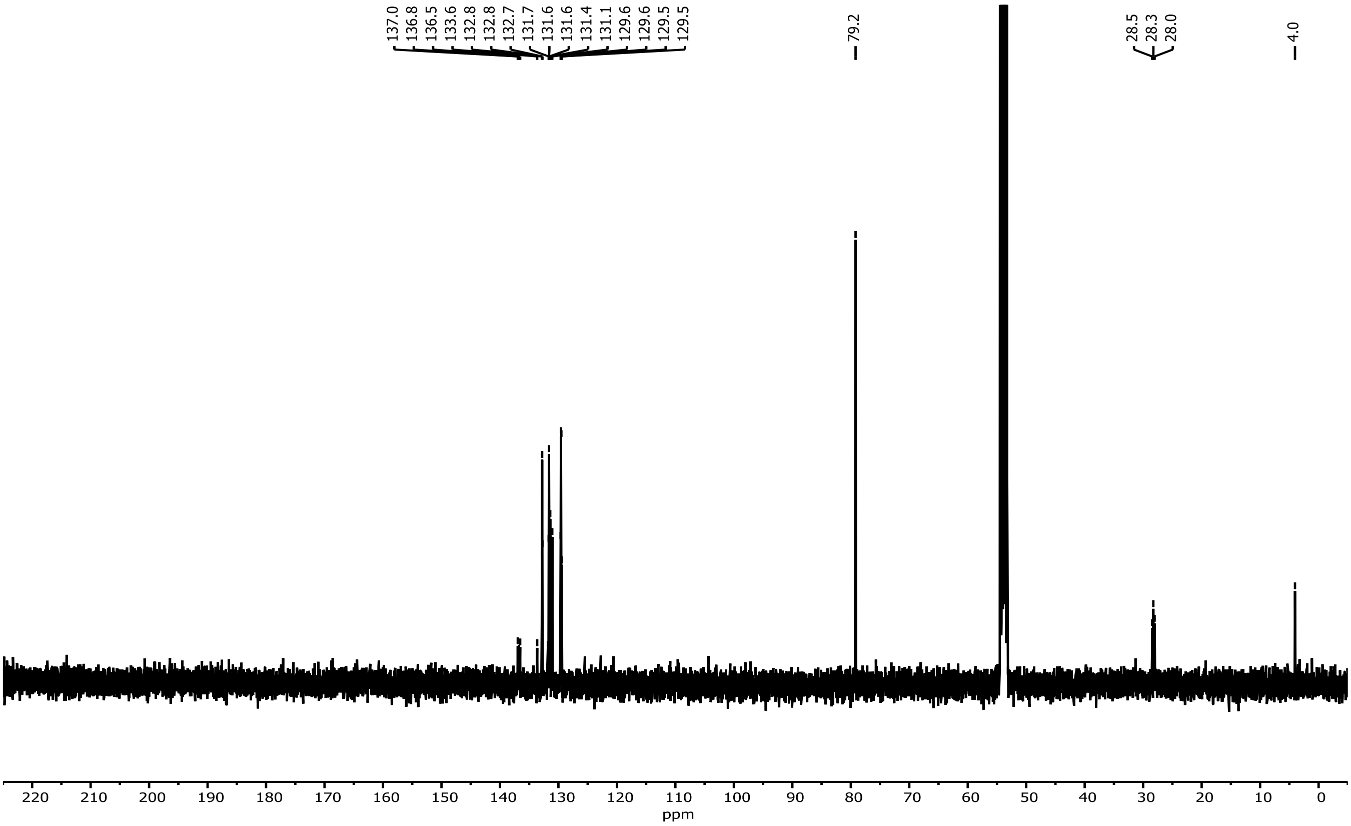

Figure S22. $^{13}C\{^1H\}$ NMR (100 MHz, CD_2Cl_2, rt) spectrum of $[Fe(C_5H_5)(DPPE)(MeCN)][C_5(CF_3)_5]$.

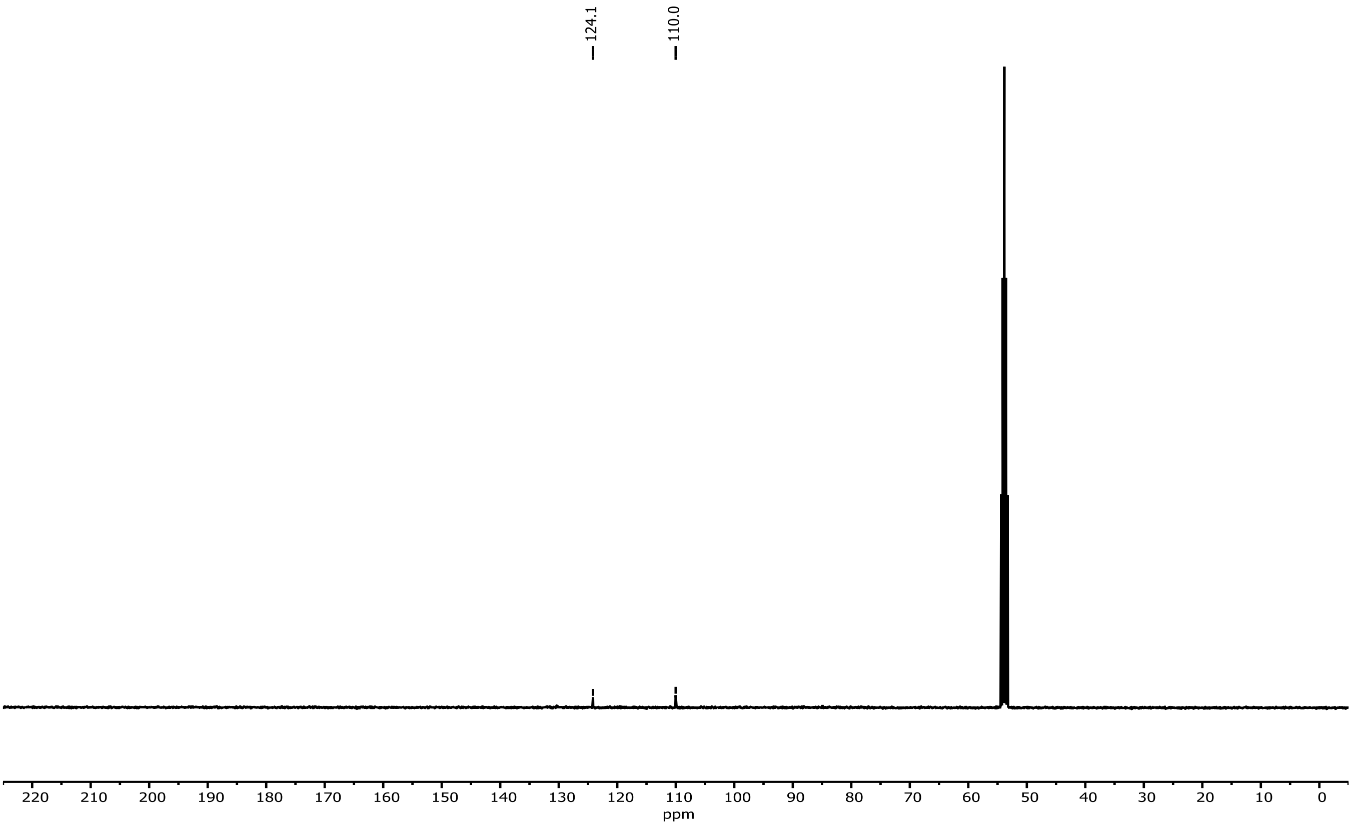

Figure S23. $^{13}C\{^{19}F\}$ NMR (162 MHz, CD_2Cl_2, rt) spectrum of $[Fe(C_5H_5)(DPPE)(MeCN)][C_5(CF_3)_5]$.

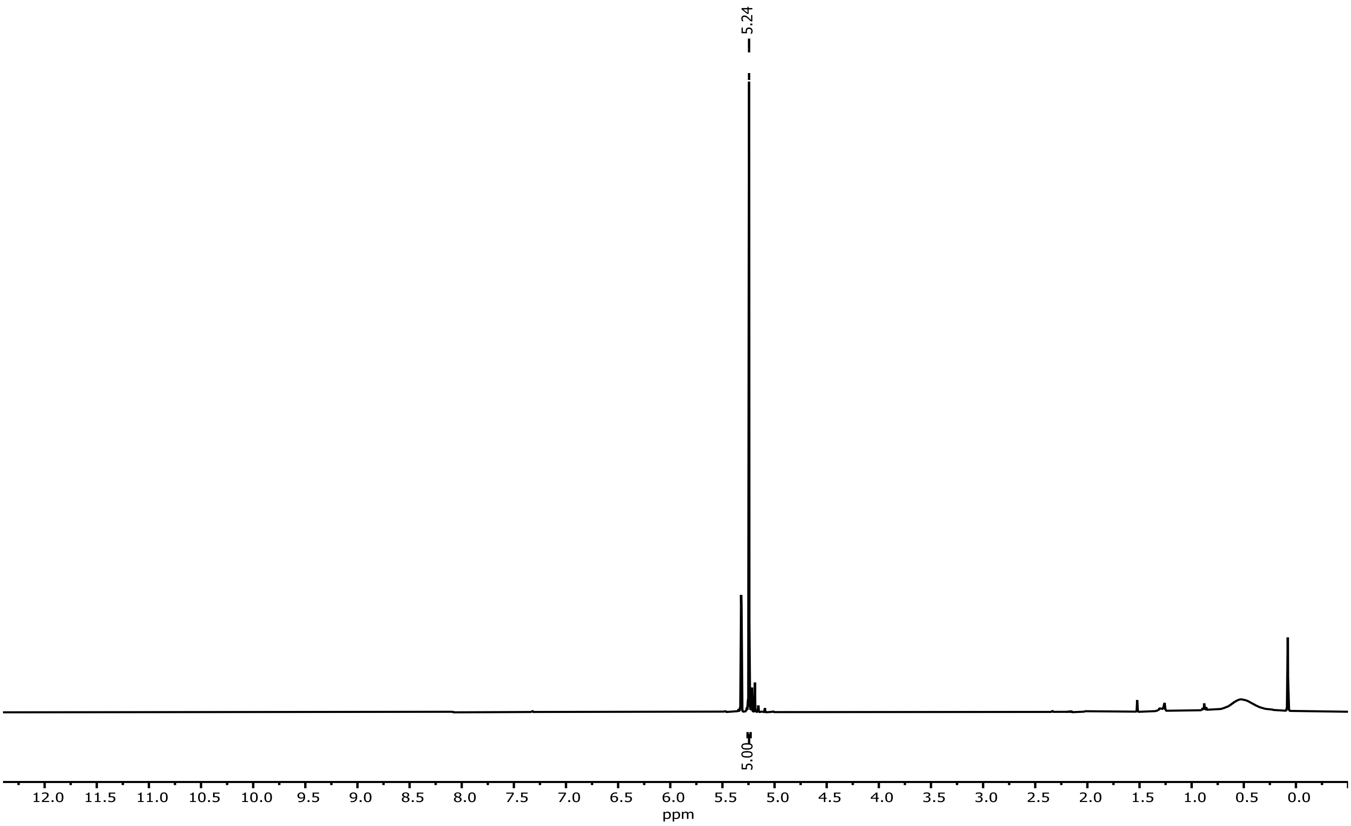

Figure S24. ^{1}H NMR (400 MHz, CD_2Cl_2, rt) spectrum of $[Ru(C_5H_5)(C_5(CF_3)_5)]$.

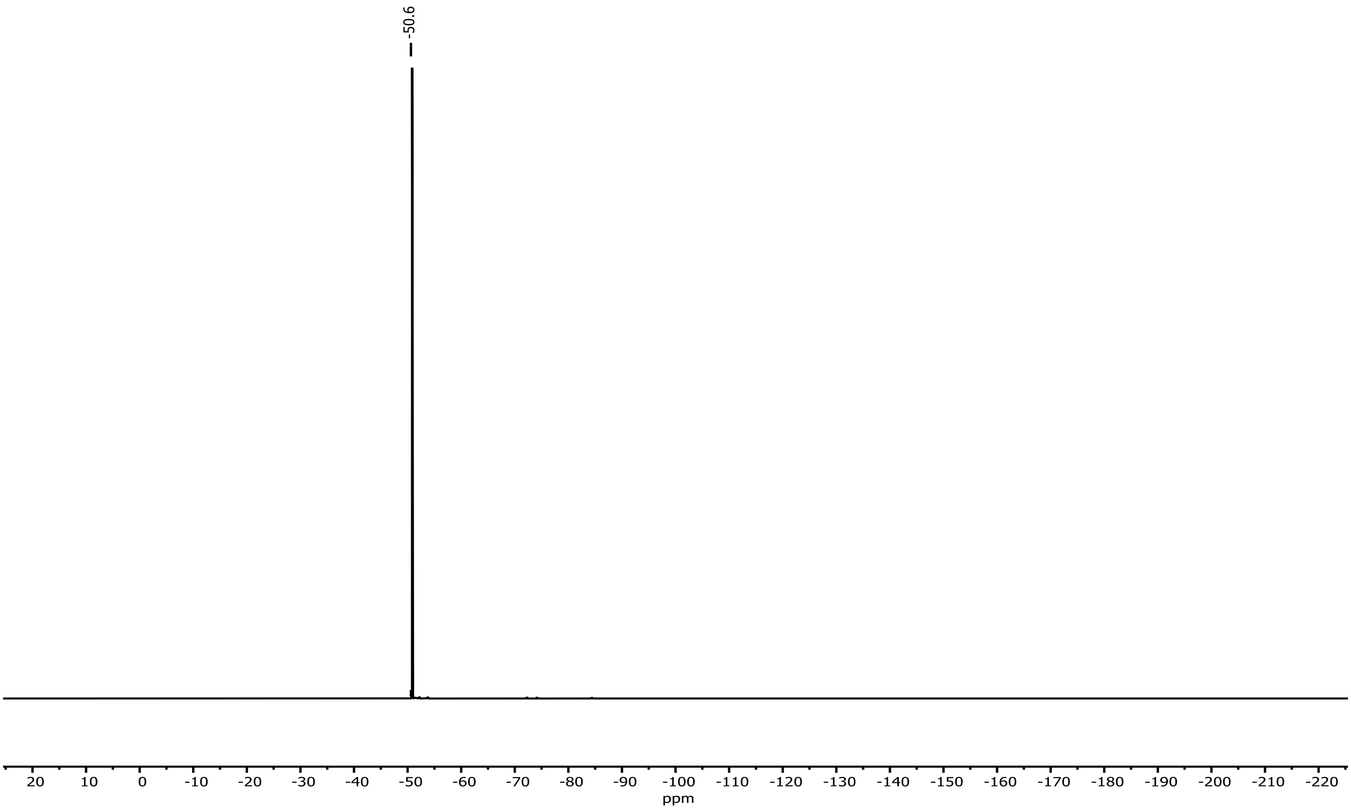

Figure S25. ^{19}F NMR (377 MHz, CD_2Cl_2, rt) spectrum of [Ru(C_5H_5)($C_5(CF_3)_5$)].

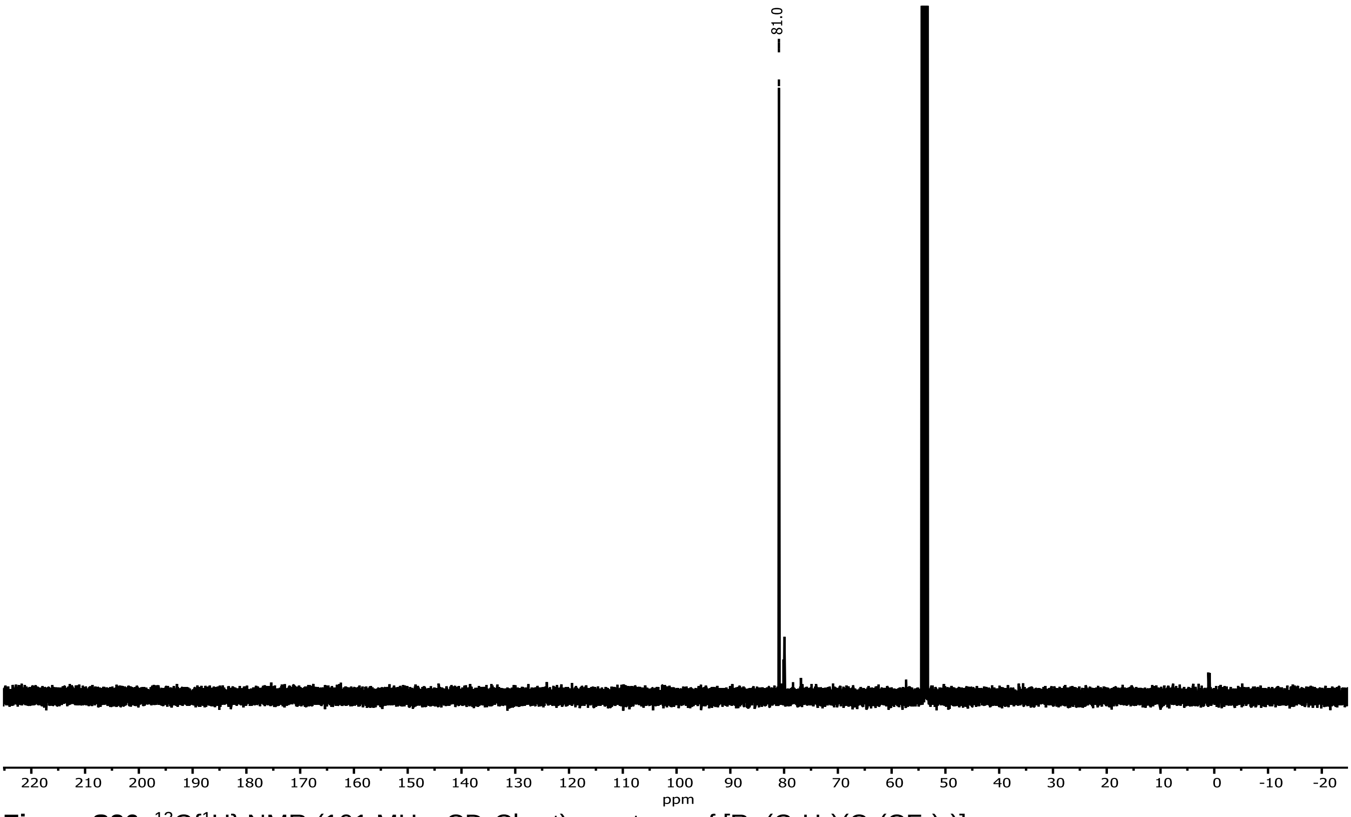

Figure S26. $^{13}C\{^1H\}$ NMR (101 MHz, CD_2Cl_2, rt) spectrum of [Ru(C_5H_5)($C_5(CF_3)_5$)].

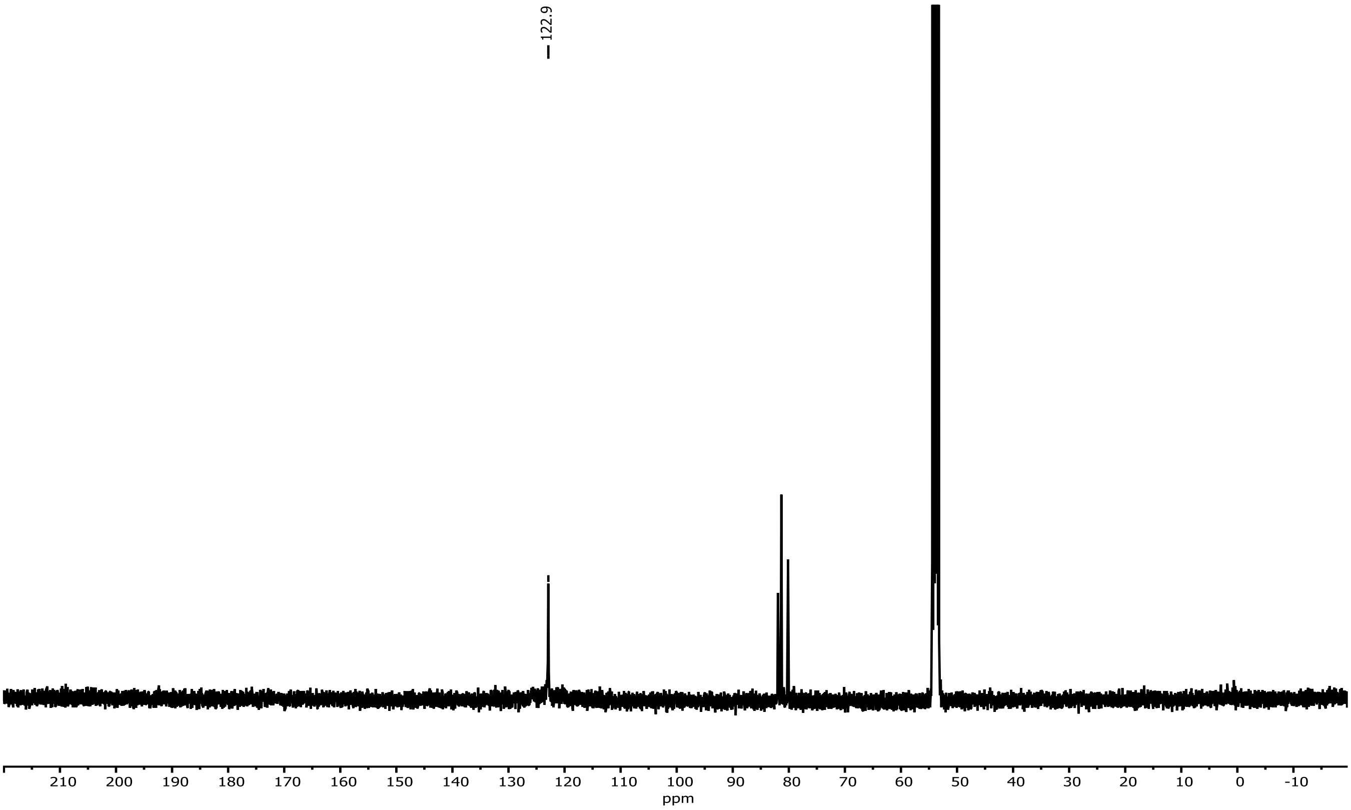

Figure S27. $^{13}C\{^{19}F\}$ NMR (101 MHz, CD_2Cl_2, rt) spectrum of [Ru(C_5H_5)($C_5(CF_3)_5$)].

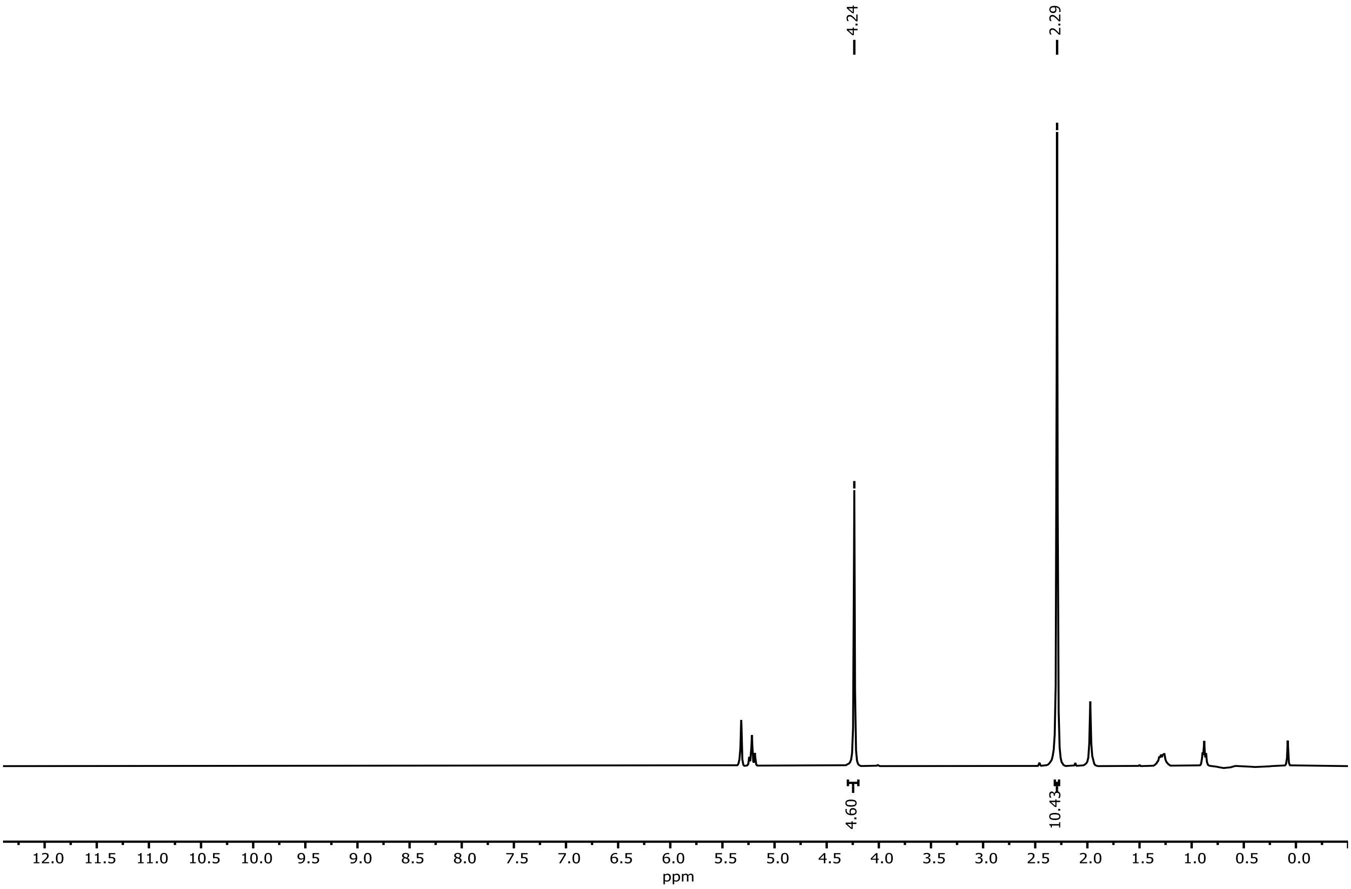

Figure S28. ^{1}H NMR (400 MHz, CD_2Cl_2, rt) spectrum of [Ru(C_5H_5)(MeCN)$_3$][$C_5(CF_3)_5$].

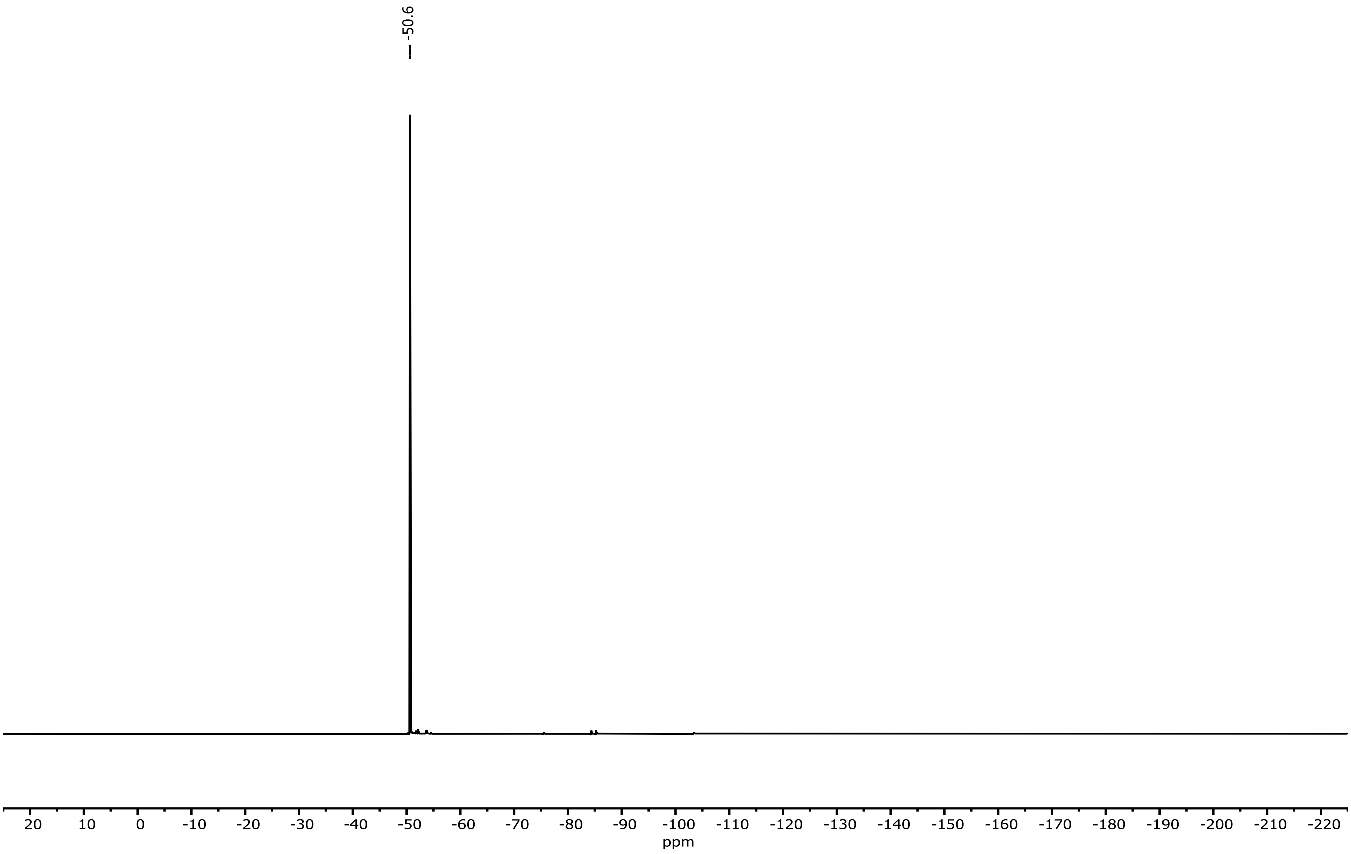

Figure S29. ^{19}F NMR (377 MHz, CD_2Cl_2, rt) spectrum of $[Ru(C_5H_5)(MeCN)_3][C_5(CF_3)_5]$.

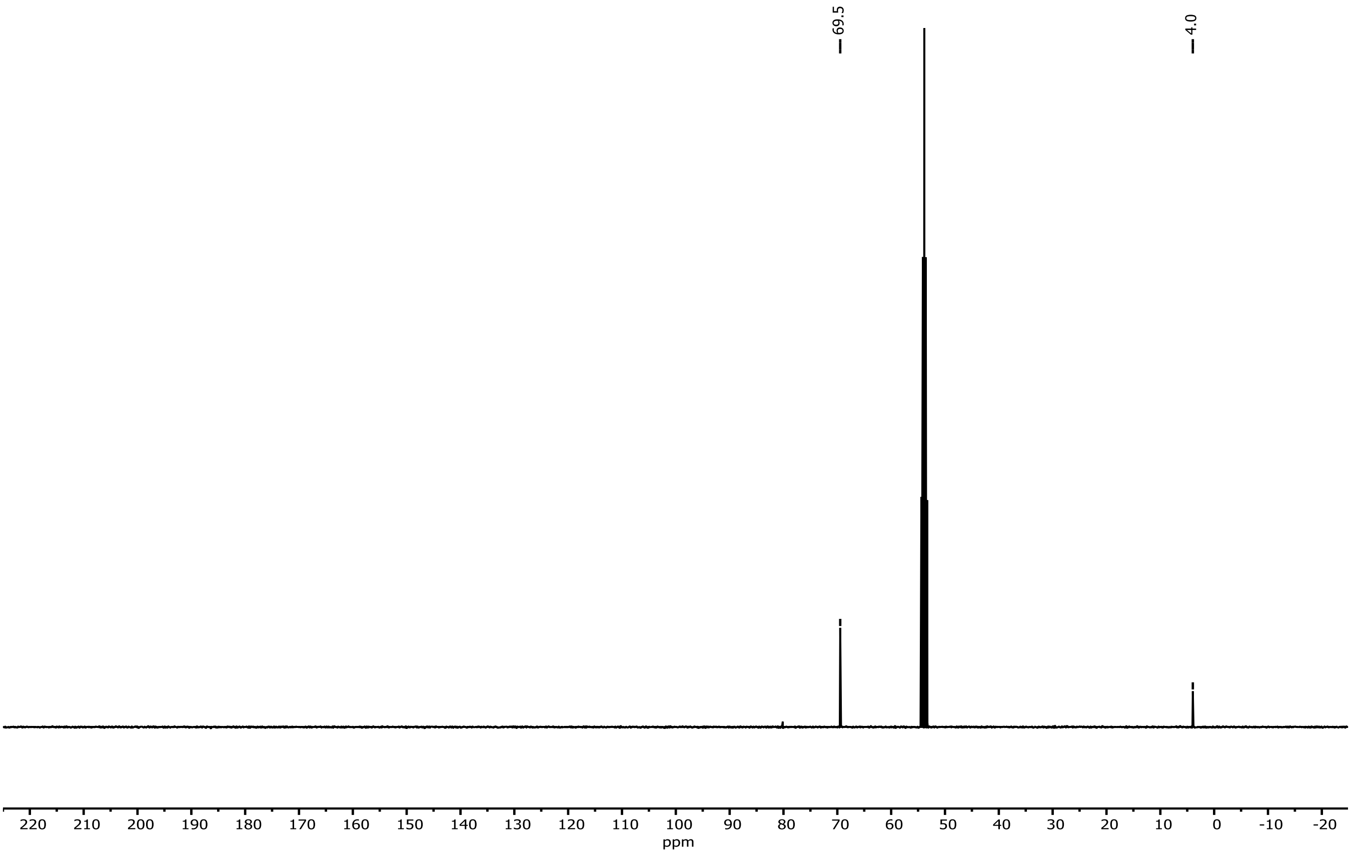

Figure S30. $^{13}C\{^1H\}$ NMR (100 MHz, CD_2Cl_2, rt) spectrum of $[Ru(C_5H_5)(MeCN)_3][C_5(CF_3)_5]$.

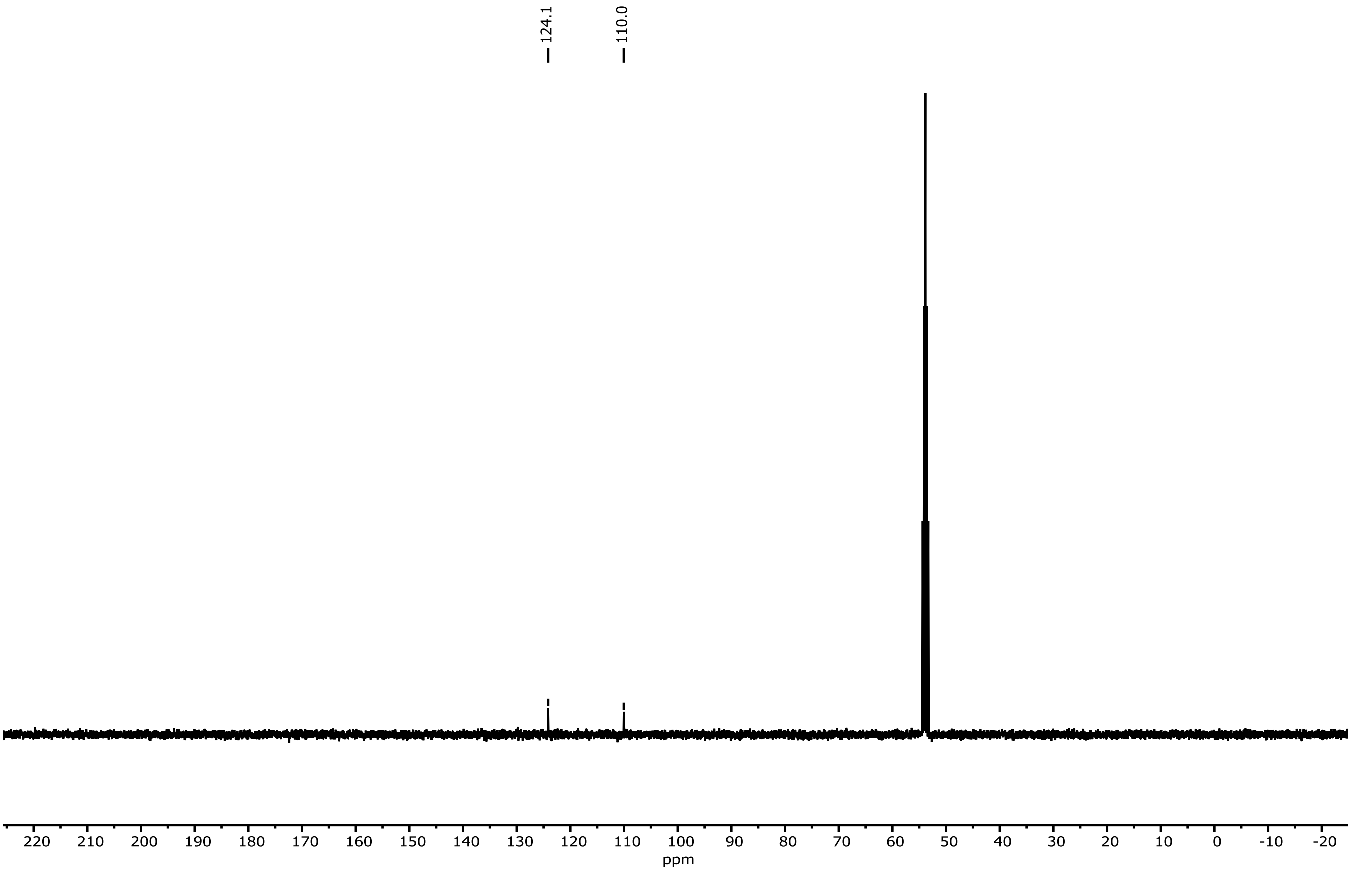

Figure S31. $^{13}C\{^{19}F\}$ NMR (100 MHz, CD_2Cl_2, rt) spectrum of [Ru(C_5H_5)(MeCN)$_3$][$C_5(CF_3)_5$].

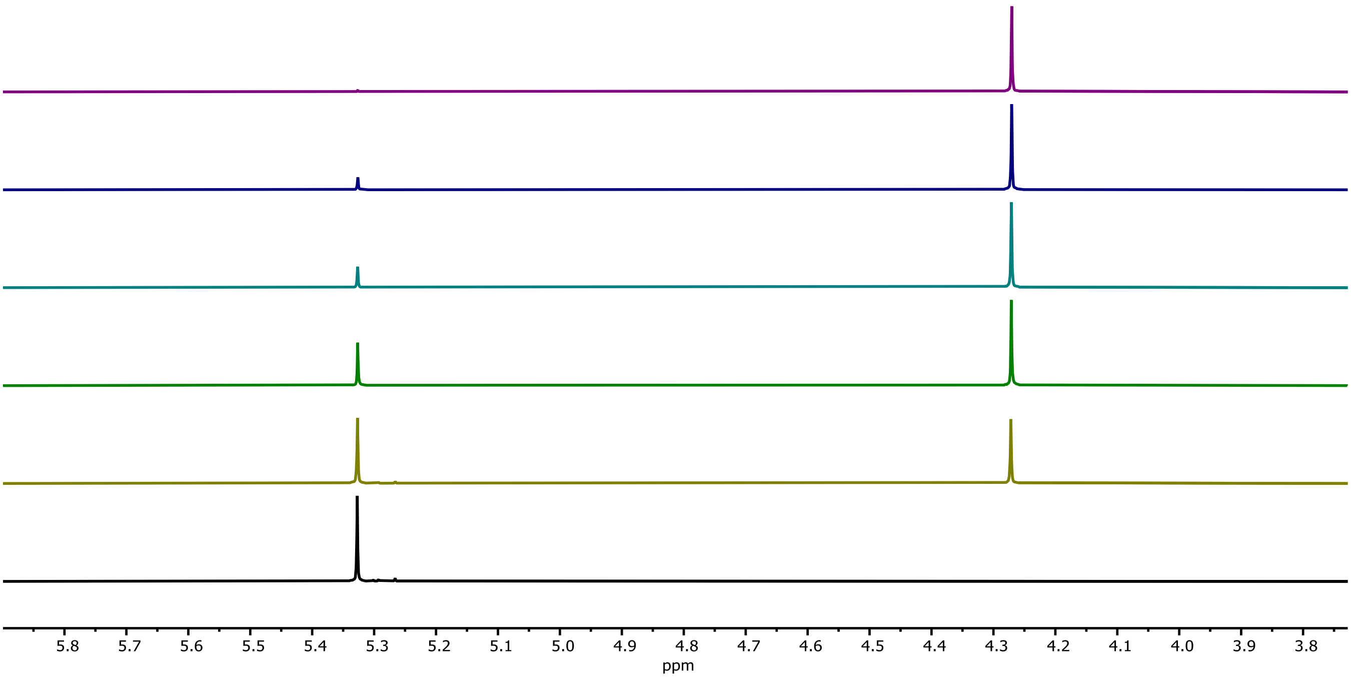

Figure S32. ^{1}H NMR (400 MHz, d_3-MeCN, rt) spectra of [Ru(C_5H_5)($C_5(CF_3)_5$)] after 0 h (black), 2 h (yellow), 4 h (green), 8 h (light blue), 16 h (dark blue) and 24 h (purple) of UV irradiation at room temperature giving [Ru(C_5H_5)(d_3-MeCN)$_3$][$C_5(CF_3)_5$].

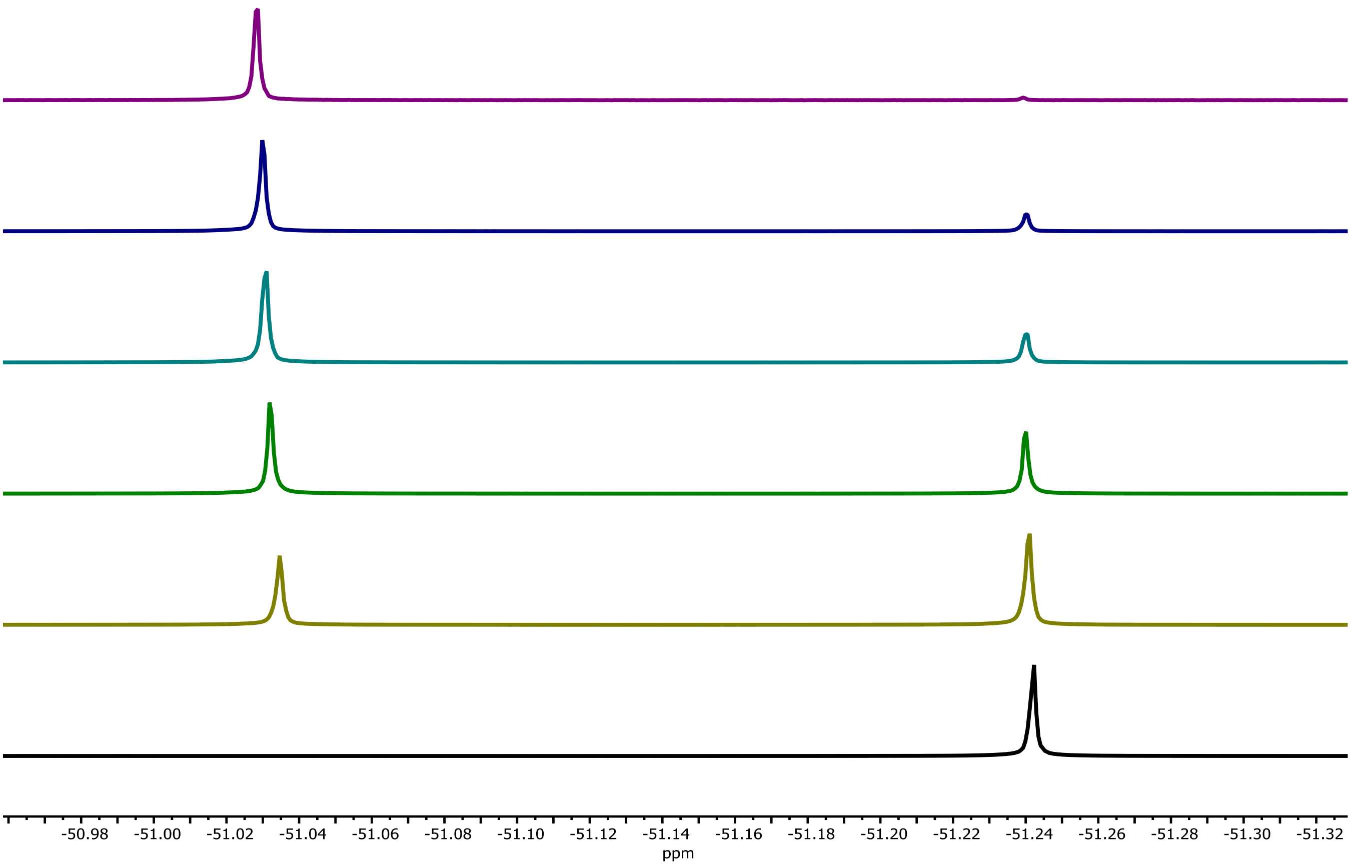

Figure S33. ^{19}F NMR (377 MHz, *d*$_3$-MeCN, rt) spectra of [Ru(C_5H_5)($C_5(CF_3)_5$)] after 0 h (black), 2 h (yellow), 4 h (green), 8 h (light blue), 16 h (dark blue) and 24 h (purple) of UV irradiation at room temperature giving [Ru(C_5H_5)(*d*$_3$-MeCN)$_3$][$C_5(CF_3)_5$].

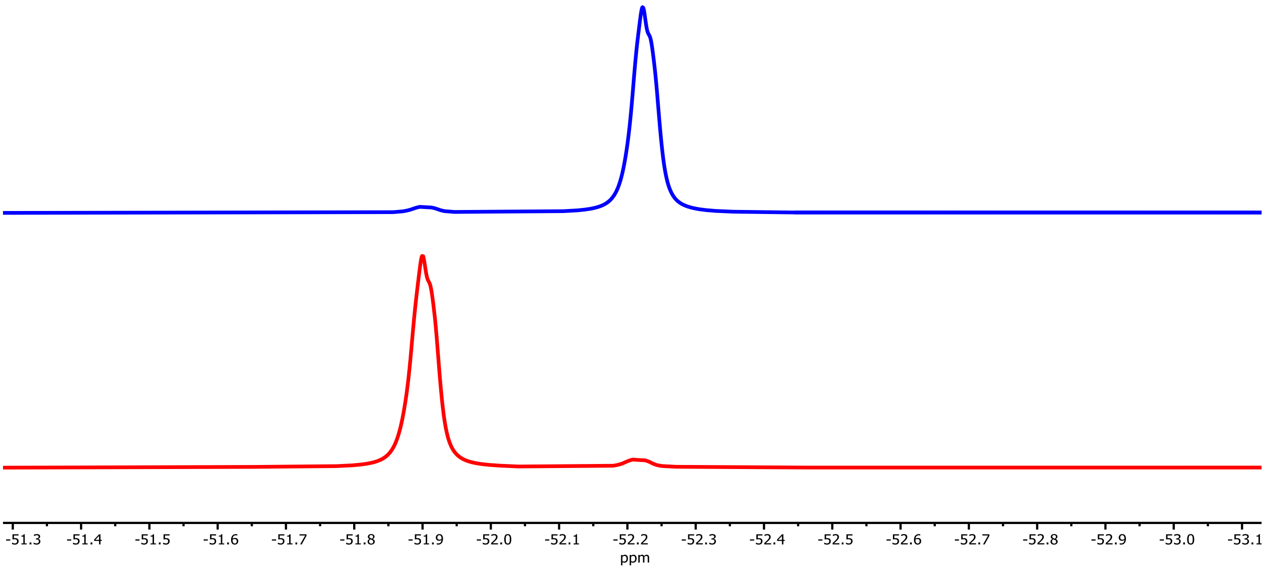

Figure S34. ^{19}F NMR (377 MHz, 1,2-DCE, rt) spectra of [Ru(C_5H_5)(MeCN)$_3$][$C_5(CF_3)_5$] after 0 h (red) and 2 h (blue) of heating at 80 °C giving [Ru(C_5H_5)($C_5(CF_3)_5$)].

IR Spectra

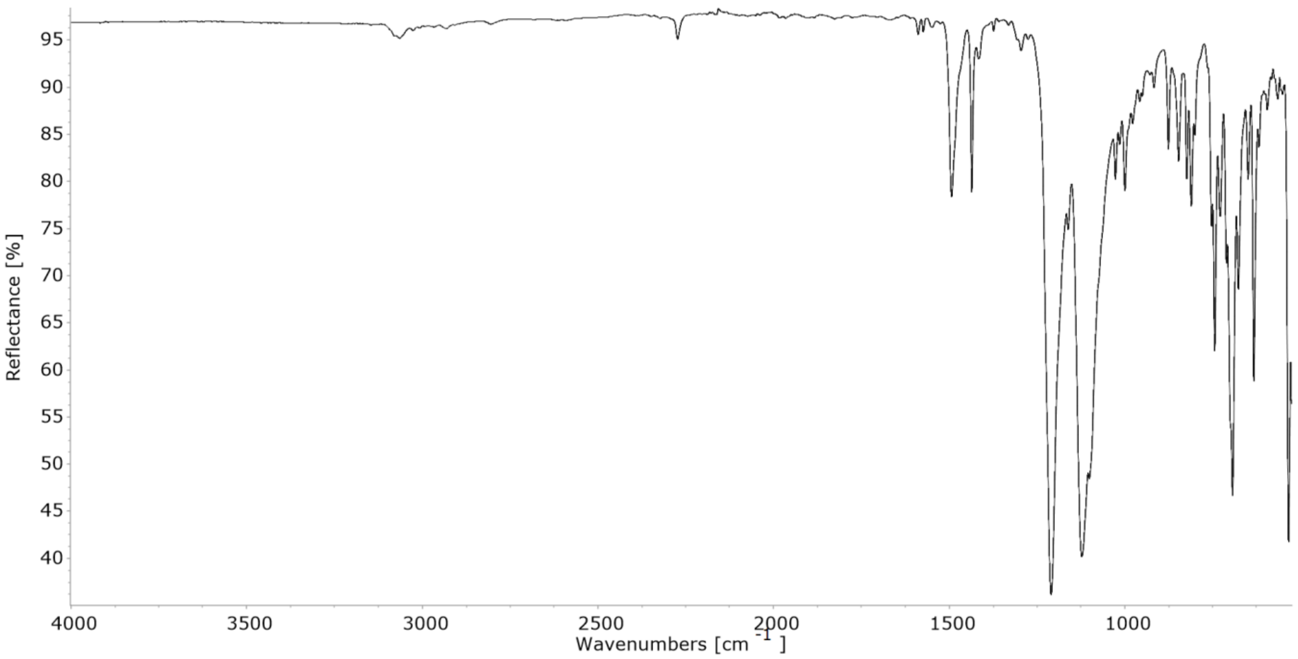

Figure 35. IR (ATR, rt) spectrum of $[Fe(C_5H_5)(DPPE)(MeCN)][C_5(CF_3)_5]$.

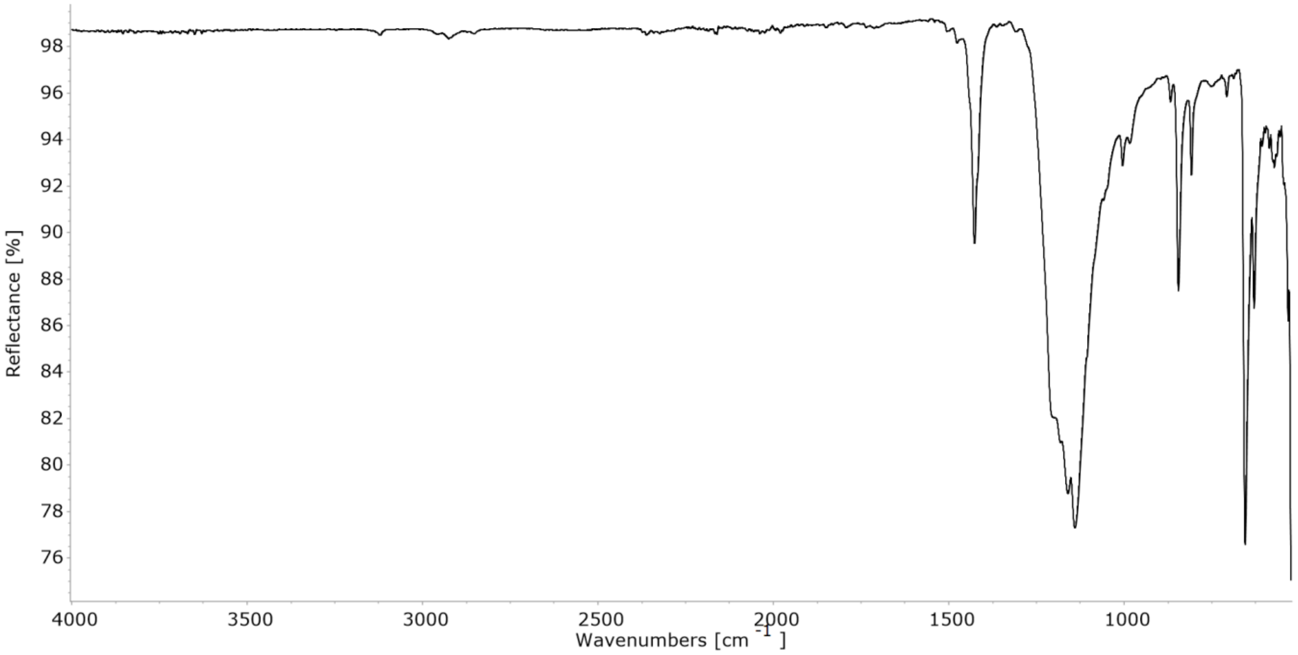

Figure 36. IR (ATR, rt) spectrum of $[Ru(C_5H_5)(C_5(CF_3)_5)]$.

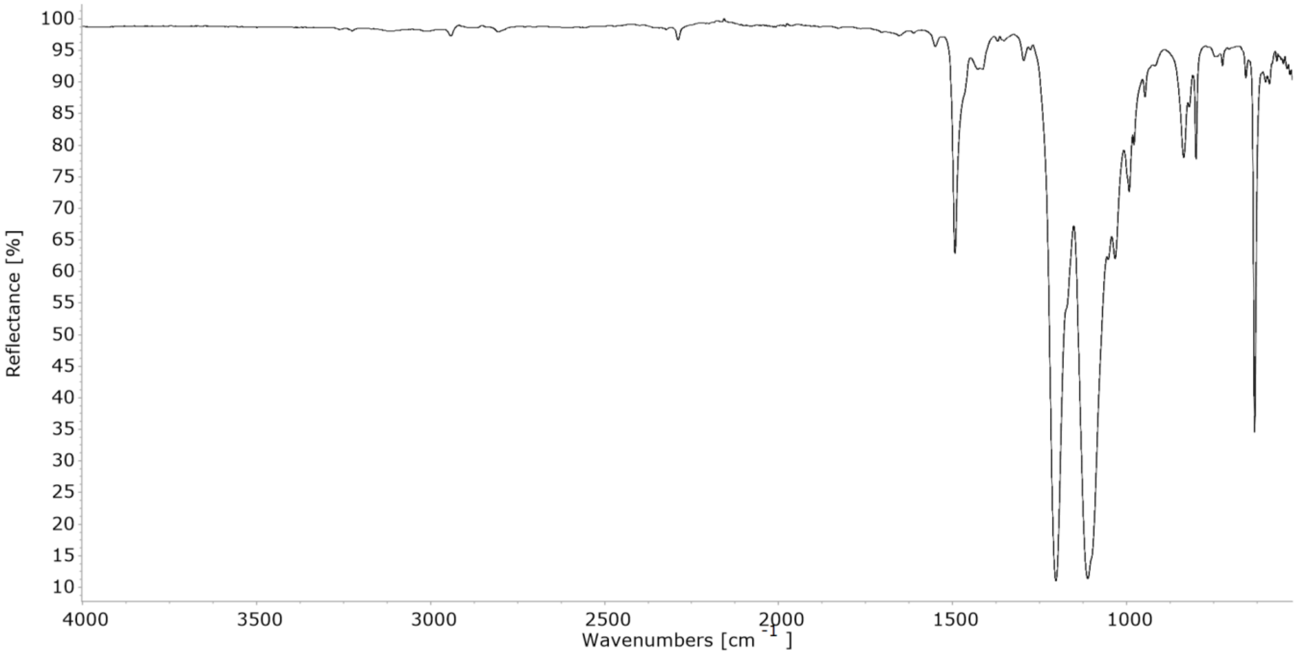

Figure 37. IR (ATR, rt) spectrum of $[Ru(C_5H_5)(MeCN)_3][C_5(CF_3)_5]$

UV/VIS Spectra

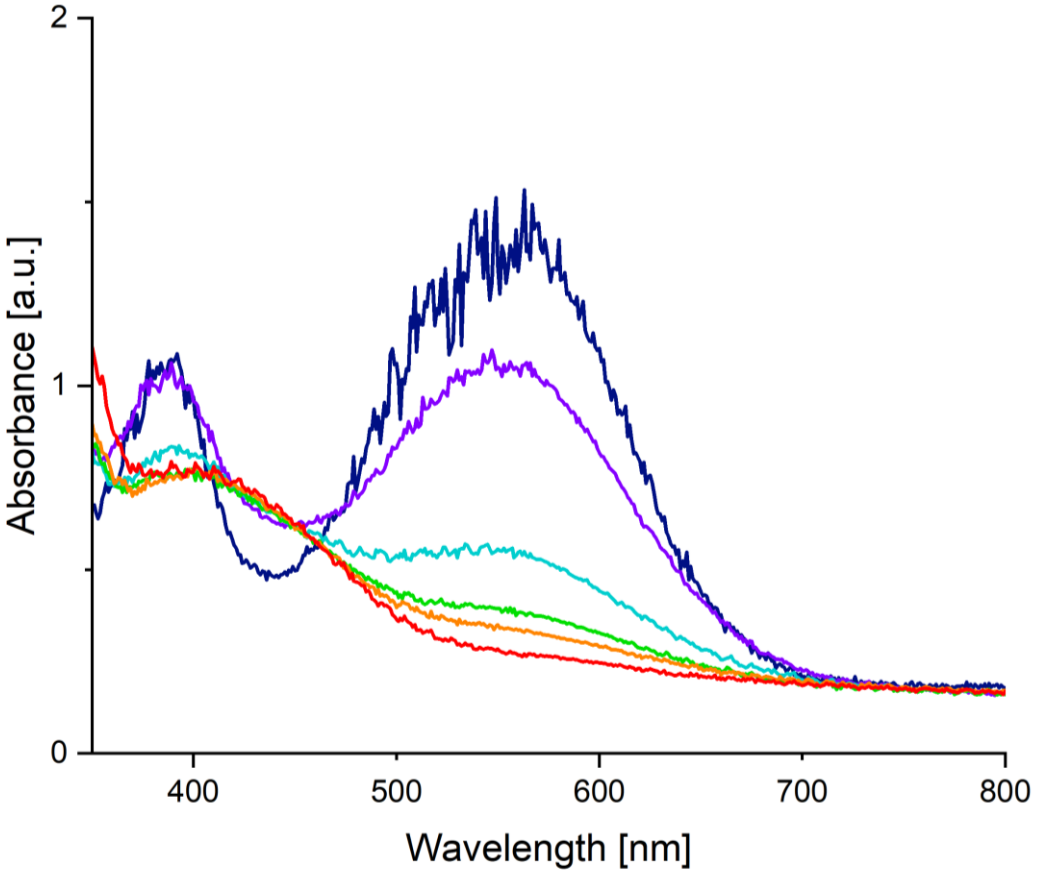

Figure S38. UV/VIS spectra (MeCN, rt) of $[Fe(C_5H_5)(MeCN)_3][C_5(CF_3)_5]$ after 0 min (dark blue), 10 min (violet), 20 min (light blue), 30 min (green), 40 min (orange), 75 min (red).

Crystallographic Data

Table S1. Crystallographic data of $[Fe(C_5H_5)(DPPE)(MeCN)][C_5(CF_3)_5]\cdot 2\ CH_2Cl_2$.

Identification code	2440856
Empirical formula	$C_{45}H_{36}Cl_4F_{15}FeNP_2$
Formula weight	1135.34
Temperature/K	100.00
Crystal system	monoclinic
Space group	*Pc*
a/Å	15.9310(9)
b/Å	15.9306(11)
c/Å	18.5289(13)
α/°	90
β/°	89.979(3)
γ/°	90
Volume/Å^3	4702.5(5)
Z	4
ρ_{calc}g/cm^3	1.604
μ/mm^{-1}	0.711
F(000)	2288.0
Crystal size/mm^3	0.1 × 0.1 × 0.1
Radiation	MoKα (λ = 0.71073)
2Θ range for data collection/°	3.616 to 52.782
Index ranges	-19 ≤ h ≤ 19, -19 ≤ k ≤ 19, -23 ≤ l ≤ 23
Reflections collected	77532
Independent reflections	19069 [R_{int} = 0.0551, R_{sigma} = 0.0503]
Data/restraints/parameters	19069/2/1326
Goodness-of-fit on F^2	0.842
Final R indexes [I>=2σ (I)]	R_1 = 0.0563, wR_2 = 0.1715
Final R indexes [all data]	R_1 = 0.0681, wR_2 = 0.1968
Largest diff. peak/hole / e Å^{-3}	1.01/-0.89
Flack parameter	0.046(6)

Table S2. Crystallographic data of $[Ru(C_5H_5)(C_5(CF_3)_5)]$.

Identification code	2440857
Empirical formula	$C_{15}H_5F_{15}Ru$
Formula weight	571.26
Temperature/K	100.15
Crystal system	monoclinic
Space group	$P2_1/c$
a/Å	7.3204(3)
b/Å	15.6095(7)
c/Å	14.6724(7)
α/°	90
β/°	90.01(2)
γ/°	90
Volume/Å^3	1676.58(13)
Z	4
ρ_{calc}g/cm^3	2.263
μ/mm^{-1}	1.095
F(000)	1096.0
Crystal size/mm^3	0.532 × 0.168 × 0.092
Radiation	MoKα (λ = 0.71073)
2Θ range for data collection/°	2.61 to 61.056
Index ranges	-10 ≤ h ≤ 10, -22 ≤ k ≤ 19, -20 ≤ l ≤ 16
Reflections collected	14655
Independent reflections	5034 [R_{int} = 0.0497, R_{sigma} = 0.0581]
Data/restraints/parameters	5034/48/294
Goodness-of-fit on F^2	1.091
Final R indexes [I>=2σ (I)]	R_1 = 0.0785, wR_2 = 0.1969
Final R indexes [all data]	R_1 = 0.0993, wR_2 = 0.2176
Largest diff. peak/hole / e Å^{-3}	1.35/-1.07

Table S3. Crystallographic data of $[Ru(C_5H_5)(MeCN)_3][C_5(CF_3)_5]\cdot CH_2Cl_2$.

Identification code	2440858
Empirical formula	$C_{22}H_{14}Cl_2F_{15}N_3Ru$
Formula weight	777.33
Temperature/K	100.00
Crystal system	monoclinic
Space group	$P2_1$
a/Å	11.6437(10)
b/Å	11.1515(9)
c/Å	12.2040(11)
α/°	90
β/°	116.484(3)
γ/°	90
Volume/Å^3	1418.3(2)
Z	2
ρ_{calc}g/cm^3	1.820
μ/mm^{-1}	0.859
F(000)	760.0
Crystal size/mm^3	0.1 × 0.1 × 0.1
Radiation	MoKα (λ = 0.71073)
2Θ range for data collection/°	5.22 to 50.088
Index ranges	-13 ≤ h ≤ 13, -13 ≤ k ≤ 13, -14 ≤ l ≤ 14
Reflections collected	24628
Independent reflections	5005 [R_{int} = 0.0490, R_{sigma} = 0.0349]
Data/restraints/parameters	5005/16/389
Goodness-of-fit on F^2	1.084
Final R indexes [I>=2σ (I)]	R_1 = 0.0415, wR_2 = 0.0956
Final R indexes [all data]	R_1 = 0.0501, wR_2 = 0.1056
Largest diff. peak/hole / e Å^{-3}	1.21/-0.65
Flack parameter	0.47(6)

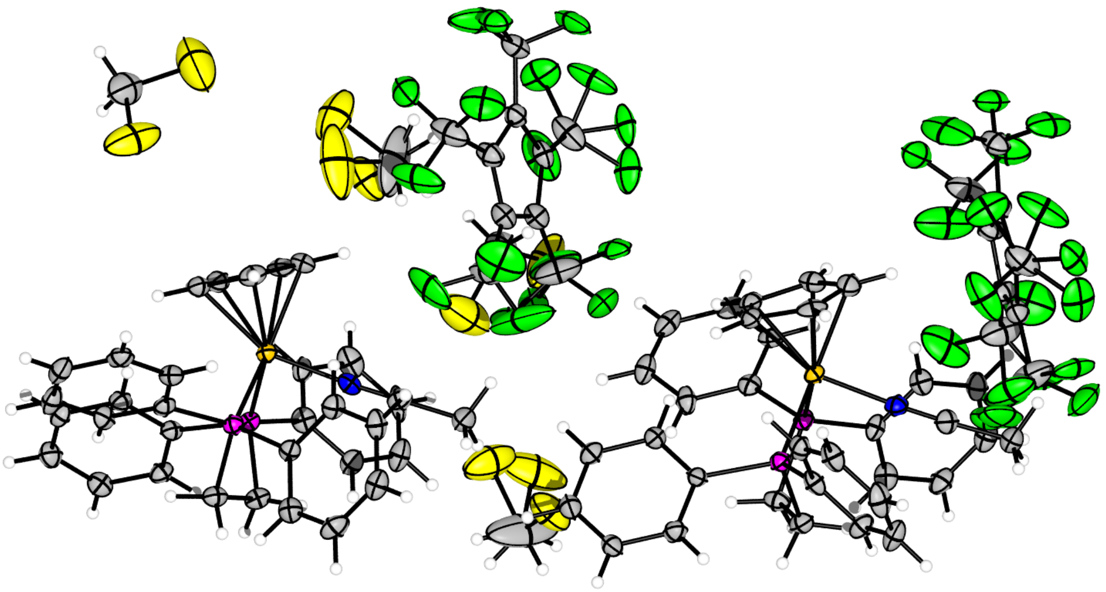

Figure S39. Molecular structure in the solid state of [Ru(C_5H_5)(DPPE)(MeCN)][$C_5(CF_3)_5$]· 2 CH_2Cl_2. Ellipsoids are depicted with 50% probability level. Color code: white-hydrogen, grey-carbon, green-fluorine, orange-iron, deep blue-nitrogen, yellow-chlorine, purple-phosphorus.

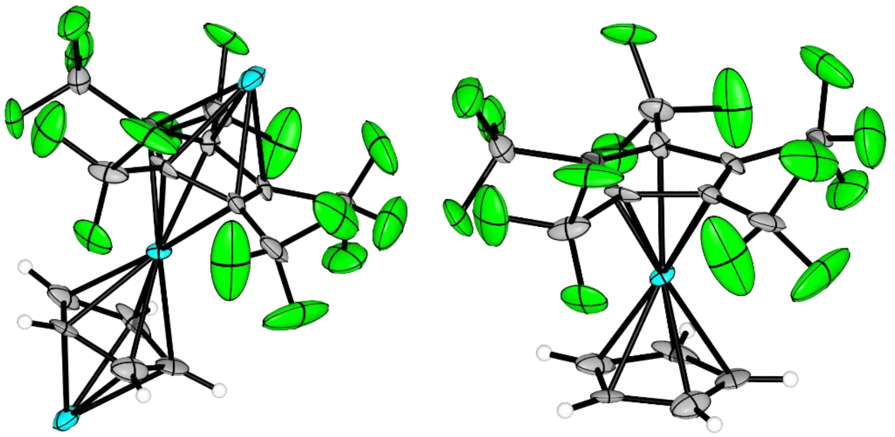

Figure S40. Molecular structure in the solid state of [Ru(C_5H_5)($C_5(CF_3)_5$)] with disorder (left) and without disorder (right). Ellipsoids are depicted with 50% probability level. Color code: white-hydrogen, grey-carbon, green-fluorine, light blue-ruthenium.

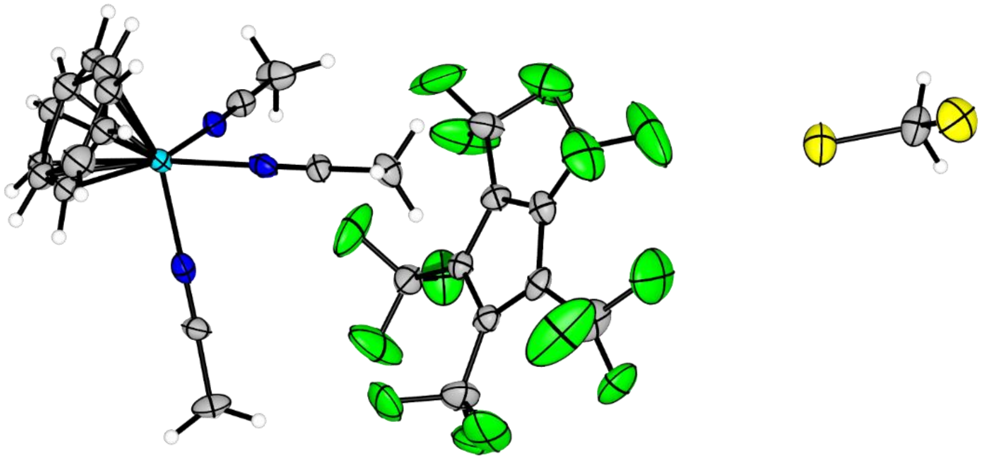

Figure S41. Molecular structure in the solid state of $[Ru(C_5H_5)(MeCN)_3][C_5(CF_3)_5]\cdot CH_2Cl_2$. Ellipsoids are depicted with 50% probability level. Color code: white-hydrogen, grey-carbon, green-fluorine, light blue-ruthenium, deep blue-nitrogen, yellow-chlorine.

$[Fe(C_5H_5)(DPPE)(MeCN)][C_5(CF_3)_5]$: This compound crystallized as a twin with a ratio of 97:3. The structure shows severe disorder, since solvent molecules as well as CF_3-groups are refined with split position which results in a higher number of parameters.

$[Ru(C_5H_5)(C_5(CF_3)_5)]$: The structure suffers from severe general and racemic twinning, being a four-fold twin. Further the Ru1 and Ru2 atoms are disordered (ratio 72:28) resulting in an infinite chain of Ru cations and $[C_5(CF_3)_5]^-$ anions. The above-average ADPs of six fluorine and two carbon atoms of the perfluorinated Cp* have been treated with the command ISOR.

$[Ru(C_5H_5)(MeCN)_3][C_5(CF_3)_5]$: The $[C_5H_5]^-$ ligand shows rotational disorder (ratio 58:42), that was treated with SADI commands. The compound is also twinned with a ratio of 53:47.

Density functional theory (DFT) calculations

$[C_5H_5]^-$

monoanionic, diamagnetic, HF= –193.6112471 Hartree

Table S4. Coordinates (x,y,z) for $[C_5H_5]^-$.

C	-0.919874000	-0.766354000	0.072425000
C	0.486217000	-0.686045000	-0.017904000
C	0.834871000	0.669451000	-0.199533000
C	-0.355783000	1.426887000	-0.221310000
C	-1.440310000	0.539512000	-0.053452000
H	1.177232000	-1.519183000	0.042606000
H	1.840763000	1.060232000	-0.303934000
H	-2.489063000	0.812971000	-0.025801000
H	-1.498637000	-1.672117000	0.214307000
H	-0.425222000	2.501582000	-0.346722000

$[C_5(CF_3)_5]^-$

monoanionic, diamagnetic, HF= –1879.679711 Hartree

Table S5. Coordinates (x,y,z) for $[C_5(CF_3)_5]^-$.

F	2.916325000	1.483320000	0.561188000
F	2.934289000	0.260199000	-1.234190000
F	2.196316000	2.277040000	-1.315684000
F	0.476555000	3.583212000	0.181258000
F	-0.253623000	3.219038000	-1.833661000
F	-1.638394000	3.436173000	-0.199647000
F	-3.403612000	1.555666000	-0.980693000
F	-3.709474000	-0.102251000	0.369742000
F	-3.067295000	1.787856000	1.155384000
F	-2.325795000	-2.290196000	1.310650000
F	-2.683058000	-2.079212000	-0.823208000
F	-0.949453000	-3.132654000	-0.130390000
F	1.004326000	-2.552439000	1.380332000
F	1.744487000	-2.601162000	-0.661775000
F	2.635941000	-1.282675000	0.804439000
C	1.449899000	-1.765095000	0.368256000
C	2.193723000	1.166010000	-0.546919000
C	-2.885988000	0.932754000	0.111934000
C	-1.709453000	-2.044883000	0.123955000
C	-0.927048000	-0.771878000	0.090431000
C	0.482120000	-0.678397000	0.026749000
C	-0.451478000	2.888708000	-0.527576000
C	0.818661000	0.664736000	-0.250237000
C	-0.376278000	1.418724000	-0.277940000
C	-1.457094000	0.532259000	-0.057083000

$[Fe(C_5H_5)]^+$

monocationic, diamagnetic, HF = –1457.0535874 Hartree

Table S6. Coordinates (x,y,z) for $[Fe(C_5H_5)]^+$.

Fe	-0.9306960000	-0.0001230000	-0.0023450000
C	0.6991630000	1.1943230000	-0.2072250000
C	0.7018810000	0.1703660000	-1.1983350000
C	0.6956660000	0.5679120000	1.0728100000
C	0.6964540000	-0.8432650000	0.8726140000
C	0.7002480000	-1.0889430000	-0.5310820000
H	0.6507610000	-2.0571640000	-1.0053630000
H	0.6432000000	-1.5924840000	1.6470900000
H	0.6409970000	1.0721170000	2.0251600000
H	0.6483790000	2.2561010000	-0.3930820000
H	0.6542780000	0.3222730000	-2.2655460000

$[Fe(C_5H_5)]^+$

Monocationic, paramagnetic (quintet), HF= –1457.0945932 Hartree

Table S7. Coordinates (x,y,z) for $[Fe(C_5H_5)]^+$ (quintet)

Fe	-1.168373000	-0.013711000	0.067300000
C	0.696382000	1.194132000	-0.216035000
C	0.713508000	0.171467000	-1.202138000
C	0.672752000	0.572071000	1.068166000
C	0.672733000	-0.845327000	0.867308000
C	0.695880000	-1.085687000	-0.539773000
H	0.707106000	-2.053477000	-1.016551000
H	0.691712000	-1.596802000	1.641051000
H	0.690205000	1.078795000	2.020372000
H	0.705773000	2.256415000	-0.404601000
H	0.722655000	0.323237000	-2.270402000

$[Ru(C_5H_5)]^+$

monocationic, diamagnetic, HF = –288.2832530 Hartree

Table S8. Coordinates (x,y,z) for $[Ru(C_5H_5)]^+$.

Ru	-0.7647490000	-0.0008190000	-0.0005690000
C	0.9636920000	1.0576540000	-0.6063440000
C	0.9664190000	-0.2497740000	-1.1915970000
C	0.9620780000	0.9048970000	0.8179550000
C	0.9642850000	-0.4970420000	1.1128000000
C	0.9676320000	-1.2106750000	-0.1291770000
H	0.9454500000	-2.2833210000	-0.2446370000
H	0.9388120000	-0.9373600000	2.0973090000
H	0.9366920000	1.7049960000	1.5414160000
H	0.9398730000	1.9931630000	-1.1437570000
H	0.9435130000	-0.4718250000	-2.2471310000

$[Fe(C_5(CF_3)_5)]^+$

monocationic, diamagnetic, HF = –3142.9555470 Hartree

Table S9. Coordinates (x,y,z) for $[Fe(C_5(CF_3)_5)]^+$.

Fe	-0.034741000	0.020995000	1.321412000
F	0.828871000	3.154125000	0.681656000
F	0.356557000	3.219606000	-1.450986000
F	-1.220725000	3.259414000	0.016544000
F	-2.599832000	1.412716000	1.201678000
F	-3.073814000	1.614031000	-0.929233000
F	-3.449682000	-0.237674000	0.112913000
F	-0.819153000	-3.277565000	-0.932793000
F	-2.115733000	-2.620564000	0.681239000
F	-2.515085000	-2.046149000	-1.385836000
F	0.968429000	-2.992530000	0.878123000
F	1.954256000	-2.819289000	-1.061763000
F	2.727784000	-1.777388000	0.677912000
F	3.057482000	0.964670000	0.930563000
F	3.335141000	0.049503000	-1.031996000
F	2.686007000	2.091364000	-0.884830000
C	-1.562467000	-2.270791000	-0.485151000
C	-2.620055000	0.787413000	-0.005990000
C	-0.029347000	2.751079000	-0.270860000
C	2.589384000	0.893740000	-0.325927000
C	1.622593000	-2.169473000	0.038232000
C	0.725861000	-0.964197000	-0.256666000
C	1.134770000	0.405112000	-0.308229000
C	-0.044302000	1.220350000	-0.304213000
C	-0.706751000	-1.006261000	-0.325838000
C	-1.182702000	0.342161000	-0.295233000

[Fe(C$_5$(CF$_3$)$_5$)]$^+$

monocationic, paramagnetic (quintet), HF = –3143.0017633 Hartree

Table S10. Coordinates (x,y,z) for [Fe(C$_5$(CF$_3$)$_5$)]$^+$.

Fe	-0.192275000	0.230905000	1.569425000
F	0.864711000	3.177288000	0.507558000
F	0.315870000	3.207141000	-1.604660000
F	-1.205588000	3.270929000	-0.073678000
F	-2.272760000	1.101050000	1.429009000
F	-3.137694000	1.760599000	-0.517515000
F	-3.414593000	-0.253309000	0.142886000
F	-0.802450000	-3.229336000	-1.129598000
F	-2.069970000	-2.690354000	0.553841000
F	-2.541281000	-2.014784000	-1.463514000
F	0.894140000	-3.076768000	0.797728000
F	2.087497000	-2.711678000	-0.983578000
F	2.589916000	-1.759191000	0.907691000
F	3.025124000	1.086609000	0.940352000
F	3.327790000	-0.046535000	-0.894198000
F	2.682490000	1.999981000	-1.000743000
C	-1.550162000	-2.261610000	-0.607684000
C	-2.563545000	0.736944000	0.039813000
C	-0.026146000	2.748487000	-0.409064000
C	2.566784000	0.873391000	-0.306854000
C	1.586542000	-2.157715000	0.107110000
C	0.689650000	-0.963118000	-0.203694000
C	1.111192000	0.405697000	-0.260588000
C	-0.053263000	1.222572000	-0.396182000
C	-0.712925000	-0.999468000	-0.401878000
C	-1.186307000	0.346676000	-0.457258000

[Ru(C$_5$(CF$_3$)$_5$)]$^+$

monocationic, diamagnetic, HF = –1974.1852318 Hartree

Table S11. Coordinates (x,y,z) for [Ru(C$_5$(CF$_3$)$_5$)]$^+$.

Ru	-0.0022390000	0.0141820000	1.3754410000
F	2.8459840000	1.7260370000	0.5567120000
F	2.4989770000	1.9550590000	-1.5891120000
F	1.4449310000	3.1945210000	-0.1794220000
F	-0.8094100000	3.0469880000	0.9633780000
F	-1.1528750000	3.1980610000	-1.1881280000
F	-2.6100570000	2.2535720000	0.1085070000
F	-2.8870610000	-1.7222410000	-1.0529390000
F	-3.3915020000	-0.3713190000	0.5690000000
F	-3.1859750000	0.3585730000	-1.4775280000
F	-1.4415780000	-2.8896140000	0.7028510000
F	-0.5748860000	-3.3252040000	-1.2510070000
F	0.6709400000	-3.2393360000	0.5245060000
F	2.8474090000	-1.5863290000	0.8294870000
F	2.3820660000	-2.2859890000	-1.1848480000
F	3.4015710000	-0.4147510000	-0.9037030000
C	-2.7123760000	-0.4953720000	-0.5724480000
C	-1.3137270000	2.4304440000	-0.1210640000
C	1.9296220000	1.9785610000	-0.3878980000
C	2.4720720000	-1.2138800000	-0.4016570000
C	-0.3818960000	-2.7055040000	-0.0990730000
C	-0.1671960000	-1.2029960000	-0.3039840000
C	1.1016950000	-0.5185510000	-0.3416210000
C	0.8399140000	0.9021160000	-0.3344550000
C	-1.2157090000	-0.2139370000	-0.3584880000
C	-0.5937810000	1.0880740000	-0.3075100000

[Fe(C_5H_5)($C_5(CF_3)_5$)]
neutral, diamagnetic, HF = –3336.9986336 Hartree
Table S12. Coordinates (x,y,z) for [Fe(C_5H_5)($C_5(CF_3)_5$)].

Fe	-0.0143280000	0.0145610000	1.0739940000
F	2.8934940000	1.9161850000	-0.9528750000
F	3.2456390000	-0.1077100000	-1.6024490000
F	3.3098640000	0.3971790000	0.5109680000
F	2.6560080000	-2.1074200000	0.0661530000
F	1.6197190000	-2.8551550000	-1.6775450000
F	0.8278730000	-3.2080980000	0.3173530000
F	-2.6633300000	-1.7241010000	-1.7526030000
F	-2.3789090000	-2.4769270000	0.2689450000
F	-1.1072420000	-3.1427080000	-1.3488640000
F	-3.4081880000	0.0467780000	-0.0832200000
F	-2.9664270000	1.5035190000	-1.6283660000
F	-2.6550800000	1.9715880000	0.4697810000
F	-0.9687310000	3.3583960000	-0.7028570000
F	0.7713010000	3.0325040000	-1.9159790000
F	0.9684040000	3.2716650000	0.2409210000
C	-1.1320400000	0.4634390000	-0.5618090000
C	-0.7785540000	-0.9257400000	-0.6003140000
C	0.0820760000	1.2298160000	-0.5820870000
C	1.1834060000	0.3110590000	-0.5671920000
C	0.6482550000	-1.0184430000	-0.5523210000
C	1.4408620000	-2.3023380000	-0.4686250000
C	2.6624840000	0.6365700000	-0.6450770000
C	0.2107660000	2.7299710000	-0.7311030000
C	-2.5429290000	1.0029600000	-0.4573160000
C	-1.7383080000	-2.0745140000	-0.8419740000
C	1.0071120000	0.6284140000	2.7682530000
C	-0.2980400000	1.1920180000	2.7551790000
C	0.8752460000	-0.7871140000	2.7650440000
C	-1.2367180000	0.1251450000	2.7421350000
C	-0.5113490000	-1.0980060000	2.7488310000
H	1.9334940000	1.1762040000	2.7362080000
H	-0.5330360000	2.2422140000	2.7107750000
H	1.6843350000	-1.4971890000	2.7308490000
H	-2.3076280000	0.2249920000	2.6888280000
H	-0.9378160000	-2.0854880000	2.6954830000

[Ru(C_5H_5)($C_5(CF_3)_5$)]
neutral, diamagnetic, HF = –2168.2185621 Hartree
Table S13. Coordinates (x,y,z) for [Ru(C_5H_5)($C_5(CF_3)_5$)].

Ru	-0.515119000	0.069216000	1.234657000
F	-3.089389000	1.984307000	2.845318000
F	-2.592460000	1.468671000	4.903017000
F	-1.435792000	2.937974000	3.846829000
F	0.685310000	2.974954000	2.559975000
F	1.304648000	2.308530000	4.531816000
F	2.388313000	1.693572000	2.755172000
F	2.264013000	-2.411600000	3.250817000
F	2.909398000	-0.868880000	1.879817000
F	2.799262000	-0.481951000	4.017917000
F	0.604533000	-3.228595000	1.491734000
F	-0.169995000	-3.719327000	3.463401000
F	-1.517136000	-3.353722000	1.811132000
F	-3.487152000	-1.588642000	1.857647000
F	-2.982220000	-2.371152000	3.821775000
F	-3.848070000	-0.408526000	3.619192000
C	0.509577000	-0.013279000	-0.708697000
C	0.178289000	1.343094000	-0.419981000

C	-0.704043000	-0.756083000	-0.795534000
C	-1.240254000	1.438259000	-0.327686000
C	-1.785536000	0.140972000	-0.558728000
C	2.189792000	-1.098868000	2.989553000
C	1.163105000	1.926449000	3.251880000
C	-2.072182000	1.771977000	3.698840000
C	-2.983004000	-1.260699000	3.060842000
C	-0.369044000	-2.953936000	2.378975000
C	-0.400045000	-1.483720000	2.730020000
C	-1.577868000	-0.694004000	2.973633000
C	-1.158253000	0.659019000	3.232347000
C	0.748917000	-0.635229000	2.880420000
C	0.279298000	0.701875000	3.133840000
H	1.504434000	-0.410984000	-0.819188000
H	0.877295000	2.150121000	-0.278697000
H	-0.787791000	-1.812481000	-0.987478000
H	-1.801934000	2.329490000	-0.103398000
H	-2.829967000	-0.120369000	-0.536100000

$[Rh(COD)]^+$

cationic, diamagnetic, HF= −422.5845527 Hartree

Table S14. Coordinates (x,y,z) for $[Rh(COD)]^+$.

Rh	-0.383380000	-0.200454000	1.936381000
C	0.941295000	0.122912000	0.335474000
C	0.419023000	1.124182000	-0.677974000
C	0.541964000	-1.237363000	0.384945000
C	-0.794128000	1.896798000	-0.130178000
C	-0.483673000	-1.887805000	-0.509082000
C	-1.772156000	-1.054475000	-0.627771000
C	-2.036360000	-0.260427000	0.637967000
C	-1.608542000	1.077137000	0.838935000
H	-2.615413000	-1.717055000	-0.814725000
H	-1.720644000	-0.372089000	-1.475162000
H	-0.719295000	-2.864927000	-0.087628000
H	-0.047236000	-2.077753000	-1.495202000
H	1.894735000	0.367744000	0.798153000
H	1.231057000	-1.926971000	0.871838000
H	1.212961000	1.826402000	-0.926137000
H	0.167250000	0.602693000	-1.600494000
H	-0.451421000	2.788099000	0.395218000
H	-1.434626000	2.249974000	-0.945047000
H	-2.868346000	-0.602759000	1.249238000
H	-2.158343000	1.655045000	1.581381000

$[Rh(C_5(CF_3)_5)(COD)]$

neutral, diamagnetic, HF= −2302.4545927 Hartree

Table S15. Coordinates (x,y,z) for $[Rh(COD)]^+$.

Rh	0.626018000	-0.718569000	0.187351000
F	-2.755850000	-1.670257000	1.199520000
F	-3.523272000	0.337734000	1.507615000
F	-3.781057000	-0.631243000	-0.391832000
F	-2.121934000	-1.765221000	-2.050195000
F	-2.851354000	0.166121000	-2.742465000
F	-0.945119000	-0.579473000	-3.407255000
F	1.327387000	0.315663000	-3.067474000
F	0.204876000	2.166397000	-3.204586000
F	2.018182000	2.044854000	-2.016684000
F	-1.437736000	0.018133000	3.034299000
F	0.296338000	1.285374000	3.084384000
F	-1.667172000	2.162643000	2.800713000

F	2.372056000	2.469789000	0.674709000
F	0.980164000	3.729360000	-0.423999000
F	0.655565000	3.342189000	1.661113000
C	-0.892397000	1.117994000	2.477516000
C	1.070538000	2.772460000	0.519849000
C	0.904936000	1.383129000	-2.365500000
C	-1.754001000	-0.503443000	-2.344413000
C	-2.917091000	-0.466836000	0.616176000
C	-1.613494000	0.149777000	0.152548000
C	-1.107969000	0.176491000	-1.159919000
C	0.076264000	1.006316000	-1.155771000
C	-0.744298000	0.946498000	0.982856000
C	0.215501000	1.583012000	0.150161000
C	1.470247000	-1.630740000	1.960487000
C	2.939147000	-1.987154000	1.816492000
C	0.419865000	-2.423865000	1.477453000
C	3.559381000	-1.339625000	0.572665000
C	0.567734000	-3.702126000	0.688209000
C	1.605923000	-3.585537000	-0.435529000
C	1.669538000	-2.175750000	-0.999288000
C	2.570669000	-1.189605000	-0.557380000
H	1.350272000	-4.276767000	-1.238927000
H	2.594565000	-3.881348000	-0.083867000
H	-0.405782000	-3.928899000	0.251371000
H	0.805048000	-4.536148000	1.359755000
H	1.236123000	-0.959744000	2.776867000
H	-0.538983000	-2.309133000	1.968361000
H	3.473233000	-1.651744000	2.705815000
H	3.049255000	-3.071240000	1.786276000
H	3.912580000	-0.339863000	0.829127000
H	4.438921000	-1.899779000	0.232644000
H	1.292020000	-2.062225000	-2.008653000
H	2.834673000	-0.418861000	-1.271150000

References

[1] H. E. Gottlieb, V. Kotlyar, A. Nudelman, *J. Org. Chem.* **1997**, *62*, 7512–7515.

[2] G. R. Fulmer, A. J. M. Miller, N. H. Sherden, H. E. Gottlieb, B. M. Stoltz, J. E. Bercaw, K. I. Goldberg, *Organometallics* **2010**, *29*, 2176–2179.

[3] R. K. Harris, E. D. Becker, S. M. Cabral de Menezes, R. Goodfellow, P. Granger, *Pure Appl. Chem.* 2001, *73*, 1795–1818.

[4] M. R. Willcott, *J. Am. Chem. Soc.*, 2009, **131**, 13180.

[5] O. V. Dolomanov, L. J. Bourhis, R. J. Gildea, J. A. K. Howard, H. Puschmann, *J. Appl. Cryst.*, 2009, **42**, 339–341.

[6] G. M. Sheldrick, *Acta Cryst.*, 2015, **A71**, 3–8.

[7] G. M. Sheldrick, *SHELXL Version 2014/7, Program for Crystal Structure Solution and Refinement*; Göttingen, Germany, 2014.

[8] G. M. Sheldrick, *Acta Cryst.*, 2008, **A64**, 112–122.

[9] K. Brandenburg, Diamond: Crystal and Molecular Structure Visualization http://www.crystalimpact.com/diamond

[10] Persistence of Vision Pty. Ltd. Persistence of Vision Raytracer. Ltd., Persistence of Vision Pty. 2004.

[11] Gaussian 16, Revision C.01, M. J. Frisch, G. W. Trucks, H. B. Schlegel, G. E. Scuseria, M. A. Robb, J. R. Cheeseman, G. Scalmani, V. Barone, G. A. Petersson, H. Nakatsuji, X. Li, M. Caricato, A. V. Marenich, J. Bloino, B. G. Janesko, R. Gomperts, B. Mennucci, H. P. Hratchian, J. V. Ortiz, A. F. Izmaylov, J. L. Sonnenberg, D. Williams-Young, F. Ding, F. Lipparini, F. Egidi, J. Goings, B. Peng, A. Petrone, T. Henderson, D. Ranasinghe, V. G. Zakrzewski, J. Gao, N. Rega, G. Zheng, W. Liang, M. Hada, M. Ehara, K. Toyota, R. Fukuda, J. Hasegawa, M. Ishida, T. Nakajima, Y. Honda, O. Kitao, H. Nakai, T. Vreven, K. Throssell, J. A. Montgomery, Jr., J. E. Peralta, F. Ogliaro, M. J. Bearpark, J. J. Heyd, E. N. Brothers, K. N. Kudin, V. N. Staroverov, T. A. Keith, R. Kobayashi, J. Normand, K. Raghavachari, A. P. Rendell, J. C. Burant, S. S. Iyengar, J. Tomasi, M. Cossi, J. M. Millam, M. Klene, C. Adamo, R. Cammi, J. W. Ochterski, R. L. Martin, K. Morokuma, O. Farkas, J. B. Foresman, and D. J. Fox, Gaussian, Inc., Wallingford CT, 2016.

[12] M. D. Hanwell, D. E. Curtis, D. C. Lonie, T. Vandermeersch, E. Zurek, G. R. Hutchison, *J. Cheminformatics* **2012**, *4*, 17.

[13] G. A. Zhurko, in *ChemCraft*, http://www.chemcraftprog.com

[14] E. D. Laganis, D. M. Lemal, *J. Am. Chem. Soc.* **1980**, *102*, 6633.

[15] R. D. Chambers, W. K. Gray, J. F. S. Vaughan, S. R. Korn, M. Médebielle, A. S. Batsanov, C. W. Lehmann, J. A. K. Howard, *Perkin Trans.*, **1997**, 135–146.

[16] R. Sievers, M. Sellin, S. M. Rupf, M. Malischewski, *Angew. Chem. Int. Ed.*, **2022**, *e202211147*.

[17] C. Malchau, N. Ultes, J. Ehrhard, T. R. Eger, D. V. Fries, B. Oelkers, S. Becker, G. Niedner Schatteburg, W. R. Thiel, *J. Organomet. Chem.* **2023**, *987*, 122618.

[18] R. Sievers, N. G. Kub, T.-N. Streit, M. Reimann, G. Thiele, M. Kaupp, M. Malischewski, ChemRxiv 2025, DOI: 10.26434/chemrxiv-2025-lqllp

[19] $[\mathbf{C_5}(CF_3)_5]^-$ resonances are suppressed and invisible in $^{13}C\{^{19}F\}$ NMR spectroscopy even at prolonged measurement times.

[20] Me**C**N resonances are suppressed and invisible in $^{13}C\{^{1}H\}$ NMR spectroscopy even at prolonged measurement times.
